# A toolbox of molecular photoswitches to modulate the CXCR3 chemokine receptor with light

**DOI:** 10.3762/bjoc.15.244

**Published:** 2019-10-23

**Authors:** Xavier Gómez-Santacana, Sabrina M de Munnik, Tamara A M Mocking, Niels J Hauwert, Shanliang Sun, Prashanna Vijayachandran, Iwan J P de Esch, Henry F Vischer, Maikel Wijtmans, Rob Leurs

**Affiliations:** 1 Division of Medicinal Chemistry, Amsterdam Institute for Molecules Medicines and Systems (AIMMS), Vrije Universiteit Amsterdam, 1081 HZ, Amsterdam, The Netherlands; 2 present address: Institute of Functional Genomics, Université de Montpellier, Unité 5302 CNRS and Unité U1191, INSERM, 34090 Montpellier, France

**Keywords:** azo compounds, chemokine receptor, efficacy photoswitching, G protein-coupled receptors, photopharmacology

## Abstract

We report a detailed structure–activity relationship for the scaffold of VUF16216, a compound we have previously communicated as a small-molecule efficacy photoswitch for the peptidergic chemokine GPCR CXCR3. A series of photoswitchable azobenzene ligands was prepared through various synthetic strategies and multistep syntheses. Photochemical and pharmacological properties were used to guide the design iterations. Investigations of positional and substituent effects reveal that halogen substituents on the *ortho*-position of the outer ring are preferred for conferring partial agonism on the *cis* form of the ligands. This effect could be expanded by an electron-donating group on the *para*-position of the central ring. A variety of efficacy differences between the *trans* and *cis* forms emerges from these compounds. Tool compounds VUF15888 (**4d**) and VUF16620 (**6e**) represent more subtle efficacy switches, while VUF16216 (**6f**) displays the largest efficacy switch, from antagonism to full agonism. The compound class disclosed here can aid in new photopharmacology studies of CXCR3 signaling.

## Introduction

Photopharmacology is an emerging discipline at the interface of medicinal chemistry and photochemistry. Classical medicinal chemistry approaches make use of small-molecule ligands binding a target protein, thereby modifying its activity. Photopharmacological approaches use light-sensitive photochromic ligands that provide an advantageous and more precise pharmacological alternative, especially with respect to spatial and temporal precision [1–2]. Photochromic ligands usually contain a molecular photoswitch (photoswitchable moiety) that under certain wavelengths of illumination undergoes an isomerization event, thereby changing the properties of the molecule and the binding affinity for the target protein [3–5] or the intrinsic functional activity (efficacy) [6–7]. Despite the considerable number of photoswitches reported to date, such as spiropyrans, diarylethenes, fulgides or azobenzenes, the most widely used moiety in the photopharmacology is the latter one. One of the main reasons is that an azobenzene has a relatively simple structure that can resemble various biaryl moieties of bioactive compounds: two aromatic rings linked with a bridge (e.g., amide, ether, alkane or alkyne) [8]. In the case of azobenzene the bridge is a diazene group (also called azo group) and depending on the wavelength of illumination, a linear *trans-*isomer or a bent *cis-*isomer can be obtained [9]. If certain biaryl moieties are replaced by an azobenzene (i.e., azologization approach), there is a relatively good chance that one of the resulting photoisomers will have a spatial disposition similar to the original biaryl unit and, therefore, a similar biological activity that might change upon isomerization of the azobenzene [8]. The second reason for the success of azobenzene in the photopharmacology field is the robust photoisomerization. It provides typically high yields of photoisomerization with relatively low intensity of light and minimal photobleaching even over hundreds of cycles. A third reason is the relatively high synthetic accessibility to azobenzenes. All these properties make azobenzene compounds ideal molecular photoswitches to control protein activity and physiological events with light.

**Figure 1:**
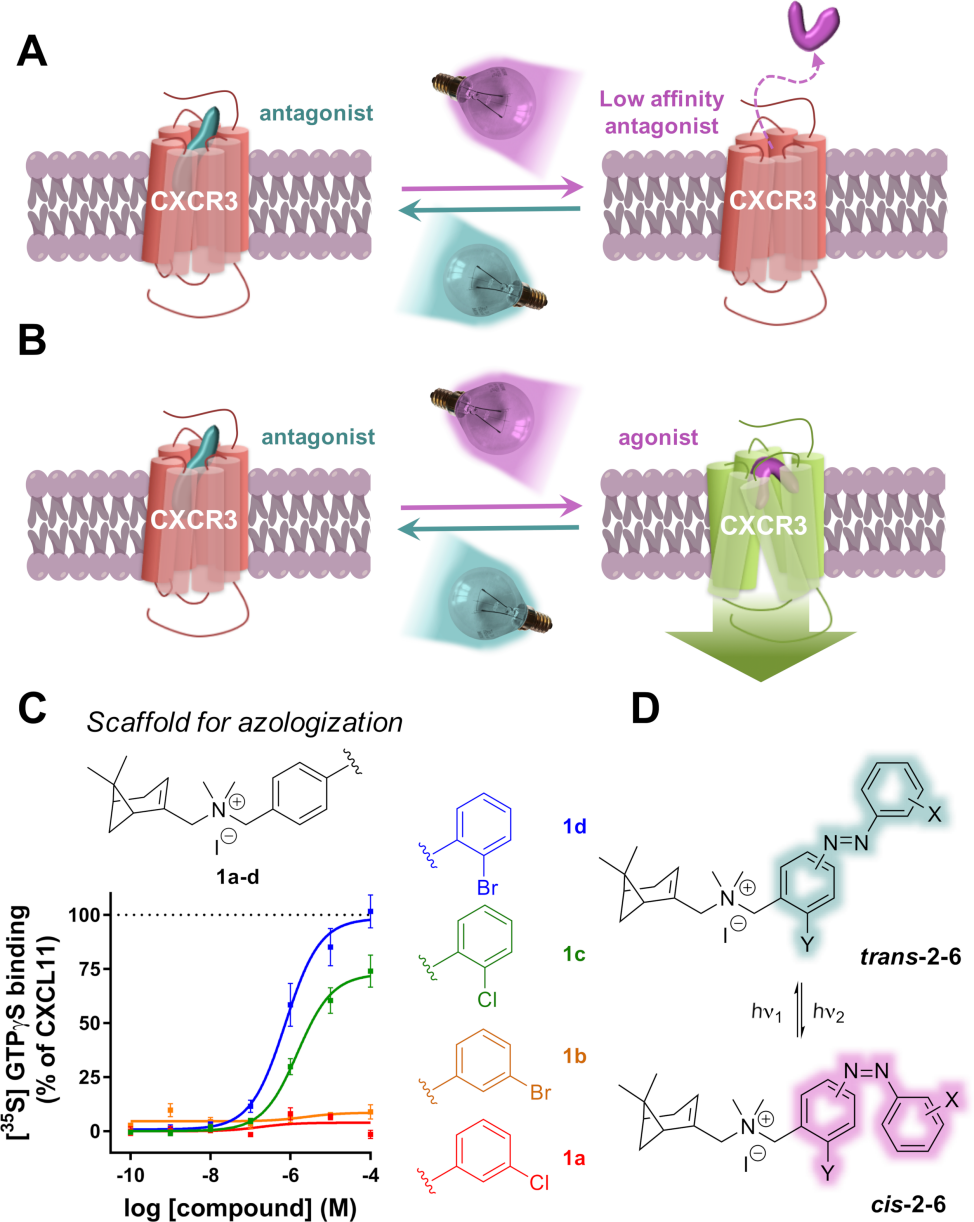
Design of the CXCR3 efficacy photowitchable ligands. A,B) Schematic representation of a GPCR photochromic ligand that photoisomerizes and thereby photoswitches (A) binding affinity and/or (B) functional efficacy. Red represents the inactive GPCR, while green represents the active GPCR. C) General structure and exemplary functional dose-response curves of the parent biaryl family of CXCR3 ligands disclosed in Wijtmans et al. [24], in which *ortho* substitution on the outer aromatic ring gives partial or full agonists, while *meta* substitution provides antagonists. D) Azologization of the biaryl moiety provides a family of photowitchable CXCR3 small-molecule ligands.

A number of protein targets have been explored with photochromic small-molecule ligands, such as ion channels, microtubules, enzymes and GPCRs (G protein-coupled receptors) [1,10]. We focus our photopharmacology research on GPCRs [3,7,11], which constitute a superfamily of membrane proteins that regulate many physiological processes [12]. Despite the high relevance of GPCRs both functionally and as a drug target [12], the first synthetic GPCR photochromic small-molecule ligands appeared only five years ago [13–15]. Since then, photopharmacology has been explored on GPCRs targeted endogenously by small molecules [3–4,11,16–20], small peptides [5,13] and larger peptides [7,21–22]. Most of the targeted GPCRs belong to the three rhodopsin-, secretin- and glutamate-like subfamilies and involve GPCRs that endogenously bind small-molecule ligands [10]. The ensuing photochromic GPCR ligands are usually orthosteric and the photoswitching generally affects the functional potency [4,11,23] and/or the binding affinity [3–5,11] of the ligand (Figure 1A). However, as mentioned, GPCRs that endogenously bind large molecules (large peptides or proteins) can be also targeted by allosteric photochromic ligands. In an initial communication [7], we recently reported a photochromic ligand class that is based on azologization of a biaryl ligand class [24] and that activates the chemokine CXCR3 receptor (CXCR3), a GPCR endogenously activated by large peptides CXCL9, CXCL10 and CXCL10 and involved in inflammatory responses. In fact, the six compounds reported in that study represent the first photochromic small-molecule class that harbors a dynamic efficacy photoswitch (from antagonism to agonism) on a peptidergic GPCR (Figure 1B). Here, we report the rationale and synthetic strategies behind this series of compounds, a detailed analysis of the molecular determinants that control the efficacy of the ligands (SAR, structure-activity relationship) and a toolbox of pharmacologically useful photoswitchable small-molecule CXCR3 agonists.

## Results and Discussion

### Azologization design

**Figure 2:**
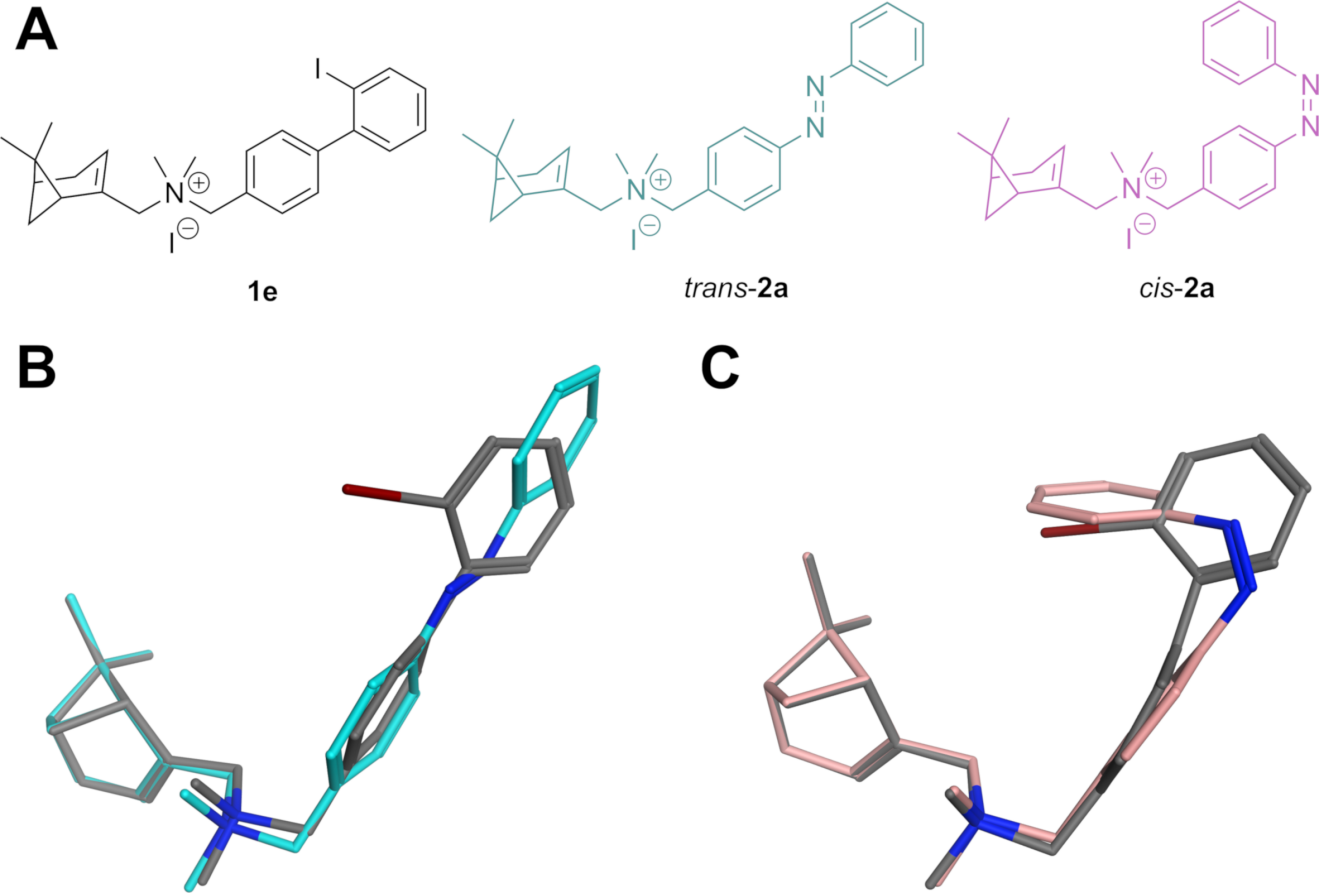
Conformational alignment of a biaryl CXCR3 agonist with a designed azobenzene analogue. A) 2D structure of biaryl CXCR3 agonist **1e** and designed ligand *trans*-**2a** and *cis*-**2a**. B,C) Alignments of **1e** (grey carbon atoms) and **2a**, in which the *trans* and *cis*-isomers are shown in (B) turquoise and (C) light magenta carbon atoms, respectively. The iodine atom is shown in red for clarity.

The chemokine receptor CXCR3 is endogenously activated by the chemotactic peptides CXCL11, CXCL10 and CXCL9. Synthetic small-molecule ligands can also bind to CXCR3 [25]. Multiple small-molecule CXCR3 antagonist scaffolds have been disclosed but small-molecule CXCR3 agonists are scarce and are mostly limited to peptidomimetics [25], which makes our published biaryl series a notable exception [24]. The general scaffold of these biaryl ligands consists of a polycycloaliphatic anchor and a biaryl moiety both linked to an ammonium ion. Depending on the substitution pattern of this biaryl moiety, a broad spectrum of efficacies for CXCR3 can be obtained, i.e., from antagonists to partial agonists and full agonists (Figure 1C) [24]. *Meta* and *para*-substitution yields antagonists (exemplified by **1a**,**b**), while o*rtho*-substitution with halogen atoms provides agonists, exemplified by partial agonist **1c** and equal full agonists **1d** and **1e** (VUF11418, Figure 2A). A tentative explanation for this efficacy switch includes a variation of the dihedral angle of the biaryl moiety, an increase of the electron density in the biaryl unit and/or a postulated halogen bond of the halogen substituent to the binding site of the receptor [24].

In order to obtain an efficacy photoswitching, we opted for replacing the biaryl moiety for an azobenzene in an azologization approach (Figure 1D) with the expectation that the isomerization of the azobenzene would provide changes in 3D shape that are similar to those observed in the biaryl series. To reinforce this hypothesis, molecular alignments were performed with Molecular Operating Environment (MOE) software [26] in which **1e** was used as a model for full agonism (Figure 2). Its 3D structure was superposed with both the *trans* and *cis-*isomers of parent azobenzene compound **2a** allowing flexibility of the molecules except for the conformation of the *trans* and *cis*-azobenzene moieties, which were fixed in the lowest energy conformation to ensure a shape that has also been validated by crystallographic data [27]. The results show a reasonable overall alignment between *trans*-**2a** and the agonist **1e** (Figure 2B), since the planar azobenzene is partially overlapping with the biaryl moiety. However, the two aromatic rings of both compounds can evidently not be in exactly the same plane because the azobenzene moiety is planar while the tilting of the dihedral angle of the biaryl moiety of **1e** was speculated to be associated with its agonist activity (vide supra) [24]. The alignment of the *cis-*isomer of **2a** with agonist **1e** is very different. The outer aromatic ring of *cis-***2a** goes out of plane and is now occupying the space that is also occupied by the iodine atom of **1e**. These calculations indicate that CXCR3 agonism is more likely to be associated with the *cis-*isomer than with the *trans-*isomer in our designed azobenzenes.

### Synthesis of azobenzene analogues and exploration of substitution pattern on the outer aromatic ring

**Scheme 1:**
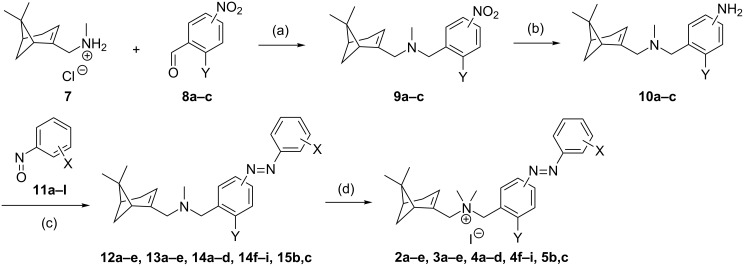
Synthetic strategies for compounds **2a–e**, **3a–e**, **4a–d**, **4f–i** and **5b,c** (Y = H, Cl). Reagents and conditions: (a) i) Et_3_N (1.1–1.6 equiv), DCM, rt, 10–30 min; ii) NaBH(OAc)_3_ (1.6 equiv), rt, 6–16 h, 91–97%; (b) SnCl_2_ (5.0 equiv), EtOH, 75 °C, 2 h, 87%–quant; (c) AcOH/DCM, rt, 1–5 d, 23–73%, (d) MeI (20 equiv), DCM, rt, 6–72 h, 41–97%.

In addition to unsubstituted azobenzene analogue **2a,** we explored the substitution pattern of the outer aromatic ring with chlorine atoms in the *ortho*, *meta* and *para*-position (compounds **2b–d**, respectively) to also assess the possibility of agonism provided by a halogen bond. Compound **2e**, which contains a bromine atom in the *ortho*-position, was also tested since this atom type provides full agonist activity of parent **1d**. The synthesis of the compounds **2a–e** was performed according to the strategies depicted in Scheme 1. The intermediate **7** was prepared as described previously by us [28] and was used in a reductive amination with 4-nitrobenzaldehyde (**8a**) to give the corresponding tertiary amine **9a** in high yield. The nitro group of **9a** was subsequently reduced by SnCl_2_ in high yield. The resulting aniline **10a** was used to obtain the azo compounds **12a–e** in varying yields through a Mills reaction with the corresponding nitroso compounds **11a–e**, which were commercially available or prepared as described in our previous communication [7]. A final methylation of the tertiary amine **12a–e** with MeI in DCM and subsequent precipitation with MTBE (methyl *tert*-butyl ether) gave **2a–e** as orange powders with ≥99% *trans*-isomer in moderate to high yield.

Next, we characterized the photochemical properties of **2a–e**, including absorption maxima, wavelengths of illumination and percentage of conversion from *trans* to *cis* in the photostationary state (PSS *cis*). First, UV–vis absorption spectra were measured in the dark and after illumination with a wavelength of 360 nm. In all cases the spectrum measured in the dark shows a large band between 320 and 330 nm that corresponds to the π–π* transition of the *trans-*isomer (Figure S1A, Supporting Information File 1). After irradiating with different wavelengths, the proportion of *trans* and *cis-*isomer varies to reach the PPS. For example, after irradiating with 360 nm the π–π* transition band of the *trans-*isomer can barely be observed, but a less intense wide band around 420 nm appears, which corresponds to the n–π* transition of the *cis-*isomer (Figure S1A, Supporting Information File 1). This indicates that, using 360 nm light, a PPS of high percentage of *cis-*isomer (PPS_360_) can be reached. Literature evidence [7,29–30] suggests that this scaffold would have sufficiently long half-lives of the PSS state, i.e., the *cis*-isomer only slowly reverts to *trans* in the dark, which was confirmed by early-stage analyses on key compounds (data not shown). In fact, due to this bistable nature, the percentage of each photoisomer can be quantified by analytical chromatography (LC–MS). The integration wavelength (265 nm) was selected closely to the observed isosbestic point for most compounds to get an indication of mole ratios from the UV area ratios. For **2a–e** after illumination at 360 nm, PPS_360_ values of 79–90% of *cis-*isomer were obtained. We also tested 434, 460 nm and/or 494 nm to revert the isomerization process to PSS *trans* (Figure S1A, Supporting Information File 1). After re-illuminating the samples at 434 nm or 460 nm, the π–π* transition band of the *trans-*isomer re-appears, indicating a high percentage of *trans-*isomer at that PSS. The use of 494 nm affords less *trans* compound and is less efficient in achieving PSS *trans*.

Next, the CXCR3 binding properties of **2a–e** were measured (Table 1) in a competition binding assay versus displacement of a radiolabeled small-molecule CXCR3 antagonist ([^3^H]-VUF11211 [31]). Values reported are mean ± SEM (Standard Error of the Mean). These experiments were performed with samples under dark conditions to ensure ≥99% *trans-*isomer and with samples previously illuminated with 360 nm light to obtain a high percentage of *cis* compound in the PSS_360_. This assay setup is enabled by the bistable nature of the photoswitch (vide supra), maintaining integrity of both the *trans* and *cis*-isomer for the duration of the assay. Compounds **2a–e** under dark conditions (*trans-*isomers) bind CXCR3 with *K*_i_ values in the high nanomolar range. In contrast, compounds **2a–e** after 360 nm illumination bind CXCR3 with *K*_i_ values in the low micromolar range, although the observed photoinduced affinity shifts (PAS) are not large (<4.0-fold). To assess if the compounds have agonist or antagonist activity on CXCR3-mediated signaling, a single-dose functional [^35^S]-GTPγS accumulation assay was performed with the compounds **2a–e** at 10 µM (Table 1). In this assay, we observed that most of the compounds are not activating the CXCR3 receptor in either *cis* or *trans* configuration, which indicates that the compounds bind to the receptor as antagonists. However, *cis*-**2b** and, more notably, *cis*-**2e** show a small partial agonist activity (11% and 23%, respectively) that gives a hint of a slight photoswitching of their efficacy. Both compounds have a halogen atom on the *ortho*-position of the outer ring, which seems to reaffirm the importance of the *ortho*-halogen atoms effect observed in the biaryl series [24]. However, the difference in efficacy between *cis* and *trans-*isomers (defined as PDE - photoinduced difference of efficacy) needed to be improved.

**Table 1:** Structures and results of photochemical, binding and functional characterization of compounds **2a–e** and **3a–h**.

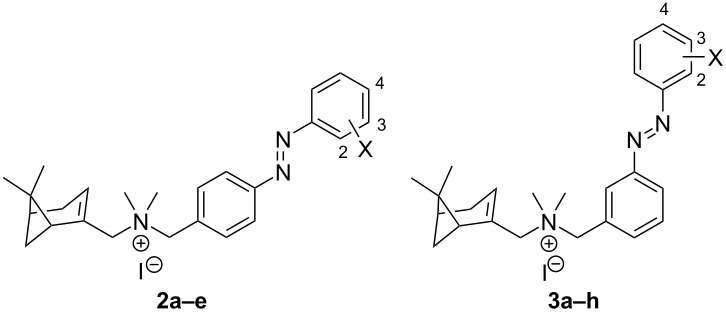															

Cmpd	*Photochemistry*					*CXCR3 binding affinity*					*Functional CXCR3 activity*				
	X	λ_max_* trans* π–π* ^a^	λ_max_* cis* n–π* ^a^	PSS_360_ (area % *cis*)^b^	SEM	p*K*_i_ *trans*^c^	SEM	p*K*_i_ PSS_360_^c^	SEM	PAS^d^	E (%) *trans*^e^	SEM	E (%) PSS_360_^f^	SEM	PDE (%)^g^

**2a**	H	321	422	88.3	0.8	6.3	0.0	5.7	0.1	4.0	−11.0	2.3	−8.3	0.7	2.7
**2b**	2-Cl	323	416	84.9	0.5	6.3	0.0	5.8	0.1	3.2	2.4	4.3	11.1	2.0	8.7
**2c**	3-Cl	320	421	79.2	0.9	6.5	0.0	6.3	0.0	1.6	−10.3	3.5	−9.6	2.7	0.7
**2d**	4-Cl	330	423	89.7	0.5	6.6	0.0	6.0	0.0	4.0	−9.8	3.5	−2.7	3.6	7.1
**2e**	2-Br	324	421	87.2	0.5	6.4	0.0	5.9	0.1	3.2	7.0	1.6	22.7	1.5	15.7

**3a**	H	320	423	82.0	1.5	6.0	0.1	5.4	0.1	4.0	−11.6	2.2	−4.7	1.7	6.9
**3b**	2-Cl	323	419	85.0	0.5	6.3	0.0	5.6	0.0	5.0	−4.7	1.7	17.4	3.7	22.1
**3c**	3-Cl	317	421	83.4	0.4	6.3	0.0	5.8	0.0	3.2	−8.4	2.2	−0.4	1.6	8.0
**3d**	4-Cl	325	424	92.0	0.3	6.4	0.0	5.7	0.0	5.0	−8.1	2.6	1.7	2.3	9.8
**3e^h^**	2-Br	323	421	88.9	0.4	6.3	0.0	5.7	0.1	4.0	−5.8	1.7	25.1	2.0	30.9
**3f**	2-I	323	422	80.9	0.2	6.2	0.0	5.8	0.1	2.5	−4.0	1.2	15.8	1.3	19.8
**3g**	3-I	318	422	82.5	1.5	6.1	0.1	6.1	0.0	1.0	−11.1	0.6	−7.6	2.3	3.5
**3h**	4-I	337	422	91.1	1.1	6.0	0.0	5.7	0.0	2.0	−15.3	1.1	5.4	1.5	20.7

^a^The absorbance maxima were extracted from UV–vis spectra at 25 µM in PBS buffer with 1% DMSO. ^b^% of *cis-*isomer at the photostationary state (PSS_360_) measured in 68% TRIS buffer and 32% DMSO (1 mM) after being pre-irradiated at 360 nm as obtained by LC–MS integration of the *cis* and *trans-*isomer signals at 265 nm. The mean and SEM of at least two experiments are shown. ^c^Binding affinity of *trans*-isomer or PSS_360_ as measured using [^3^H]-VUF11211 displacement. The mean and SEM of at least three experiments are shown. ^d^The photoinduced affinity shift (PAS) is calculated as the ratio of the *K*_i_ PSS_360_ and *K*_i_ *trans*. ^e^Normalized CXCR3 functional activity of *trans*-isomer (10 µM) in the dark (efficacy of **1d** set at 100% activity). The mean and SEM of at least three experiments are shown. ^f^Normalized CXCR3 functional activity of a sample (10 µM) pre-irradiated at 360 nm to reach the photostationary state (efficacy of **1d** set at 100% activity). The mean and SEM of at least three experiments are shown. ^g^The photoinduced difference of efficacy (PDE) is obtained by subtracting *E trans* from *E* PSS_360_. ^h^Compound was previously described by us [7].

### Optimization of positioning of azobenzene unit

Aiming to improve the position and directionality of the halogen atom, we next designed a subseries with the azo group at the *meta*-position of the central ring (scaffold **3**) instead of at the *para*-position as in **2a–e**. The analogue without halogen substitution (**3a**) as well as Cl/Br analogues comparable to the first series (**3b–e**) were prepared. Moreover, since the importance of the presence of a halogen in the outer ring was suggested in **2**, compounds **3f–h**, which include an iodine atom on the *ortho*, *meta* and *para*-positions, respectively, were synthesized.

**Scheme 2:**
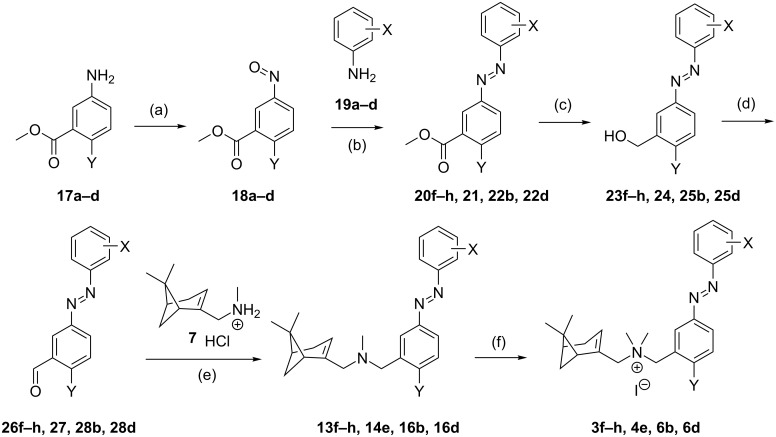
Synthetic strategies for compounds **3f–h**, **4e**, **6b**, and **6d** (Y = H, F, Cl, Br). Reagents and conditions: (a) Oxone® (2.0 equiv), DCM/H_2_O 1:4, rt, 2 h, 91–98%; (b) AcOH, 100 °C, 16–20 h, 61–96%; (c) i) DIBAL-H (3.0–4.0 equiv), THF, 0–5 °C to rt, 2–4 h; ii) NH_4_Cl (aq), Rochelle salt (10% aq), EtOAc, 1–2 h, 76–99%; or for **23h** i) DIBAL-H (1.2 equiv) , DCM, −78 °C, 1 h; ii) MeOH, −78 °C to rt, 0.5 h, iii) Rochelle salt (10% aq), 3 h, 45%; (d) Dess–Martin periodinane (1.0 equiv), DCM, rt, 1–2 h, 68–97%; (e) i) Et_3_N (1.1–1.6 equiv), DCM, rt, 10–30 min; ii) NaBH(OAc)_3_ (1.6 equiv), rt, 6–16 h, 69–96%; (f) MeI (20 equiv), DCM, rt, 6–72 h, 79–95%.

The synthesis of compounds **3a–e** was performed following the strategies shown in Scheme 1 as disclosed for compounds **2a–e**. Briefly, a reductive amination of **7** and **8b** gave nitro compound **9b**, which after reduction to **10b**, coupling with nitroso compounds **11a–e** to **13a–e** and methylation gave iodide salts **3a–e** with purities of *trans-*isomers ≥99% and overall yields similar to the ones obtained for **2a–e**. However, 2-iodonitrosobenzene cannot be accessed through oxidation of the corresponding aniline owing to oxidation sensitivity of the iodine atom. Therefore, an alternative route had to be used to synthesize **3f–h** (Scheme 2). The route began with the oxidation of methyl 3-aminobenzoate (**17a**) using Oxone^®^ to obtain a crude nitroso product **18a**, which was used in a Mills reaction with an iodoaniline (**19a–c**) at 100 °C to obtain azobenzenes **20g,h** in high yields and *ortho*-analogue **20f** in a decreased yield presumably due to steric hindrance. The methyl ester was selectively reduced with DIBAL-H to benzyl alcohols **23f–h**, which were oxidized with Dess–Martin periodinane to the corresponding benzaldehyde **26f–h**. Reductive amination of **26f–h** with **7** gave the tertiary amines **13f–h**. Methylation with iodomethane and subsequent precipitation gave **3f–h** as orange powders with ≥99% *trans*-isomer in high yields.

The photochemical properties of **3a–h** are very similar to those of **2a–e**. For the *trans-*isomers, the maximum of the π–π* band is located around 317–325 nm, with the exception of **3h** which has an iodine atom on the *para*-position and arguably confers a larger electron delocalization of π-electrons that is translated to a bathochromic shifting of the band to lower energy wavelength (337 nm). PSS values of 81–92% are obtained when illuminating with 360 nm light. The binding properties of **3a–h** also result in outcomes similar to those of **2a–e**. That is, *K*_i_ values are in the high nanomolar range for the *trans-*isomers with no or low PAS values (1.0–5.0-fold) after isomerization. In single-dose functional assays, all *trans-*isomers do not substantially activate CXCR3, whereas three of the *cis-*isomers weakly to substantially activate CXCR3 (**3b:** 17%, **3e**: 25% and **3f:** 16%). Interestingly, these three compounds are the only ones to include a halogen atom on the *ortho*-position of the outer azobenzene ring, as is the case with *para*-compounds **2b** (11%) and **2e** (23%). However, compared to the latter, *meta*-compounds **3b**,**e**,**f** display an agonist effect that is slightly higher. Increasing the size of the *ortho*-halogen does not guarantee a maximal agonist effect, as the absolute activity of **3f** is lower than that of its Br analogue **3e**. Nevertheless, evidence emerged that the *ortho*-position of the outer aromatic ring in scaffold **3** is important to achieve agonist activity of the *cis-*isomer, but it should be complemented with other strategies to further increase intrinsic activity.

### Substituent effects on the outer ring

One of the postulated contributors to the CXCR3 agonism effect of parent biaryls such as **1d** and **1e** is the increased π-electron density of the aromatic rings [24]. A way to translate this effect to the azobenzene system is by including a π-donating substituent on the aromatic system. For synthetic access and thus rapid exploration, we chose a Cl atom as mildly π-EDG (electron-donating group) even though it is σ-EWG (electron-withdrawing group) on the *para*-position of the inner ring with respect to the azo bond (i.e., the *ortho*-position with respect to the benzylic position) to afford subseries **4.** This π-electron delocalization would increase the electron density of the azobenzene unit, and was also expected to have an effect on the *trans*–*cis* azobenzene isomerization and PSS value. In terms of the outer ring, and given that *ortho* halogen atoms in subseries **2** and **3** play an important role in conferring the *cis*-isomer with partial agonism, in subseries **4** we explored halogen atoms and groups differing in, e.g., steric and electronic properties (Me, CF_3_, OMe, OCF_3_). The electron-donating groups in this series (Me and OMe) also increase the electron density of the azobenzene system.

The synthesis of **4a–d** and **4f–i** was performed following the route shown in Scheme 1. Briefly, **7** was used in a reductive amination with 2-chloro-3-nitrobenzaldehyde (**8c**) to give nitro compound **9c** which was reduced to aniline **10c** and used to obtain the azo compounds **14a–d,f–i** in variable yields through a Mills reaction with the corresponding nitroso compounds **11a-b,e–j**. Methylation of **14a–d,f–i** with MeI yielded compounds **4a–d,f–g,i** as orange powders with ≥99% *trans*-isomer in moderate to high yields. Salt **4h** did not precipitate after treatment with MTBE and was isolated as an oil, which retained substantial amounts of MTBE solvate even after extensive drying. For **4e**, we used the strategy as explained for iodo compounds **3f–h** (vide supra). Briefly, the route (Scheme 2) consists of the oxidation of methyl 5-amino-2-chlorobenzoate (**17c**) with Oxone^®^ to **18c**, which was used in a Mills reaction with 2-iodoaniline (**19a**) to yield azobenzene **21**. The methyl ester was selectively reduced with DIBAL-H and the resulting alcohol **24** oxidized with Dess–Martin periodinane to benzaldehyde **27**. Reductive amination of **27** with **7** to **14e** and subsequent methylation gave **4e** as an orange powder with ≥99% *trans*-isomer.

Photochemical characterization of **4a–i** (Table 2) gives similar results as observed for subseries **2** and **3** (Figure S2 (Supporting Information File 1) shows an exemplary time-resolved NMR and LC–MS analysis). That is, *trans* π–π* bands have a maximum between 326 and 332 nm and PSS values generally amount to over 90% of *cis*-isomer after illuminating with 360 nm light, as predicted from the electron localization provided by the chlorine atom in the central aromatic ring. Compounds **4e**, **4i** and most notably **4g** deviate from this trend, since the percentage of *cis*-isomer in the PSS is significantly lower (58–80%). *Trans*-**4a–i** show a slight decrease in binding affinity compared to the subseries **2** and **3**, amounting to low micromolar values (Table 2). Moreover, PSS affinity values are also modest, with only four compounds that have a PAS > 2.5 (**4a–c**,**f**). However, functional data from [^35^S]-GTPγS assays provide encouraging results. While all *trans*-isomers of **4a–i** do not or only weakly activate the receptor, some of the *cis-*isomers clearly behave as partial agonists. The highest efficacy is exerted by *cis*-**4c–f** with E values between 30–50% at 10 µM. Interestingly, one of these compounds (**4f**) includes a methyl group as *ortho*-substituent on the outer ring and its agonist effect at PSS amounts to 32%, possibly questioning one of our hypotheses that a halogen bond is involved in inducing CXCR3 agonism.

**Table 2:** Structure and results of photochemical, binding and functional characterization of compounds **4a–i** and **5b**,**c**.

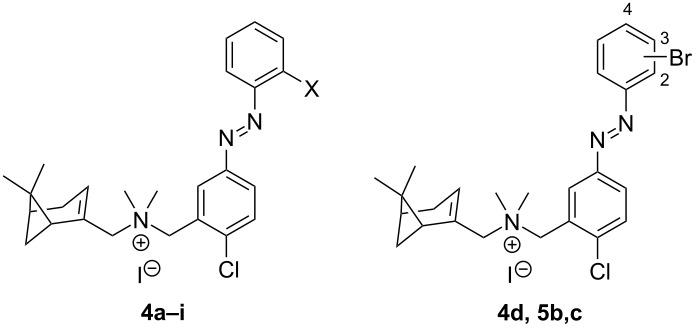															

Cmpd	*Photochemistry*					*CXCR3 binding affinity*					*Functional CXCR3 activity*				
	X	λ_max_* trans* π–π* ^a^	λ_max _* cis* n–π* ^a^	PSS_360_ (area % *cis*)^b^	SEM	p*K*_i_ *trans*^c^	SEM	p*K*_i_ PSS_360_^c^	SEM	PAS^d^	E (%) *trans*^e^	SEM	E (%) PSS_360_^f^	SEM	PDE (%)^g^

**4a**	H	326	424	91.8	1.2	5.8	0.0	5.2	0.0	4.0	−2.9	1.0	12.1	0.7	15.0
**4b**	2-F	330	420	93.0	0.2	5.6	0.0	5.0	0.0	4.0	−4.9	0.5	14.6	0.8	19.5
**4c**	2-Cl	330	419	92.8	0.1	5.8	0.0	5.3	0.1	3.2	2.3	1.6	36.9	2.0	34.6
**4d**^h^	2-Br	328	422	92.6	0.2	5.9	0.0	5.6	0.0	2.0	13.6	2.4	49.6	2.6	36.0
**4e**	2-I	330	424	79.9	1.2	5.7	0.0	5.7	0.0	1.0	16.0	1.4	37.1	0.5	21.1
**4f**	2-Me	332	428	94.9	0.1	5.9	0.0	5.3	0.0	4.0	6.9	3.1	32.2	1.1	25.3
**4g**	2-CF_3_	326	424	58.0	1.1	5.9	0.0	5.7	0.0	1.6	10.5	1.6	8.3	1.5	−2.2
**4h**	2-OMe	328	427	92.6	0.2	5.4	0.0	5.0	0.0	2.5	4.9	1.2	18.7	1.1	13.8
**4i**	2-OCF_3_	326	422	78.8	1.2	5.9	0.0	5.6	0.0	2.0	−3.0	2.0	-2.4	3.1	0.6

**4d**^h^	2-Br	328	422	92.6	0.2	5.9	0.0	5.6	0.0	2.0	13.6	2.4	49.6	2.6	36.0
**5b**	3-Br	324	420	87.4	1.2	5.7	0.1	5.6	0.0	1.3	−1.7	1.9	10.5	0.5	12.2
**5c**	4-Br	335	427	93.8	0.5	6.2	0.0	5.6	0.0	4.0	−7.2	1.8	1.8	2.5	9.0

^a^The absorbance maxima were extracted from UV–vis spectra at 25 µM in PBS buffer with 1% DMSO. ^b^% of *cis-*isomer at the photostationary state (PSS_360_) measured in 68% TRIS buffer and 32% DMSO (1 mM) after being pre-irradiated at 360 nm as obtained by LC–MS integration of the *cis* and *trans-*isomer signals at 265 nm. The mean and SEM of at least two experiments are shown. ^c^Binding affinity of *trans*-isomer or PSS_360_ in PBS (25 µM) as measured using [^3^H]-VUF11211 displacement. The mean and SEM of at least three experiments are shown. ^d^The photoinduced affinity shift (PAS) is calculated as the ratio of the *K*_i_ PSS_360_ and *K*_i_ *trans*. ^e^Normalized CXCR3 functional activity of *trans-*isomer 10 µM in the dark (efficacy of **1d** set at 100% activity). The mean and SEM of at least three experiments are shown. ^f^Normalized CXCR3 functional activity of a sample (10 µM) pre-irradiated at 360 nm to reach the photostationary state (efficacy of **1d** set at 100% activity). The mean and SEM of at least three experiments are shown. ^g^The photoinduced difference of efficacy (PDE) is obtained by subtracting E *trans* from E PSS_360_. ^h^Compound was previously described by us [7].

**Figure 3:**
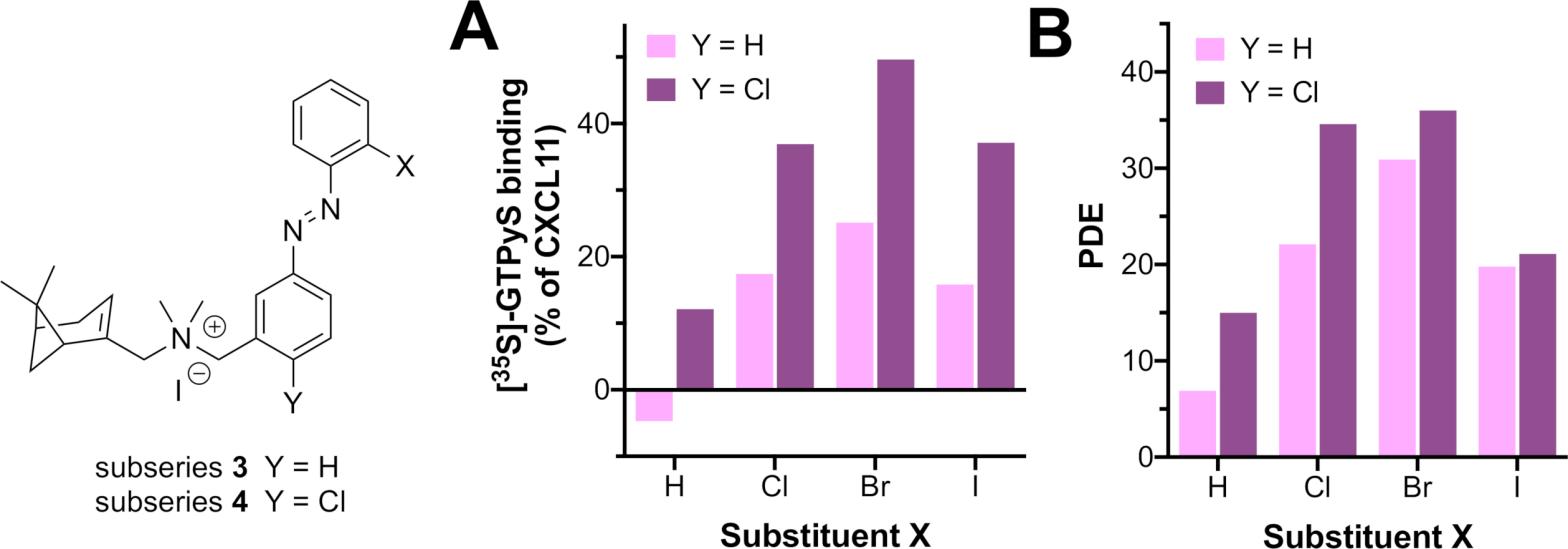
Comparison of compounds belonging to the subseries **3** or **4** with a halogen substitution on the *ortho*-position of the outer ring (X). (A) Activities of *cis*-isomers at 10 µM on CXCR3-mediated G protein activation. (B) PDE values at 10 µM.

Similar to subseries **3**, the substituent on the *ortho*-position of the outer aromatic ring appears to be a driver for the agonist activity of the corresponding *cis-*isomer. Starting from weak partial agonist **4a** (X = H), the agonist response of the *cis* increases with increasing halogen atom (**4b–d**) to a maximal response with **4d** (X = Br) having an agonist effect of 50%. However, when X = I (**4e**) the agonist activity is reduced, as also observed in the **3** subseries (compare **3d** to **3e**). In general, the agonist effects and PDE values exerted by subseries **4** are larger than those of **3** (Figure 3). This could be explained by the effect of the Cl atom present in the **4** subseries, that may give a rise in electron density to the azobenzene necessary to increase the efficacy of the *cis*-isomer.

In both subseries **3** and **4**, a bromine atom on the *ortho*-position of the outer ring gives optimal results in providing CXCR3 efficacy photoswitching (Figure 3). To confirm this for subseries **4**, the analogues of **4d** with the bromine on the *meta* and *para* position (**5b** and **5c**, respectively) were also synthesized. The synthetic route (Scheme 1) utilized **10c** and 3- and 4-bromonitrosobenzene (**11k,l**) to form azobenzenes **15b**,**c**, which were methylated to obtain **5b**,**c**. The binding affinities (Table 2) obtained for the *trans* and *cis-*isomers are all in the micromolar range. More importantly, when comparing functional results of **4d**, **5b** and **5d**, the preference for a Br atom on the *ortho*-position can also be reaffirmed for subseries **4**, because *cis-***5c** is an antagonist, while *cis*-**5b** shows only a weak activity (Table 2).

### Substituent effects on the central ring

As shown, substitution of the outer ring with an *ortho*-bromine in conjunction with a Cl substituent on the central ring appeared to pave the way for efficacy photoswitching but there was still room for improvement. Our strategy in the final optimization round was to replace the mildly π-EDG Cl atom with other groups. Thus, to aim for a full-agonist *cis* compound, the sub-series **6** was synthesized. In this subseries, different groups at the *para*-position of the central ring (Y) in combination with the *ortho*-Br atom on the outer aromatic ring are used to to explore optimal electron densities in the azobenzene system. Besides the H and Cl already explored (**3e** and **4d**, respectively), other groups including an EWG halogen atom (F, **6b**), EDG halogen atoms (Br, **6d** and I, **6e**) and stronger EDG moieties such as OMe (**6f**), OiPr (**6g**), SMe (**6h**) and NMe_2_ (**6i**) were introduced.

**Scheme 3:**
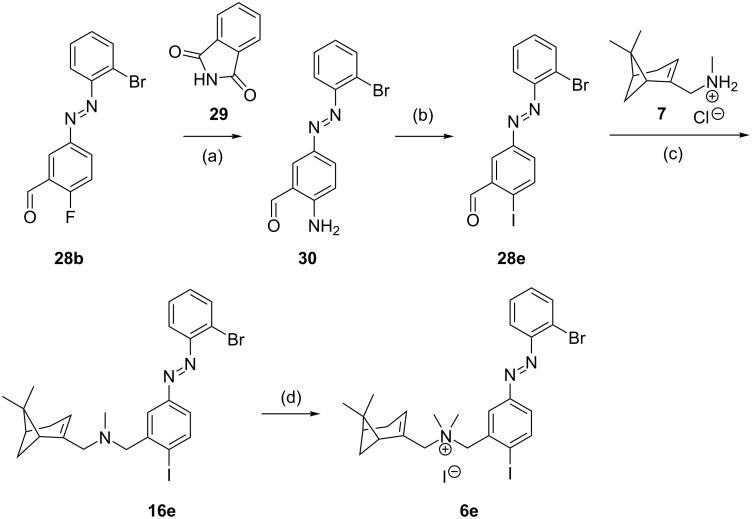
Synthetic strategy for compound **6e**. Reagents and conditions: (a) i) K_2_CO_3_ (2.0 equiv), DMF, µW, 65 °C, 3 h; ii) AcOH, reflux, 1 h, 67%; (b) i) *p*TsOH·H_2_O (3.0 equiv), MeCN, 10–15 °C; ii) NaNO_2_ (2.0 equiv), KI (2.5 equiv), H_2_O, 10–15 °C to rt, 2 h, 13%; (c) i) Et_3_N (1.2 equiv), DCM, rt, 30 min; ii) NaBH(OAc)_3_ (1.6 equiv), rt, 16 h, 54%; (f) MeI (20 equiv), DCM, rt, 20 h, 57%.

Compounds **6b** and **6d** were synthesized by the route shown in Scheme 2, using in this case halogenated methyl aminobenzoates **17b**,**c** and 2-bromoaniline **19d**. The synthetic route for compound **6e** proved more challenging. The route of Scheme 1 could not be readily used because the corresponding iodinated nitro precursor **8** is not commercially available, nor could the second route (Scheme 2) since the iodine would likely be sensitive to the first oxidation step. Therefore, we designed a new route (Scheme 3). The starting point was the advanced intermediate **28b** (Scheme 3), which was subjected to an aromatic nucleophilic substitution with potassium phthalimide prepared in situ from **29** and K_2_CO_3_. Presumably due to the alkaline medium, the phthalimide ring was partially opened as detected by HPLC–MS. Upon attempted re-closing under reflux in AcOH, completely deprotection took place and, after purification, aniline **30** was obtained with high purity. Compound **30** was used to introduce the iodine atom through a Sandmeyer reaction to give **28e** albeit in low yield. Reductive amination with **7** afforded amine **16e** followed by methylation to give *trans-***6e** as an orange solid with 97% purity.

**Scheme 4:**
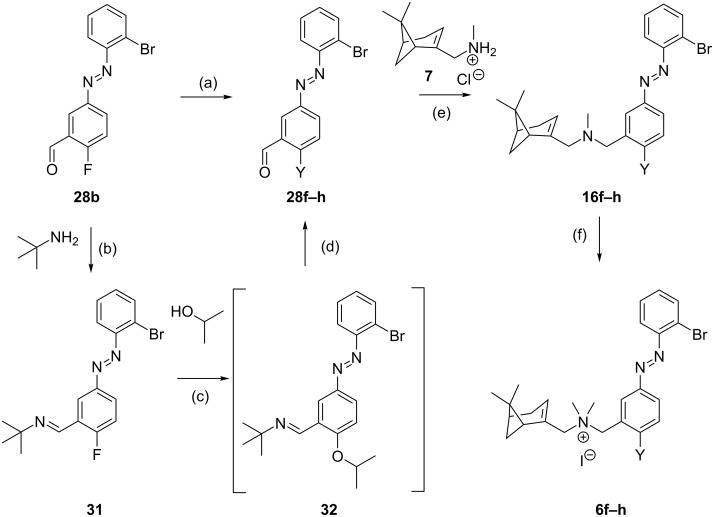
Synthetic strategies for compounds **6f–h** (Y = OMe, OiPr, SMe). Reagents and conditions: (a) NaOMe or NaSMe (1.0–1.2 equiv), MeOH or DMF, 65 °C, 30–60 min, 88–90%. (b) PhMe, 110 °C, Dean–Stark, 20 h, 99%; (c) NaH (1.0 equiv), DMSO, 100 °C, 1 h; (d) THF/H_2_O/AcOH 50:15:1, rt, 16 h, 79% (two steps); (e) i) Et_3_N (1.3–1.4 equiv), DCM, rt, 10–30 min; ii) NaBH(OAc)_3_ (1.6 equiv), rt, 6–16 h, 84–95%; (f) MeI (20 equiv), DCM, rt, 20 h, 86–90%.

The synthesis of compounds **6f–h** was performed following a nucleophilic aromatic substitution route on **28b** (Scheme 4) since precursors **8** or **17** with the required substituent Y were not available. We performed nucleophilic aromatic substitutions with the corresponding sodium salts of MeOH, 2-PrOH and MeSH under µW irradiation. Both **28f** (Y = OMe) and **28h** (Y = SMe) were formed in high yields, but the conversion of the reaction with NaOiPr was very low and partially gave reduction of the benzaldehyde. An alternative route utilized a method from Engle et al. proceeding through a *tert*-butylimine intermediate (**31**) formed under Dean–Stark conditions [32]. This imine was reacted with NaOiPr to form the ether **32**, which was subsequently hydrolyzed to obtain the desired aldehyde **28g** in high yield. Reductive amination of the aldehydes **28f–h** with **7** furnished amines **16f–h** which was followed by methylation to afford **6f–h** as orange solids with ≥98% *trans*-isomer. The synthetic strategies for the synthesis of compound **6i** were reported in our previous communication [7].

**Figure 4:**
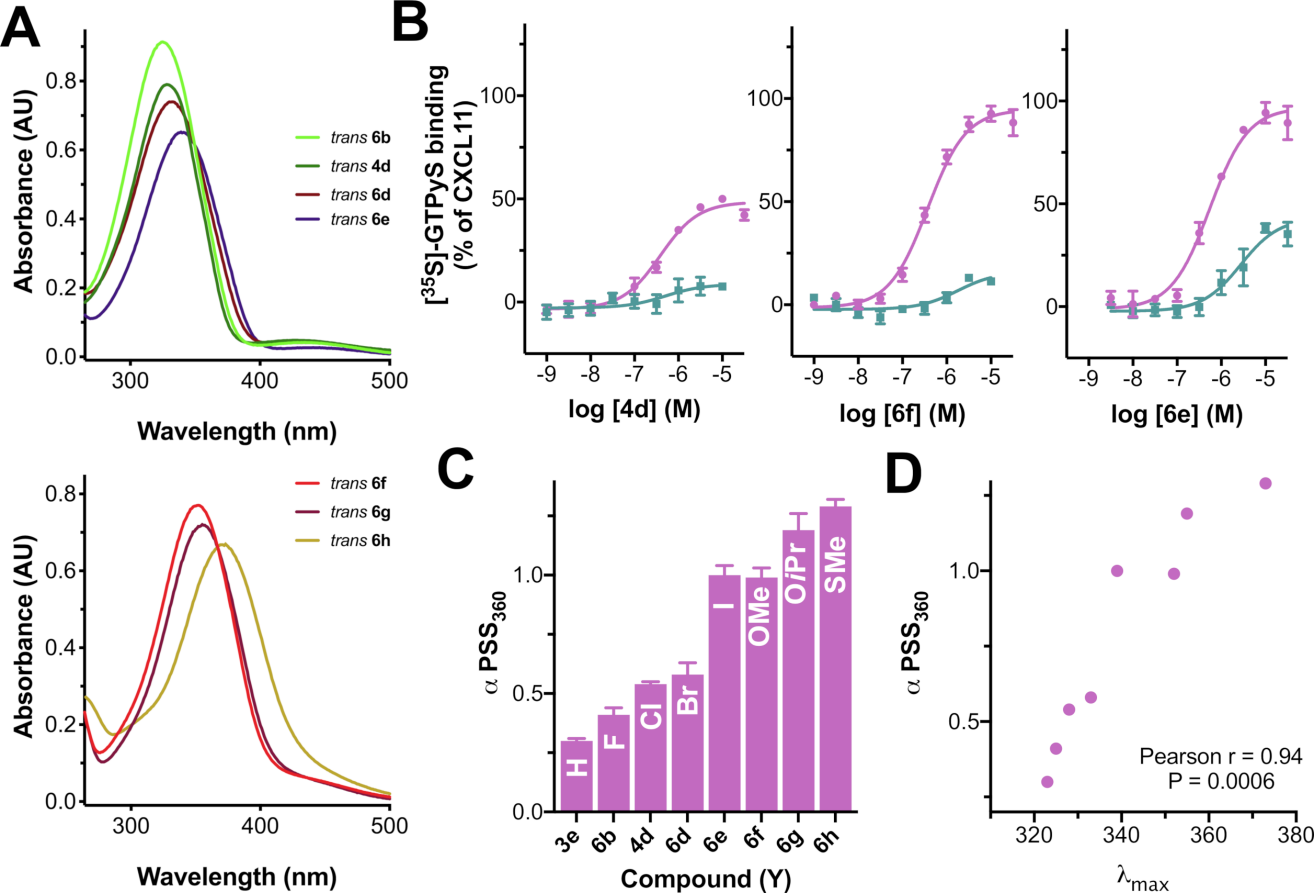
Properties of subseries **3e**, **4d**, **6b** and **6d-h**. (A) UV–vis absorption spectra of (top) *trans-*isomers of **6b**, **4d**, **6d** and **6e** (having substituent Y = F, Cl, Br and I, respectively) and (bottom) **6f**, **6g** and **6h** (having Y = OMe, OiPr and SMe, respectively). (B) Functional dose-response curves using [^35^S]-GTPγS assay exemplified for **4d**, **6f** and **6e**, respectively. (C) Summary of the efficacies of compounds **3e, 4d, 6b** and **6d–h** at PSS_360_. (D) Correlation between the bathochromic shifting of the π–π* band and the intrinsic activity of PSS_360_ for the compounds in Table 4.

In the subseries depicted in Table 3, several notable differences in both the UV–vis spectra (Figure S1, Supporting Information File 1) and the photoisomerization are observed. When the substituent Y is a halogen atom, we observe a slight bathochromic shift of the π–π* band to higher wavelengths with increasing size of the heteroatom, from 323 nm for Y = H (**3e**) to 339 nm for Y = I (**6e**) (Table 3, Figure 4A). When the group Y is OMe (**6f**), this shift is larger due to the higher EDG properties of MeO (λ_max_ = 352 nm) and this is slightly increased with Y = OiPr (**6g,** λ_max_ = 355 nm). The shift is highest for Y = NMe_2_ (**6i**, λ_max_ = 387 nm). When the oxygen atom of **6f** is replaced by a sulfur atom, the bathochromic shift of the π–π* band is also increased (**6h**, λ_max_ = 373 nm, Table 3, Figure 4A). This high capacity of sulfur substituents to induce a bathochromic effect has already been reported in the azobenzene field [33]. The *trans*–*cis* photoisomerization for **3e**, **4d**, **6b** and **6d–h** in general gives a high percentage of *cis*-isomer (89–93%) with two exceptions. Compound **6h** shows a PSS of only 65% *cis-*isomer due to a poor separation of the π–π* and n–π* bands as a result of the red-shifting by the SMe group (Figure S1A,B, Supporting Information File 1), whereas **6i** decomposes upon irradiation with 360 nm light (as previously reported by us [7]) leading to its exclusion from further characterization. The PSS_360_ forms of exemplary compounds **4d**, **6e** and **6f** have thermal half-lives of 55 [7], 28 and 29 [7] days, respectively, at 10 μM in HEPES (4-(2-hydroxyethyl)-1-piperazineethanesulfonic acid) buffer with 1% DMSO at 25 °C and we consider this to be in line with the expectations (vide supra).

**Table 3:** Structure and results of photochemical and binding characterization of compounds **3e**, **4d**, **6b** and **6d–i**.

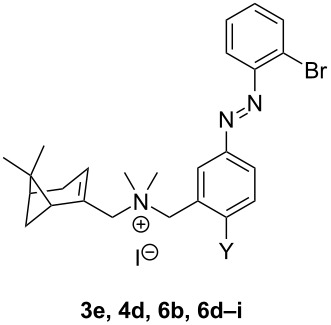										

Compound	*Photochemistry*					*CXCR3 binding affinity*				
	Y	λ_max_* trans* π–π*^a^	λ_max_* cis* n–π*^a^	PSS_360_ (% area *cis*)^b^	SEM	p*K*_i_ *trans*^c^	SEM	p*K*_i_ PSS_360_^c^	SEM	PAS^d^

**3e**^e^	H	323	421	88.9	0.4	6.3	0.0	5.7	0.1	4.0
**6b**^e^	F	325	417	92.7	0.1	6.0	0.0	5.4	0.0	4.0
**4d**^e^	Cl	328	422	92.6	0.2	5.9	0.0	5.6	0.0	2.0
**6d**^e^	Br	333	420	92.6	0.2	5.8	0.0	5.4	0.0	2.5
**6e**	I	339	424	90.5	1.0	5.5	0.1	5.5	0.0	1.0
**6f**^e^	OMe	352	424	92.1	0.1	5.4	0.0	5.0	0.0	2.5
**6g**	OiPr	355	424	89.0	0.4	5.1	0.2	5.0	0.0	1.3
**6h**	SMe	373	≈425^f^	64.5	1.7	5.3	0.0	5.5	0.1	0.6
**6i**^e^	NMe_2_	387	dec.^g^	dec.^g^						

^a^The absorbance maxima were extracted from UV–vis spectra at 25 µM in PBS buffer with 1% DMSO. ^b^% of *cis-*isomer at the photostationary state (PSS_360_) in 68% TRIS buffer and 32% DMSO (1 mM) measured after being pre-irradiated at 360 nm as obtained by LC–MS integration of the *cis* and *trans*-isomer signals at 265 nm. The mean and SEM of at least two experiments are shown. ^c^Binding affinity of *trans*-isomer or PSS_360_ as measured using [^3^H]-VUF11211 displacement. The mean and SEM of at least three experiments are shown. ^d^The photoinduced affinity shift (PAS) is calculated as the ratio of the *K*_i_ PSS_360_ and *K*_i_ *trans*. ^e^Compound was previously described by us [7]. ^f^Could not be determined accurately due to partial overlapping of the π–π* and n–π* bands. ^g^Compound decomposes under illumination.

The binding affinity of *trans*-**3e, 4d, 6b** and **6d–h** (Table 3) is in the low micromolar range with a low PAS value upon illumination. Initial pilot studies on [^35^S]-GTPγS binding after CXCR3 stimulation with single concentrations of subseries **6** (data not shown) showed substantial levels of CXCR3 agonism in this group of compounds. For subseries **6** (and associated **3e** and **4d**) we therefore generated dose-response curves for the *trans* and PSS_360_ forms (Figure S4, Supporting Information File 1) using the same [^35^S]-GTPγS functional assay and calculated the intrinsic activity (α) and potency (EC_50_). As reported in our previous communication for some of these compounds [7], the PSS_360_ forms give agonism with high nanomolar potencies while most *trans* compounds are antagonists or partial agonists with very low efficacies (Table 4). However, when the size and/or EDG properties of Y increase, remarkably partial agonism with substantial efficacies appears even for the *trans*-isomers, such as for **6e**, **6g** and **6h**. The compounds illuminated to PSS_360_ follow a similar qualitative trend: when the Y substituent is H or F (**3e** and **6b**), the *cis*-isomer behaves as a partial agonist with medium efficacy (α = 0.30–0.41), but when the Y substituent increases in size and/or EDG properties, the efficacy increases to full efficacy with compounds **6e–h** (Y = I, OMe, OiPr and SMe). The *trans-*form of **6g** shows anomalous behavior at 10^−5^ M and higher (Figure S4, Supporting Information File 1), preventing the extraction of accurate functional values. The PDE value, which reflects efficacies of both the *trans* and PSS form, appears to have an optimum for **6f** (Figure 4B). Figure 4B also shows more subtle behavior for compounds **4d** and **6e**, which harbor a PDE value of 0.43 and 0.51, respectively, but in different parts of the efficacy window.

**Table 4:** Structure and results of functional characterization of compounds **3e**, **4d**, **6b** and **6d–h**.

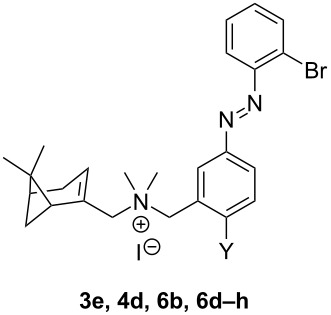										

Compound	Y	pEC_50_ *trans*^a^	SEM	pEC_50_ PSS_360_^b^	SEM	α *trans*^c^	SEM	α PSS_360_^d^	SEM	PDE^e^

**1d**	–	6.9	0.0	6.9	0.0	1.04	0.08	1.03	0.07	−0.01
**3e**^f^	H	n.m.^g^	n.m.^g^	6.5	0.1	0.05	0.03	0.30	0.01	0.25
**6b**^f^	F	n.m.^g^	n.m.^g^	6.2	0.1	0.12	0.00	0.41	0.03	0.29
**4d**^f^	Cl	n.m.^g^	n.m.^g^	6.3	0.1	0.11	0.01	0.54	0.01	0.43
**6d**^f^	Br	n.m.^g^	n.m.^g^	6.2	0.1	0.14	0.02	0.58	0.05	0.44
**6e**	I	5.5	0.3	6.3	0.2	0.49	0.04	1.00	0.04	0.51
**6f**^f^	OMe	n.m.^g^	n.m.^g^	6.4	0.1	0.16	0.01	0.99	0.04	0.83
**6g**	OiPr	n.m.^h^	n.m.^h^	6.0	0.0	n.m.^h^	n.m.^h^	1.19	0.07	n.m.^h^
**6h**	SMe	5.7	0.3	6.1	0.1	0.85	0.08	1.29	0.03	0.44

^a^Potency of *trans-*isomer in the dark. n.m. = not measurable. The mean and SEM of at least three experiments are shown. ^b^Potency of a sample pre-irradiated at 360 nm to reach the photostationary state. The mean and SEM of at least three experiments are shown. ^c^Intrinsic activity of *trans-*isomer in the dark (CXCL11 efficacy set at α = 1). The mean and SEM of at least three experiments are shown. ^d^Intrinsic activity of a sample pre-irradiated at 360 nm to reach the photostationary state (CXCL11 efficacy set at α = 1). The mean and SEM of at least three experiments are shown. ^e^The photoinduced difference of efficacy (PDE) is obtained by subtracting α *trans* from α PSS_360_. ^f^Compound was previously described by us [7]. ^g^Too low window. ^h^The curve for *trans*-**6g** shows anomalous behavior at 10^−5^ M and higher.

The results reveal that the electron density of the aryl rings, especially of the inner one, plays a key role in inducing agonism in CXCR3. In general, the *cis-*isomers of the series **2–6** are better CXCR3 agonists. Indeed, the *cis*-isomers are generally assumed to have an intrinsically higher electron density due to the disruption of the conjugation between the two rings of the azobenzene through the N=N bond. Moreover, the electronic properties of the inner substituent Y have been proven to be of strategic use in increasing the intrinsic activity (α) of the *cis*-isomers (Figure 4C). This capacity of the Y substituent to alter the electron density is conceivably also related to its capacity to induce a bathochromic shift of the π–π* band of the *trans-*isomer. Potential evidence for this can be extracted from the significant correlation between the bathochromic shift and intrinsic activity of PSS_360_ (Figure 4D) for the subseries **3e, 4d, 6b** and **6d–h**, which only differ in the nature of Y group (Figure 4C, Table 4).

## Conclusion

We report a toolbox of 31 photochromic small-molecule CXCR3 receptor ligands based on the modeling-assisted azologization of a biaryl series reported previously by us [24]. All compounds show affinity for CXCR3 from the high nanomolar to the low micromolar range. Our efforts, however, were focused on exploring the landscape in functional efficacy. To this end, the scaffold was subjected to positional and substitution changes in structure, necessitating extensive synthetic efforts through multiple routes. The presence of halogen substituents on the *ortho*-position of the outer ring (substituent X) provides partial agonism for the *cis-*isomer with a Br atom being the major exponent, while *trans-*isomers preserve antagonist behavior. The presence of a substituent on the *para-*position of the central ring (substituent Y) capable of delocalizing π-electrons increases the efficacy of the *cis-*isomer. The *cis-*isomers of compounds with Y = I, OMe, OiPr or SMe are all full agonists of CXCR3, however, the corresponding *trans-*isomers also activate the receptor to varying degrees. In all, our efforts deliver a spectrum of (subtle) efficacy differences. Notable tool compounds are VUF15888 (**4d**) switching from antagonism to partial agonism (PDE = 0.43), VUF16620 (**6e**) switching from partial agonism to full agonism (PDE = 0.51), and VUF16216 (**6f**), which represents the optimum balance and provides a CXCR3 photoswitch with a PDE value of 0.83, i.e., from antagonism to full agonism. Based on the pharmacological properties of these three compounds and the long half-lives of their PSS states, they will be valuable tools for future photopharmacological studies on the dynamic signaling of the chemokine receptor CXCR3.

## Supporting Information

File 1 Experimental part.
