# Supplementary material for: A toolbox of molecular photoswitches to modulate the CXCR3 chemokine receptor with light
Source: Beilstein J Org Chem. 2019 Oct 23;15:2509–23. doi: 10.3762/bjoc.15.244 (PMC6839561; doi:10.3762/bjoc.15.244)
Supplement: File 1 — Experimental part. [file Beilstein_J_Org_Chem-15-2509-s001.pdf]

BEILSTEIN JOURNAL OF ORGANIC CHEMISTRY

Supporting Information

for

A toolbox of molecular photoswitches to modulate the CXCR3 chemokine receptor with light

Xavier Gómez-Santacana, Sabrina M. de Munnik, Tamara A. M. Mocking, Niels J. Hauwert, Shanliang Sun, Prashanna Vijayachandran, Iwan J. P. de Esch, Henry F. Vischer, Maikel Wijtmans and Rob Leurs

Beilstein J. Org. Chem. **2019,** *15,* 2509–2523. doi:10.3762/bjoc.15.244

Experimental part

License and Terms: This is a supporting information file under the terms of the Creative Commons Attribution License (http://creativecommons.org/licenses/by/4.0). Please note that the reuse, redistribution and reproduction in particular requires that the authors and source are credited.
The license is subject to the *Beilstein Journal of Organic Chemistry* terms and conditions: (https://www.beilstein-journals.org/bjoc)

Table of contents

Page	Item
S2	Computational methods
S3	Photochemistry procedures
S4–S5	Figure S1. UV spectra
S6	Figure S2. Time-resolved analysis of irradiation by NMR and LC.
S7	Figure S3. Arrhenius plot
S8–S9	Pharmacology procedures
S10	Figure S4: Full functional dose-response curves
S11–S47	Synthesis methods and procedures
S48–S50	NMR Spectra
S51–S54	LC-PDA-MS Chromatograms
S55	References

Computational methods

The small molecules were built and optimized using MOE (Molecular Operating Environment, version 2016.0802). A conformational analysis using the stochastic search method (default settings) was performed to obtain the ideal conformation of the azobenzene moieties (taken from the global energy minimum conformation). The potential of the azobenzene atoms was fixed to ensure an optimal conformation throughout molecular superpositions. The three-dimensional structures of **1e** and the azobenzene analogues (*trans*- and *cis*-**2a**) were superposed using the flexible alignment module, using volume as the similarity term (weight 3). Other settings were used as default. For the superposition of *trans*-**2a** with **1e**, the alignment with the lowest alignment score S of −75.5234 was selected, which is the sum of the average strain energy U (95.8843 kcal/mol) and the similarity score F (−171.4077) values. The same procedure was used with **1e** and *cis*-**2a**, leading to the best alignment with S, U and F values of −197.7601, 95.3657 kcal/mol and −293.1258, respectively.

Photochemistry procedures

The photochemistry procedures are the same or similar to those published previously by us[1].

UV–vis spectra were obtained using a Thermo-scientific Evolution 201 PC spectrophotometer. Fits of UV–vis spectroscopy data were generated using GraphPad Prism 6 or 7 software. Illumination was executed using a Sutter instruments Lambda LS with a 300 Watt full-spectrum lamp connected to a Sutter instruments Lambda 10-3 optical filter changer equipped with 494 ± 9 nm, 460 ± 5 nm, 434 ± 9 nm and 360 ± 20 nm filters. The light intensity is 0.77 mW/mm^2 for the 360 ± 20 nm filter, 0.57 mW/mm^2 for the 434 ± 9 nm filter, 0.26 mW/mm^2 for the 460 ± 5 nm filter and 0.77 mW/mm^2 for the 494 ± 9 nm filter as measured using a Thorlabs PM16-401 power meter. For the determination of UV–vis spectra, the *cis*-to-*trans* thermal relaxation or the *cis*-to-*trans* back isomerisation, illuminations were performed in Hellma Suprasil™ quartz 114-QS cuvettes with a 360 nm, 434 nm, 460 nm or 494 nm filter during 2 min. Samples were 25 µM in PBS buffer + 1% DMSO.

Selected *cis*-to-*trans* thermal relaxations were performed in the dark at 10 µM PSS_{360} in HEPES solution with 1% DMSO, either by prolonged measuring at 25 °C (**4d**, **6f**) [1] or by an Arrhenius extrapolation method from measurements at higher temperatures (**6e**, Figure S3) [2].

Illuminations for pharmacological experiments were performed in cylindrical clear glass vials with a volume of 150 µL during 30 min with a 360-nm filter. Samples were 1.0 mM in a mixture of 68 vol % TRIS binding buffer and 32 vol % DMSO, or 1.0 mM in a mixture of 68 vol % HEPES binding buffer and 32 vol % DMSO (for $[^{35}S]$-GTPγS binding experiments). For compounds **6e**,**g**,**h**, irradiation was performed at 3.16 mM in 100 % DMSO and subsequent dilution adapted accordingly. The photoisomerization and photostability was monitored by LC-PDA for all the samples. The typical distance between light source and vial or cuvette was 2 cm.

A

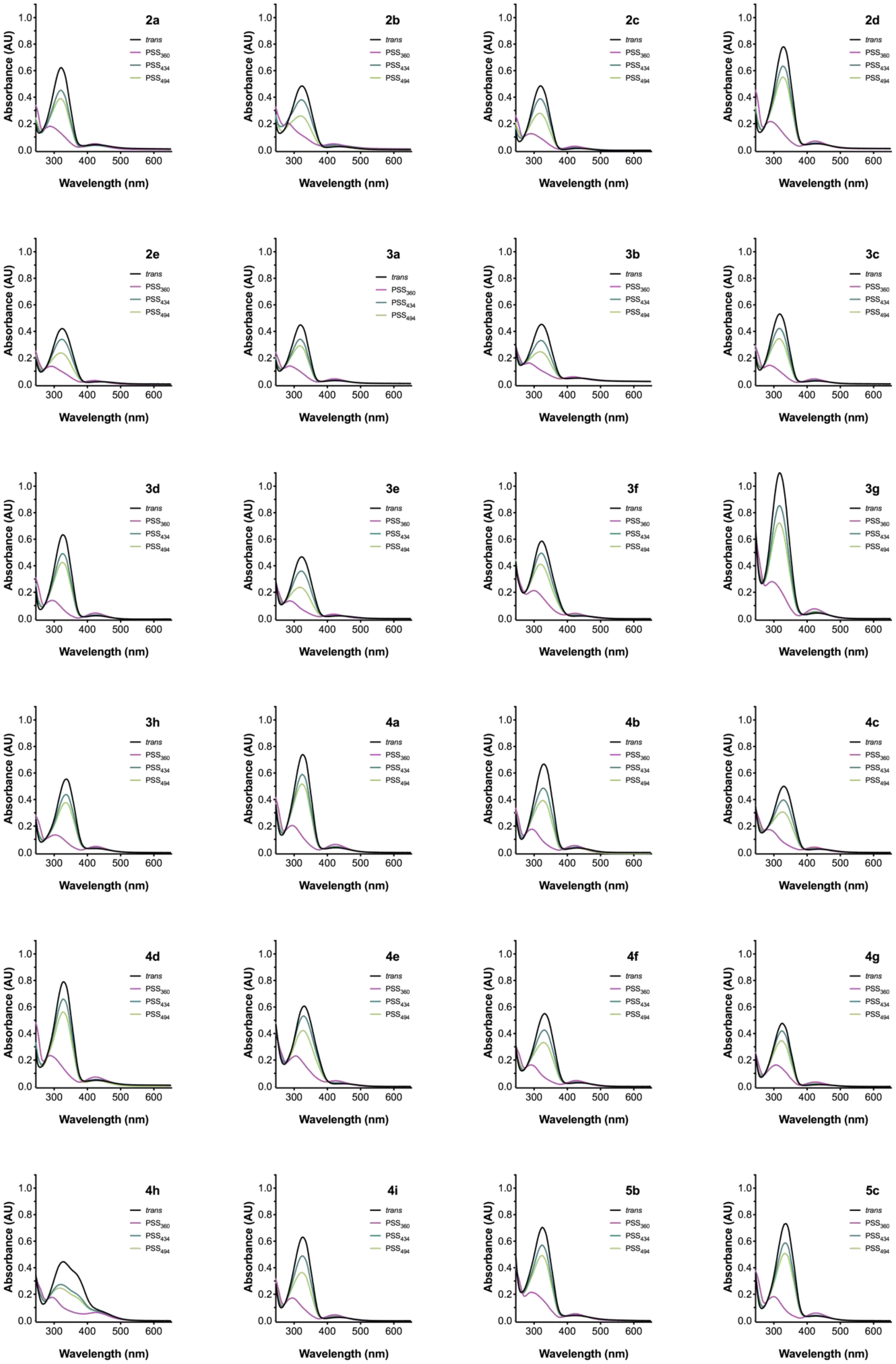
2a
2b
2c
2d
2e
3a
3b
3c
3d
3e
3f
3g
3h
4a
4b
4c
4d
4e
4f
4g
4h
4i
5b
5c
trans
PSS360
PSS434
PSS494
Absorbance (AU)
Wavelength (nm)

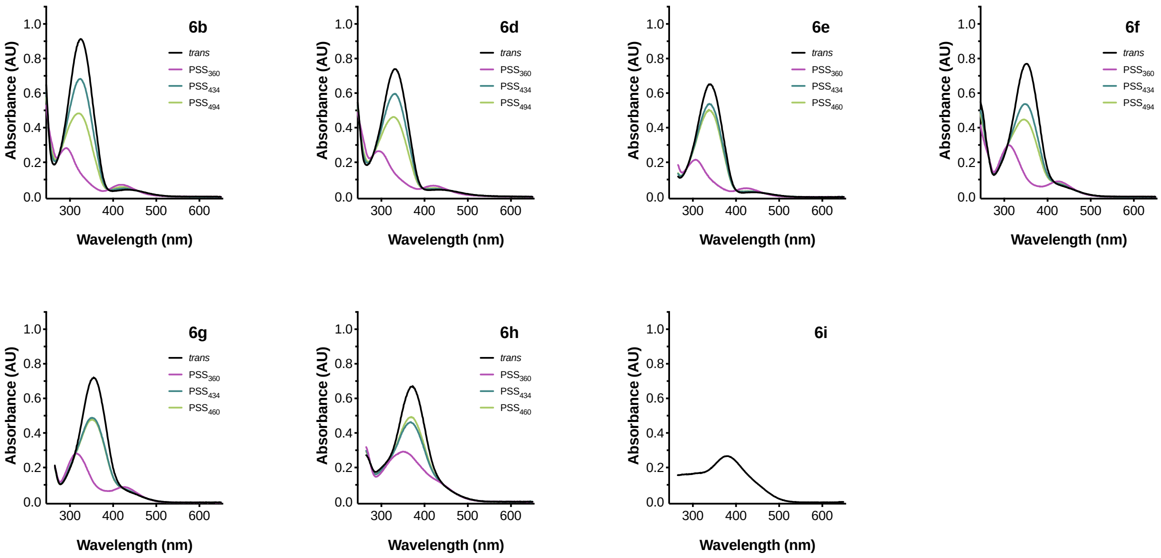

B

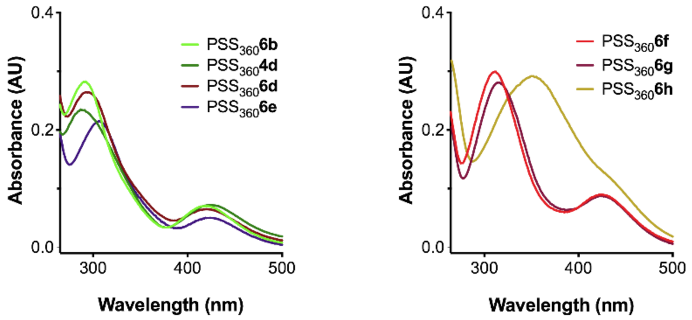

Figure S1. (A) UV–vis absorption spectra of compounds as *trans*- or as PSS-forms after irradiation with various different wavelengths. (B) Overlay of UV–vis absorption spectra of PSS_{360} forms of **6b**, **4d**, **6d** and **6e** (having substituent Y = F, Cl, Br and I respectively) and **6f**, **6g** and **6h** (having Y = OMe, OiPr and SMe, respectively).

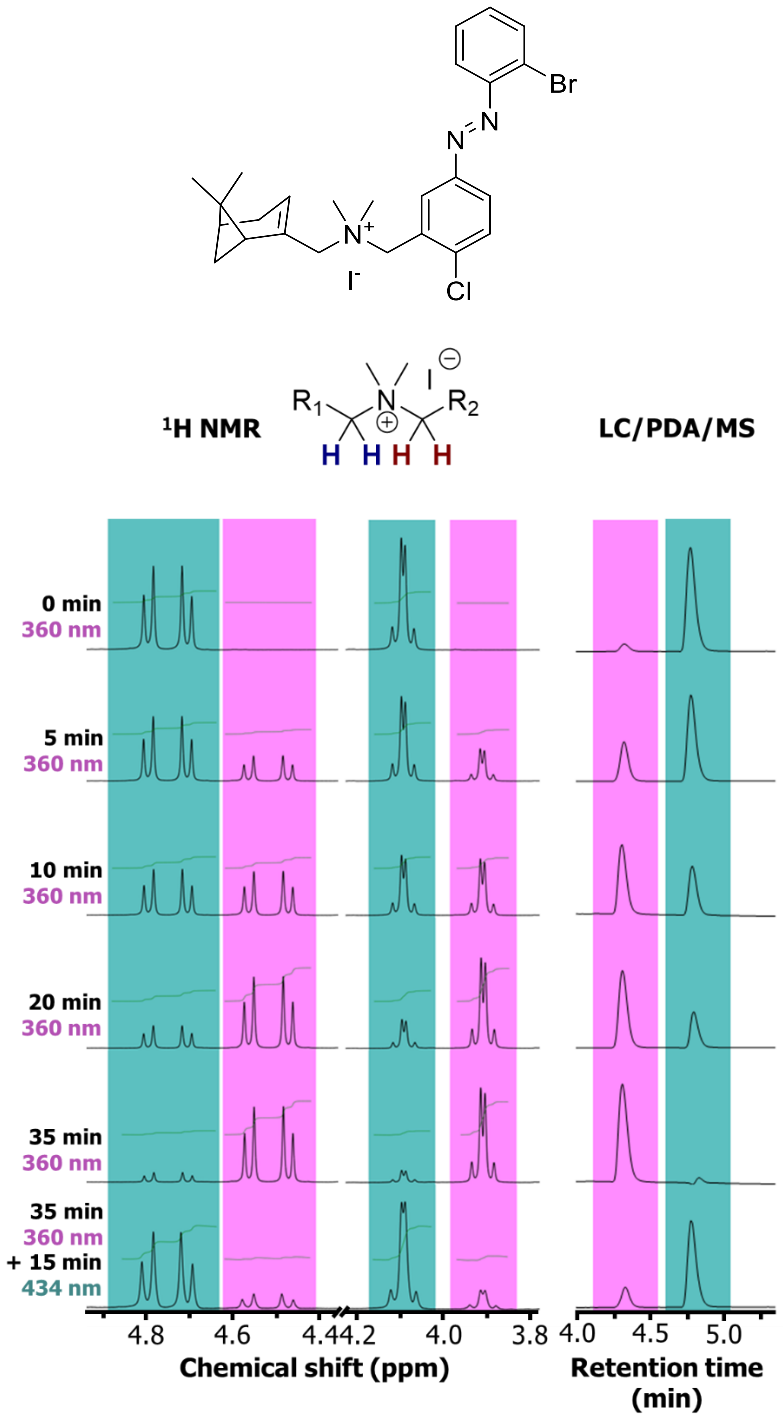

Figure S2. Monitoring of photoisomerisation over time of **4d** (10 mM in DMSO-d_6) from *trans* to *PSS cis* (λ = 360 nm) and from *PSS cis* to *PSS trans* (λ = 434 nm) as followed by ^{1}H NMR spectroscopy (integration of α-ammonium-methylene protons) and LC-PDA-MS analysis (integration at 276 nm).

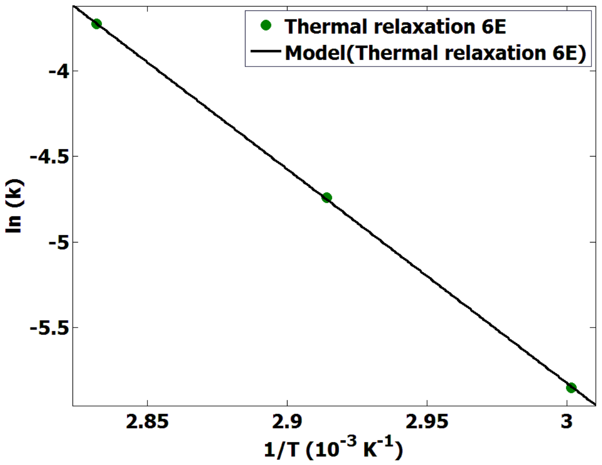

Figure S3. Arrhenius plot for **6e** measured at a concentration of 10 μM in HEPES buffer with 1% DMSO-d_6. The value provided in the main text is an extrapolation of the linear fit presented in this plot. The R^2 value for linear fit was over 0.999.

Pharmacology procedures

The pharmacology procedures are the same or similar to those published previously by us [1].

Cell culture and transfection: Human embryonic kidney 293T (HEK293T) cells were cultured at 37 °C and 5% CO_2 in Dulbecco's modified Eagle's medium (Thermo Scientific, Waltham, MA, USA) supplemented with 10% fetal bovine serum (Bodinco, Alkmaar, The Netherlands), 50 IU/mL penicillin and 50 µg/mL streptomycin (Thermo Scientific). Cells were transfected using linear polyethylenimine (PEI) with a molecular weight of 25 kDa (Polysciences, Warrington, PA, USA). Briefly, 1 µg cDNA encoding human CXCR3 (isoform A) and 4 µg empty vector pcDEF$_3$ (a gift from Dr. Langer, Robert Wood Johnson Medical School, Piscataway, NJ, USA) was diluted in a total volume of 250 µL NaCl solution (150 mM). Next, 30 µg PEI diluted in a total volume of 250 µL NaCl solution was added to the DNA solution, mixed and incubated for 20 min at 22 °C. The DNA/PEI mixture was subsequently added to the medium of adherent HEK293T cells.

Membrane preparation: Membranes were prepared as previously described [3]. In brief, 48 h post-transfection, CXCR3-expressing HEK293T cells were collected in phosphate buffered saline (PBS). Then, cells were subsequently centrifuged at 1500xg for 10 min at 4 °C and washed with PBS. The cell pellet was resuspended in ice-cold membrane buffer (15 mM Tris, pH 7.5, 1 mM EGTA, 0.3 mM EDTA and 2 mM $MgCl_2$) and homogenized using a Teflon-glass potter. Next, the membranes were frozen in liquid N_2 and thawed twice and subsequently centrifuged at 40.000xg for 25 min at 4°C. The pellet was resuspended in Tris-sucrose buffer (20 mM Tris, pH 7.4, 250 mM sucrose) and further homogenized through a 23G needle. Aliquots were quickly frozen in liquid nitrogen and stored at −80 °C.

[^{3}H]-VUF11211 binding assay: [^{3}H]-VUF11211 binding was performed as previously described [3]. In brief, CXCR3 membranes were incubated in black 96-well plates with increasing concentrations photoswitchable ligand and 1 nM [^{3}H]VUF11211 (PerkinElmer Life Sciences, Boston, MA, USA, 38.4 Ci/mmol) in binding buffer (50 mM Tris-HCl, pH 7.4, 100 mM NaCl, 0.1% (w/v) Tween80, 0.1% (w/v) BSA fraction V) for 2 h at 25 °C (shaking at 600 rpm and protected from light). After incubation, the membranes were harvested by rapid filtration and washing with ice-cold wash buffer (50 mM Tris-HCl, pH 7.4, 0.5 M NaCl) over 96-well GF/C filter plates (PerkinElmer) that were presoaked in 0.5% w/v BSA/H_2O. The GF/C plates were dried and scintillation liquid was added to determine bound radioactivity with a MicroBeta scintillation counter (PerkinElmer).

[^{35}S]-GTPγS binding assay: [^{35}S]-GTPγS binding was performed as previously described [4]. Briefly, 5 µg/well CXCR3-membranes were incubated in a black 96-well plate with indicated concentrations of photoswitchable ligand and 300 pM [^{35}S]GTP NPD-3079S (PerkinElmer, 1250 Ci/mmol) in assay buffer (50 mM HEPES, 10 mM $MgCl_2$, and 100 mM NaCl, pH 7.2), supplemented with 3 µM GDP (Sigma-Aldrich) and 5 µg/well saponin for 1 h at 25 °C (shaking at 600 rpm and protected from light). Next, the CXCR3 membranes were harvested by rapid filtration through Unifilter GF/B plates (PerkinElmer) using ice-cold washing buffer (50 mM Tris-HCl, pH 7.4, 5 mM $MgCl_2$) and scintillation liquid was added after drying the GF/B plates. [^{35}S]GTPγS membrane binding was determined using a Microbeta scintillation counter (PerkinElmer) and the response was normalized to that of the endogenous agonist CXCL11 (PeproTech, Rocky Hill, NJ, USA) to determine the relative efficacy of the photoswitchable ligands (α value).

Data analysis: GraphPad Prism 6, 7 or 8 software (GraphPad Software, San Diego) was used for plotting radioligand displacement curves and sigmoidal concentration-response curves and to

determine IC_{50} and pEC_{50} values, respectively, by nonlinear regression. The IC_{50} values were converted to K_i values using the method of Cheng and Prusoff [5]. Statistical analyses were performed using Graphpad Prism 6, 7 or 8 software.

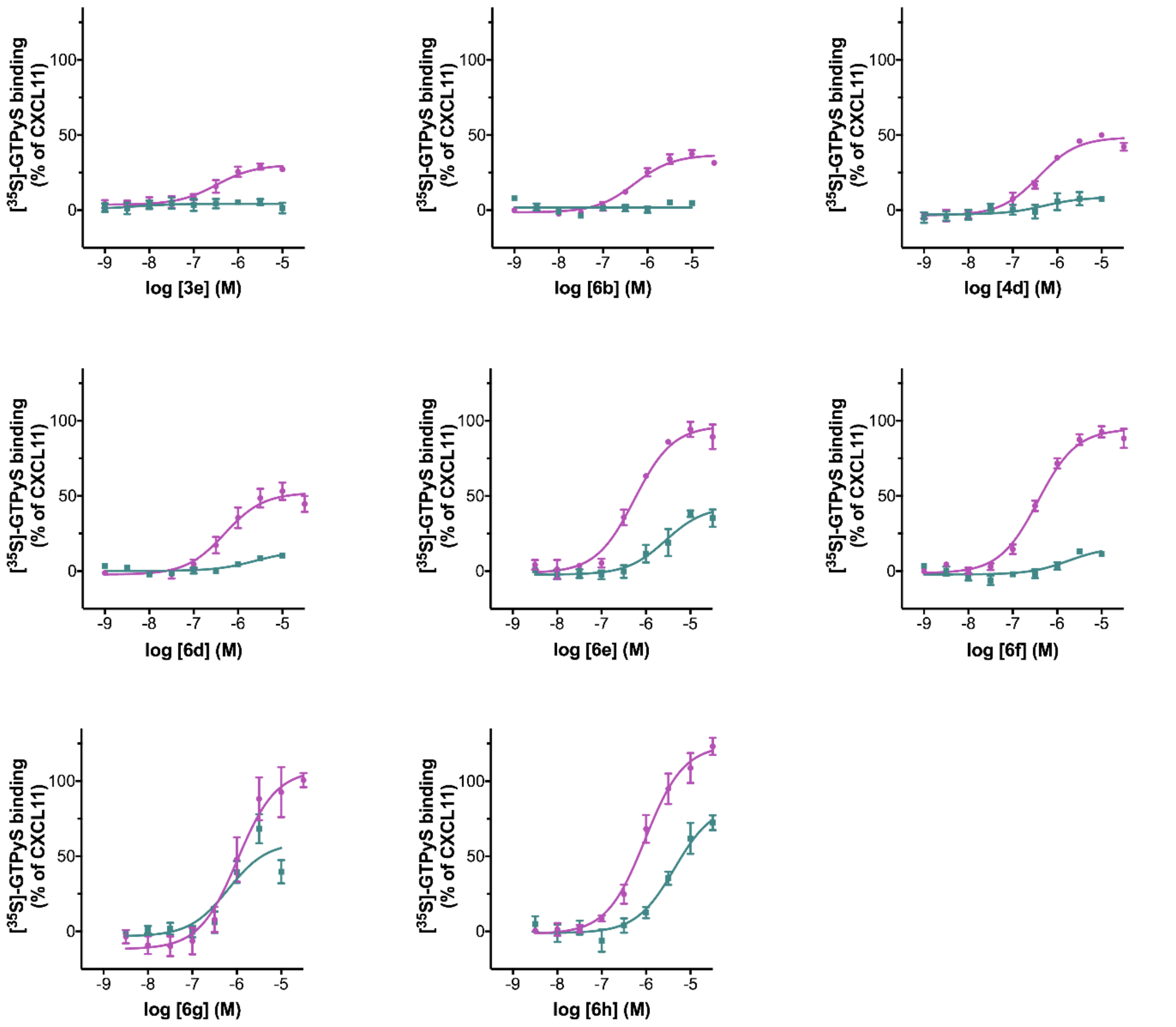

Figure S4. Full functional dose-response curves for compounds **3e, 4d, 6b** and **6d**-**h** in *trans* (turquoise) or PSS_{360} (magenta) form. Dose-response curves for compounds **3e, 4d, 6b**, **6d** and **6f** have been published by us before [1].

Synthesis methods and procedures

Unless mentioned otherwise, all reactions were performed under N_2 atmosphere. All chemicals and solvents were obtained from commercial suppliers (primarily Sigma-Aldrich, Acros Organics, Fluorochem and Combi-Blocks) and used without purification. DCM, DMF, THF and Et_2O were dried by passing through a PureSolv solvent purification system. Triethylamine (TEA) was dried over alumina. Compounds **2–6** were synthesized according the procedures detailed below. Compound **6i** and **7** were synthesized as reported previously [1,6]. Reactions were monitored by thin layer chromatography (Merck Silicagel 60 F254) by visualization under 254 nm lamp or under natural light conditions (for colored compounds). Flash column chromatography was performed with SNAP KP-Sil 50 µm (Biotage) or GraceResolv (Büchi) cartridges on Isolera One with UV–vis detection (Biotage). Nuclear magnetic resonance (NMR) spectra were determined with a Brücker Avance 500 Ultrashield or a Brücker Avance 600 Ultrashield plus spectrometer. Chemical shifts are reported in parts per million (ppm) against the reference compound using the signal of the residual non-deuterated solvent ($CDCl_3$ δ = 7.26 ppm (^{1}H), δ = 77.16 ppm (^{13}C). NMR spectra were processed using MestreNova 10.0.2 software. The peak multiplicities are defined as follows: s, singlet; d, doublet; t, triplet; q, quartet; dd, doublet of doublets; ddd, doublet of doublets of doublets; dt, doublet of triplets; dq, doublet of quartets; td, triplet of doublets; tt, triplet of triplets; br, broad signal; m, multiplet; app, apparent. Purity determination was performed with Liquid Chromatography using a Shimadzu LC-20AD liquid chromatography pump system with a Shimadzu SPDM20A photodiode array detector and MS detection with a Shimadzu LCMS-2010EV mass spectrometer operating in both positive and negative ionization mode. A Waters XBridge C18 column 5 µm 4.6 × 50 mm was used at 40 °C. The mobile phase used was a mixture of A = water + 0.1% HCO_2H and B = acetonitrile (MeCN) + 0.1% HCO_2H. The eluent program used is as follows: flow rate: 1.0 mL/min, start 95% A in a linear gradient to 10% A over 4.5 min, hold 1.5 min at 10% A, in 0.5 min in a linear gradient to 95% A, hold 1.5 min at 95% A, total runtime: 8.0 min. Compound purities were calculated as the percentage peak area of the analyzed compound by UV detection at 254 nm. All chemistry and analyses of photosensitive compounds were carried out under dimmed or red light. High-resolution mass spectra (HRMS) were recorded on a Bruker micrOTOF mass spectrometer using ESI in positive ion mode (HRMS).

General synthetic procedures

Many general synthetic procedures are the same or similar to those published previously by us [1].

*General synthetic procedure A for compounds **9a–c, 13f–h, 14e** and **16b,d–h**:* In a round-bottom flask, compound **7** (1.0–1.3 equiv) and **8a–c**, **26f–h**, **27** or **28b,d–h** (1.0 equiv) were dissolved in DCE. TEA (1.1–1.6 equiv) was added. The solution was stirred at rt for 30 min. Subsequently, $NaBH(OAc)_3$ (1.6 equiv) was added and the solution was stirred from 6 to 16 h at rt. After that, aq. Na_2CO_3 (2 M) was added and the resulting mixture was stirred for 10 min. DCM was added. The layers were separated and the aqueous layer was extracted twice with DCM. The combined organic layers were dried over anhydrous Na_2SO_4, filtered and concentrated in vacuo to give a residue, which was purified with automated flash chromatography (cHex/EtOAc + 1% TEA) to give compounds **9a–c**, **13f–h**, **14e** and **16b,d–h** as a colourless/orange oil with high purity.

*General synthetic procedure B for compounds **10a–c**:* In a round bottom flask, a mixture of compound **9a–c** (1.0 equiv) and $SnCl_2{\cdot}2H_2O$ (5 equiv) in EtOH was stirred for 2 h at 75 °C. Subsequently, the mixture was filtered through Celite and washed with DCM. The resulting solution was washed twice with aq. Na_2CO_3 (2 M), dried over anhydrous Na_2SO_4, filtered and concentrated in vacuo to give

compounds **10a–c** as yellow oils, which were used without further purification.

General synthetic procedure C for compounds ***12a–e, 13a–e, 14a*–d**, **14f–i** *and* ***15b,c:*** A solution of Oxone™ (3.0 equiv) in water (4 vol) was added to a solution of the corresponding aniline (1.5 equiv) in DCM (1 vol). The resulting mixture was stirred vigorously at rt during 2 h. Water was added and the layers were separated. The green organic layer was washed with aq. HCl (1 M), water and brine. The resulting green nitrosobenzene solution was used directly by mixing with the corresponding aniline (1.0 equiv). For the preparation of compound **12a** and **13a**, the commercially available nitrosobenzene (**11a**) was directly used (1.5 equiv) dissolved in DCM. Two drops of AcOH were added and the resulting solution was left stirring for 1–5 d protected from light. Subsequently, the solvents were removed in vacuo and the resulting dark residue was purified with automated flash chromatography (cHex/DCM/EtOAc + 1% TEA) to give compounds **12a–e**, **13a–e, 14a–d**, **14f–i** and **15b,c** as an orange/red oil with high purity.

General synthetic procedure D for compounds ***2a–e, 3a–h, 4a–6, 5b,c, 6b*** *and* ***6d–i:*** In a round-bottom flask, the tertiary amine precursor **12a–e**, **13a–h**, **14a–6**, **15b,c**, **16b** and **16d–i** (1.0 equiv) was dissolved in DCM. The flask was closed with a septum and protected from light. Excess MeI (20 equiv) was added to the solution via a syringe. The reaction mixture was stirred at rt in the dark during 6–72 h. The reaction mixture was cooled in an ice bath, and MTBE (3 vol equiv with respect to DCM) was added slowly. This induced precipitation of the salt, which was filtered and washed with a precooled solution of DCM/MTBE (1:3). This delivered the product **2a–e**, **3a–h**, **4a–6**, **5b,c**, **6b** and **6d–i** as an orange solid with high purity.

General synthetic procedure E for compounds ***18a–d:*** A solution of Oxone™ (2.0 equiv) in water (4 vol) was added to a solution of the corresponding aniline **17a–d** (1.0 equiv) in DCM (1 vol). The resulting mixture was stirred vigorously at rt during 2 h. Water was added and the layers were separated. The green organic layer was washed with aq. HCl (1 M), water and brine, dried over Na_2SO_4 and concentrated to give the corresponding nitrosocompound **18a–d**, which was used without further purification.

General synthetic procedure F for compounds **20f–h**, **21**, **22b** and **22d***:* The corresponding nitrosocompound **18a–d** (1.1–1.5 equiv) and the corresponding aniline **19a–d** (1.0 equiv) were dissolved in AcOH. The mixture was stirred at 100 °C for 16–20 h protected from light. Subsequently, the solvents were removed in vacuo and the resulting dark residue was purified with automated flash chromatography (cHex/DCM) to give compound **20f–h**, **21**, **22b** or **22d** as an orange/red solid with high purity.

General synthetic procedure G for compounds ***23*f–g**, **24**, **25b** and **25d***:* The corresponding azobenzoates **20f–g**, **21**, **22b** or **22d** (1.0 equiv) were dissolved in THF. DIBAL-H (3–4 equiv, 1.0 M in THF) was added slowly at 0–5 °C. The reaction mixture was warmed slowly to rt and stirred for 2–4 h. After that, the reaction was quenched with satd. aq. NH_4Cl. Aq. Rochelle Salt (10%) and EtOAc were added and the resulting mixture was stirred at room temperature 1–2 h. The layers were separated. The organic phase was washed with brine, dried over $MgSO_4$, filtered and the solvent was removed in vacuo. The resulting residue (orange/red oil) corresponded to the expected products with high purity (**23g**, **25b**, **25c**) or were purified with automated flash chromatography (cHex/DCM) to give alcohols (**23f**, **24**) as an orange/red oil with high purity.

General synthetic procedure H for compounds **26f–h**, **27**, **28b** and **28d***:* The corresponding alcohols

23f-g, **24**, **25b** or **25d** (1.0 equiv) and Dess Martin periodinane (1.0 equiv) were dissolved in DCM. The resulting red solution was stirred for 1–2 h at rt. After that, satd. aq. $NaHCO_3$ and EtOAc were added and the layers were separated. The organic layer was washed with satd. aq. $NaHCO_3$ (twice), water and brine, dried over $MgSO_4$ and concentrated in vacuo. The residue obtained was purified with automated flash chromatography (cHex/DCM or cHex/EtOAc) to give benzaldehyde **23f–g**, **24**, **25b** or **25d** as an orange/red oil with high purity.

Detailed synthetic procedures and characterisation of compounds

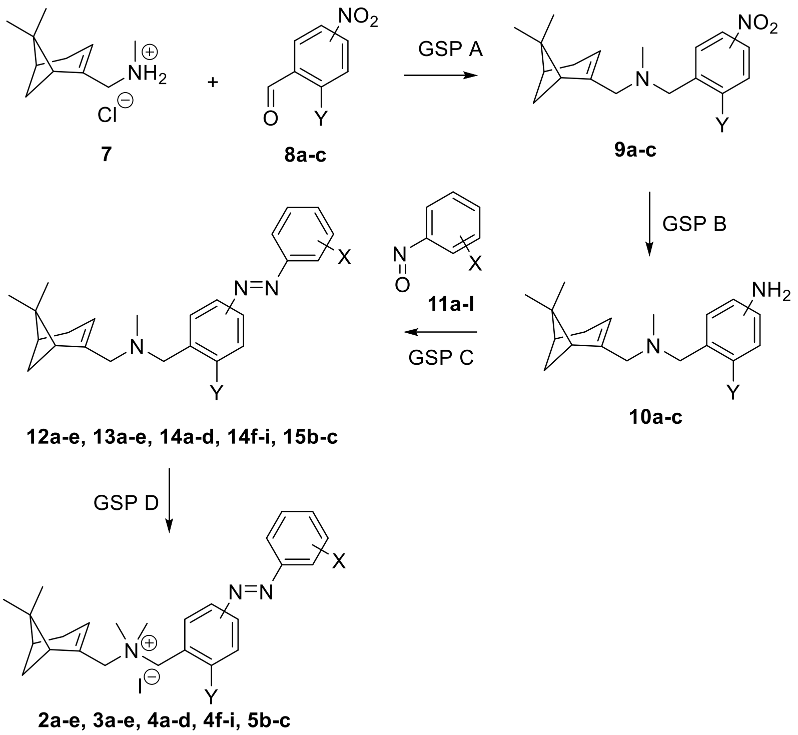

Scheme S1: Synthetic strategies for compounds **2a–e**, **3a–e**, **4a–d**, **4f–i** and **5b,c**. GSP = General Synthetic Procedure.

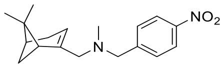

1-((1*R*,5*S*)-6,6-Dimethylbicyclo[3.1.1]hept-2-en-2-yl)-*N*-methyl-*N*-(4-nitrobenzyl)methanamine (9a): The general synthetic procedure A was used with ammonium salt **7** (2.00 g, 9.9 mmol), TEA (1.5 mL, 10.8 mmol) and 4-nitrobenzaldehyde (**8a**) (1.42 g, 9.4 mmol) in DCE (50 mL) to give the nitrocompound **9a** as a colourless oil (2.74 g, 97%). ^{1}H NMR (600 MHz, $CDCl_3$) δ 8.15 (d, J = 8.7 Hz, 2H), 7.48 (d, J = 8.7 Hz, 2H), 5.47 – 5.38 (m, 1H), 3.59 (d, J = 14.3 Hz, 1H), 3.44 (d, J = 14.2 Hz, 1H), 2.92 (app. dq, J = 13.1, 1.8 Hz, 1H), 2.84 (app. dq, J = 13.0, 1.3 Hz, 1H), 2.40 (dt, J = 8.6, 5.6 Hz, 1H), 2.33 – 2.18 (m, 3H), 2.12 (s, 3H), 2.12 – 2.05 (m, 1H), 1.29 (s, 3H), 1.11 (d, J = 8.6 Hz, 1H), 0.81 (s, 3H). ^{13}C NMR (151 MHz, $CDCl_3$) δ 148.11, 147.07, 146.13, 129.29, 123.53, 120.35, 63.87, 60.84, 44.34, 42.78, 41.00, 38.06, 31.91, 31.48, 26.40, 21.21. HPLC-PDA-MS: RT = 3.34 min, 99.1% (254 nm), PDA λ_{max} = 262 nm, MS (m/z) $[M+H]^+$ 301.15.

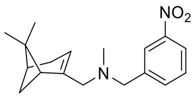

1-((1*R*,5*S*)-6,6-Dimethylbicyclo[3.1.1]hept-2-en-2-yl)-*N*-methyl-*N*-(3-nitrobenzyl)methanamine

(9b): The synthesis and characterization of this compound have been published by us [1].

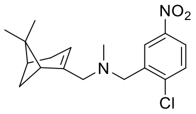

***N*-(2-Chloro-5-nitrobenzyl)-1-((1*R*,5*S*)-6,6-dimethylbicyclo[3.1.1]hept-2-en-2-yl)-*N*-methyl methanamine (9c):** The synthesis and characterization of this compound have been published by us [1].

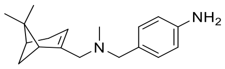

4-(((((1*R*,5*S*)-6,6-Dimethylbicyclo[3.1.1]hept-2-en-2-yl)methyl)(methyl)amino)methyl)aniline (10a): The general synthetic procedure B was used with nitrocompound **9a** (1.36 g, 4.5 mmol) and $SnCl_2 \cdot 2H_2O$ (5.10 g, 22.6 mmol) in EtOH (23 mL) to give the aniline **10a** as a yellow oil (1.23 g, quant.). ^1H NMR (600 MHz, $CDCl_3$) δ 7.08 (d, 2H), 6.64 (d, J = 8.3 Hz, 2H), 5.46 – 5.36 (m, 1H), 3.60 (br, 2H), 3.43 (d, J = 12.9 Hz, 1H), 3.26 (d, J = 13.0 Hz, 1H), 2.90 (app. dd, J = 13.1, 1.9 Hz, 1H), 2.79 (app. dd, J = 13.1, 1.4 Hz, 1H), 2.41 (dt, J = 8.6, 5.6 Hz, 1H), 2.32 – 2.17 (m, 3H), 2.11 (s, 4H), 1.32 – 1.25 (m, 4H), 1.16 (d, J = 8.6 Hz, 1H), 0.85 (s, 3H). ^{13}C NMR (151 MHz, $CDCl_3$) δ 146.52, 145.22, 130.20, 129.41, 119.80, 115.03, 63.53, 61.26, 44.36, 42.43, 41.06, 38.04, 31.98, 31.52, 26.42, 21.25. HPLC-PDA-MS: RT = 3.34 min, 99.1% (254 nm), PDA λ_{max} = 248, 280 nm, MS (m/z) $[M+H]^+$ 271.20.

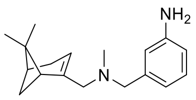

3-(((((1*R*,5*S*)-6,6-Dimethylbicyclo[3.1.1]hept-2-en-2-yl)methyl)(methyl)amino)methyl)aniline (10b): The synthesis and characterization of this compound have been published by us [1].

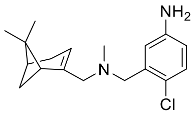

4-Chloro- 3-(((((1*R*,5*S*)-6,6-dimethylbicyclo[3.1.1]hept-2-en-2-yl)methyl)(methyl)amino)methyl) aniline (10c): The synthesis and characterization of this compound have been published by us [1].

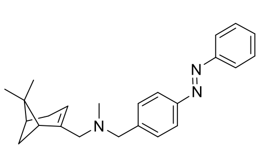

1-((1*R*,5*S*)-6,6-Dimethylbicyclo[3.1.1]hept-2-en-2-yl)-*N*-methyl-*N*-(4-((*E*)-phenyldiazenyl)benzyl) methanamine (12a): The general synthetic procedure C was used with nitrosobenzene (**11a**) (45 mg, 0.42 mmol) and the aniline **10a** (103 mg, 0.38 mmol) in DCM (2 mL) to give the azocompound **12a** as an orange oil (70 mg, 51%). ^1H NMR (500 MHz, $CDCl_3$) δ 7.91 (d, J = 7.1 Hz, 2H), 7.87 (d, J = 8.3 Hz, 2H), 7.52 (t, J = 7.4 Hz, 2H), 7.49 – 7.43 (m, 3H), 5.44 (br, 1H), 3.61 (d, J = 13.6 Hz, 1H), 3.44 (d, J = 13.6 Hz, 1H), 2.96 (d, J = 13.1 Hz, 1H), 2.86 (d, J = 13.0 Hz, 1H), 2.43 (dt, J = 8.5, 5.6 Hz, 1H), 2.39 – 2.20 (m, 3H),

2.17 (s, 3H), 2.14 – 2.06 (m, 1H), 1.31 (s, 3H), 1.16 (d, J = 8.6 Hz, 1H), 0.85 (s, 3H). ^{13}C NMR (126 MHz, $CDCl_3$) δ 152.84, 151.87, 146.40, 143.38, 130.95, 129.63, 129.21, 122.90, 122.85, 120.13, 63.81, 61.39, 44.35, 42.78, 41.03, 38.11, 31.99, 31.54, 26.43, 21.27. HPLC-PDA-MS: RT = 4.64 min, 96.9% (254 nm), PDA λ_{max} = 320 nm, MS (m/z) $[M+H]^+$ 360.25.

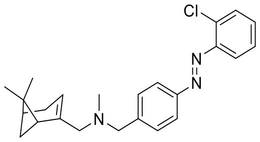

***N*-(4-((*E*)-(2-Chlorophenyl)diazenyl)benzyl)-1-((1*R*,5*S*)-6,6-dimethylbicyclo[3.1.1]hept-2-en-2-yl)-*N*-methylmethanamine (12b):** The general synthetic procedure C was used with 2-chloroaniline (125 µL, 1.19 mmol) in DCM (3.6 mL) and Oxone® (1.49 g, 2.4 mmol) in water (14.4 mL), to form the corresponding nitrosocompound **11b**, and the aniline **10a** (166 mg, 0.61 mmol) in DCM (7 mL) to give the azocompound **12b** as an orange oil (167 mg, 69%). ^{1}H NMR (500 MHz, $CDCl_3$) δ 7.93 (d, J = 8.0 Hz, 2H), 7.69 (dd, J = 7.9, 1.6 Hz, 1H), 7.56 (dd, J = 7.9, 1.3 Hz, 1H), 7.48 (d, J = 8.0 Hz, 2H), 7.39 (td, J = 7.6, 1.7 Hz, 1H), 7.34 (td, J = 7.5, 1.4 Hz, 1H), 5.44 (br, 1H), 3.61 (d, J = 13.7 Hz, 1H), 3.44 (d, J = 12.7 Hz, 1H), 2.96 (d, J = 13.0 Hz, 1H), 2.86 (d, J = 12.1 Hz, 1H), 2.43 (dt, J = 8.5, 5.6 Hz, 1H), 2.38 – 2.20 (m, 3H), 2.17 (s, 3H), 2.14 – 2.05 (m, 1H), 1.31 (s, 3H), 1.16 (d, J = 8.6 Hz, 1H), 0.84 (s, 3H). ^{13}C NMR (126 MHz, $CDCl_3$) δ 152.02, 148.93, 146.47, 144.19, 135.27, 131.61, 130.79, 129.66, 127.42, 123.39, 120.11, 117.73, 63.83, 61.40, 44.36, 42.81, 41.03, 38.12, 31.99, 31.56, 26.43, 21.28. HPLC-PDA-MS: RT = 4.80 min, 89.9% (254 nm), PDA λ_{max} = 323 nm, MS (m/z) $[M+H]^+$ 394.20.

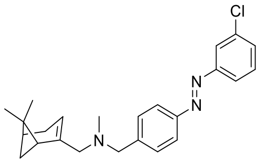

***N*-(4-((*E*)-(3-Chlorophenyl)diazenyl)benzyl)-1-((1*R*,5*S*)-6,6-dimethylbicyclo[3.1.1]hept-2-en-2-yl)-*N*-methylmethanamine (12c):** The general synthetic procedure C was used with 3-chloroaniline (37 µL, 0.35 mmol) in DCM (2.0 mL) and Oxone® (424 mg, 0.69 mmol) in water (4.0 mL), to form the corresponding nitrosocompound **11c**, and the aniline **10a** (70 mg, 0.26 mmol) in DCM (3 mL) to give the azocompound **12c** as an orange oil (65 mg, 64%). ^{1}H NMR (600 MHz, $CDCl_3$) δ 7.89 (t, J = 1.9 Hz, 1H), 7.87 (d, J = 8.3 Hz, 2H), 7.82 (dt, J = 7.4, 1.7 Hz, 1H), 7.47 (d, J = 8.0 Hz, 2H), 7.46 – 7.41 (m, 2H), 5.46 – 5.42 (m, 1H), 3.60 (d, J = 13.7 Hz, 1H), 3.44 (d, J = 13.7 Hz, 1H), 2.96 (dd, J = 13.0, 2.0 Hz, 1H), 2.86 (d, J = 13.0 Hz, 1H), 2.43 (dt, J = 8.6, 5.6 Hz, 1H), 2.36 – 2.28 (m, 2H), 2.24 (app. dd, J = 17.6, 3.5 Hz, 1H), 2.17 (s, 3H), 2.14 – 2.09 (m, 1H), 1.31 (s, 3H), 1.16 (d, J = 8.6 Hz, 1H), 0.85 (s, 3H). ^{13}C NMR (151 MHz, $CDCl_3$) δ 153.69, 151.60, 146.41, 144.10, 135.24, 130.62, 130.23, 129.63, 123.07, 122.44, 121.84, 120.13, 63.87, 61.41, 44.41, 42.81, 41.08, 38.11, 31.99, 31.55, 26.46, 21.28. HPLC-PDA-MS: RT = 5.04 min, 97.0% (254 nm), PDA λ_{max} = 319 nm, MS (m/z) $[M+H]^+$ 394.20.

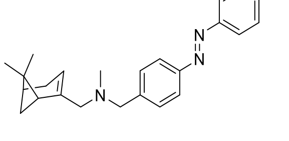

***N*-(4-((*E*)-(4-Chlorophenyl)diazenyl)benzyl)-1-((1*R*,5*S*)-6,6-dimethylbicyclo[3.1.1]hept-2-en-2-yl)-*N*-methylmethanamine (12d):** The general synthetic procedure C was used with 4-chloroaniline (152 mg, 1.19 mmol) in DCM (3.6 mL) and Oxone® (1.50 g, 2.4 mmol) in water (14.4 mL), to form the

corresponding nitrosocompound **11d**, and the aniline **10a** (162 mg, 0.60 mmol) in DCM (7 mL) to give the azocompound **12d** as an orange oil (102 mg, 43%). ^{1}H NMR (500 MHz, $CDCl_3$) δ 7.86 (d, J = 8.6 Hz, 4H), 7.48 (d, J = 7.2 Hz, 2H), 7.47 (d, J = 7.5 Hz, 2H), 5.44 (br, 1H), 3.60 (d, J = 13.7 Hz, 1H), 3.44 (d, J = 13.7 Hz, 1H), 2.95 (d, J = 13.1 Hz, 1H), 2.85 (d, J = 13.0 Hz, 1H), 2.43 (dt, J = 8.0, 5.6 Hz, 1H), 2.37 – 2.20 (m, 3H), 2.16 (s, 3H), 2.14 – 2.07 (m, 1H), 1.31 (s, 3H), 1.16 (d, J = 8.4 Hz, 1H), 0.85 (s, 3H). ^{13}C NMR (126 MHz, $CDCl_3$) δ 151.40, 150.91, 146.15, 143.56, 136.54, 129.38, 129.19, 123.92, 122.68, 119.86, 63.60, 61.14, 44.08, 42.57, 40.78, 37.85, 31.73, 31.28, 26.18, 21.02. HPLC-PDA-MS: RT = 4.87 min, 94.9% (254 nm), PDA λ_{max} = 326 nm, MS (m/z) $[M+H]^+$ 394.20.

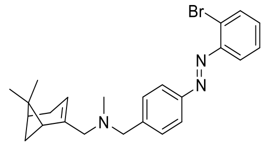

***N*-(2-((*E*)-(2-Bromophenyl)diazenyl)benzyl)-1-((1*R*,5*S*)-6,6-dimethylbicyclo[3.1.1]hept-2-en-2-yl)-*N*-methylmethanamine (12e):** The general synthetic procedure C was used with 2-bromoaniline (133 µL, 1.22 mmol) in DCM (3.3 mL) and Oxone® (1.54 g, 2.5 mmol) in water (13.2 mL), to form the corresponding nitrosocompound **11e**, and the aniline **10a** (161 mg, 0.60 mmol) in DCM (7 mL) to give the azocompound **12e** as an orange oil (216 mg, 83%). ^{1}H NMR (600 MHz, $CDCl_3$) δ 7.93 (d, J = 8.3 Hz, 2H), 7.75 (dd, J = 8.0, 1.3 Hz, 1H), 7.67 (dd, J = 8.0, 1.6 Hz, 1H), 7.48 (d, J = 8.2 Hz, 2H), 7.39 (ddd, J = 8.1, 7.3, 1.3 Hz, 1H), 7.30 (td, J = 8.0, 1.7 Hz, 1H), 5.47 – 5.40 (m, 1H), 3.60 (d, J = 13.7 Hz, 1H), 3.44 (d, J = 13.7 Hz, 1H), 2.96 (app. dq, J = 13.0, 1.8 Hz, 1H), 2.86 (d, J = 13.1 Hz, 1H), 2.43 (dt, J = 8.6, 5.6 Hz, 1H), 2.34 (td, J = 5.8, 1.5 Hz, 1H), 2.33 – 2.28 (m, 1H), 2.24 (app. dd, J = 18.3, 2.7 Hz, 1H), 2.16 (s, 3H), 2.13 – 2.09 (m, 1H), 1.32 (s, 3H), 1.16 (d, J = 8.6 Hz, 1H), 0.85 (s, 3H). ^{13}C NMR (151 MHz, $CDCl_3$) δ 151.90, 149.89, 146.45, 144.18, 133.84, 131.79, 129.65, 128.10, 125.68, 123.44, 120.10, 117.94, 63.87, 61.43, 44.39, 42.80, 41.09, 38.11, 32.00, 31.55, 26.46, 21.28. HPLC-PDA-MS: RT = 4.81 min, 95.8% (254 nm), PDA λ_{max} = 323 nm, MS (m/z) $[M+H]^+$ 438.20, 440.20.

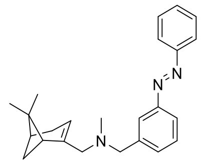

1-((1*R*,5*S*)-6,6-Dimethylbicyclo[3.1.1]hept-2-en-2-yl)-*N*-methyl-*N*-(3-((*E*)-phenyldiazenyl)benzyl) methanamine (13a): The general synthetic procedure C was used with nitrosobenzene (**11a**) (47 mg, 0.44 mmol) and the aniline **10b** (107 mg, 0.40 mmol) in DCM (2 mL) to give the azocompound **13a** as an orange oil (95 mg, 67%). ^{1}H NMR (500 MHz, $CDCl_3$) δ 7.93 (d, J = 7.0 Hz, 2H), 7.87 (s, 1H), 7.84 – 7.75 (m, 1H), 7.53 (t, J = 7.4 Hz, 2H), 7.50 – 7.44 (m, 3H), 5.45 (br, 1H), 3.63 (d, J = 13.4 Hz, 1H), 3.48 (d, J = 13.3 Hz, 1H), 2.98 (app. dd, J = 13.0, 2.0 Hz, 1H), 2.88 (d, J = 13.0 Hz, 1H), 2.44 (dt, J = 8.6, 5.6 Hz, 1H), 2.36 (td, J = 5.7, 1.4 Hz, 1H), 2.28 (app. q, J = 17.7 Hz, 2H), 2.18 (s, 3H), 2.15 – 2.06 (m, 1H), 1.32 (s, 3H), 1.18 (d, J = 8.5 Hz, 1H), 0.86 (s, 3H). ^{13}C NMR (126 MHz, $CDCl_3$) δ 152.81, 152.78, 146.42, 141.08, 131.69, 131.03, 129.21, 129.02, 123.33, 122.94, 121.63, 120.07, 63.83, 61.40, 44.34, 42.72, 41.04, 38.10, 32.00, 31.54, 26.44, 21.28. HPLC-PDA-MS: RT = 4.66 min, 96.1% (254 nm), PDA λ_{max} = 318 nm, MS (m/z) $[M+H]^+$ 360.20.

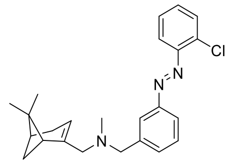

***N*-(3-((*E*)-(2-Chlorophenyl)diazenyl)benzyl)-1-((1*R*,5*S*)-6,6-dimethylbicyclo[3.1.1]hept-2-en-2-yl)-*N*-methylmethanamine (13b):** The general synthetic procedure C was used with 2-chloroaniline (63 µL, 0.60 mmol) in DCM (1.8 mL)and Oxone® (0.74 g, 1.2 mmol) in water (7.1 mL), to form the corresponding nitrosocompound **11b**, and the aniline **10b** (80 mg, 0.30 mmol) in DCM (1.5 mL) to give the azocompound **13b** as an orange oil (91 mg, 78%). ^{1}H NMR (600 MHz, $CDCl_3$) δ 7.92 (t, J = 1.7 Hz, 1H), 7.85 (dt, J = 6.8, 2.1 Hz, 1H), 7.70 (dd, J = 7.9, 1.7 Hz, 1H), 7.56 (dd, J = 7.9, 1.4 Hz, 1H), 7.50 – 7.44 (m, 2H), 7.39 (td, J = 7.6, 1.8 Hz, 1H), 7.35 (td, J = 7.6, 1.5 Hz, 1H), 5.45 (br, 1H), 3.64 (d, J = 13.4 Hz, 1H), 3.48 (d, J = 13.4 Hz, 1H), 2.98 (app. dd, J = 13.1, 2.0 Hz, 1H), 2.88 (d, J = 13.1 Hz, 1H), 2.44 (dt, J = 8.6, 5.6 Hz, 1H), 2.36 (td, J = 5.7, 1.4 Hz, 1H), 2.31 (app. d, J = 17.6 Hz, 1H), 2.25 (app. dd, J = 17.5, 2.8 Hz, 1H), 2.18 (s, 3H), 2.15 – 2.08 (m, 1H), 1.31 (s, 3H), 1.17 (d, J = 8.6 Hz, 1H), 0.85 (s, 3H). ^{13}C NMR (151 MHz, $CDCl_3$) δ 152.97, 148.95, 146.46, 141.24, 135.35, 132.23, 131.66, 130.80, 129.07, 127.40, 124.02, 121.92, 120.08, 117.74, 63.87, 61.33, 44.38, 42.71, 41.09, 38.11, 32.01, 31.56, 26.46, 21.29. HPLC-PDA-MS: RT = 4.84 min, 94.0% (254 nm), PDA λ_{max} = 321 nm, MS (m/z) $[M+H]^+$ 394.20.

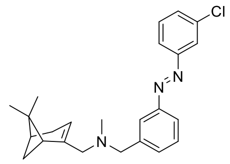

***N*-(3-((*E*)-(3-Chlorophenyl)diazenyl)benzyl)-1-((1*R*,5*S*)-6,6-dimethylbicyclo[3.1.1]hept-2-en-2-yl)-*N*-methylmethanamine (13c):** The general synthetic procedure C was used with 3-chloroaniline (37 µL, 0.60 mmol) in DCM (2.0 mL) and Oxone® (424 mg, 0.69 mmol) in water (4.0 mL), to form the corresponding nitrosocompound **11c**, and the aniline **10b** (70 mg, 0.26 mmol) in DCM (3 mL) to give the azocompound **13c** as an orange oil (72 mg, 71%). ^{1}H NMR (500 MHz, $CDCl_3$) δ 7.90 (t, J = 1.9 Hz, 1H), 7.86 (s, 1H), 7.84 (dt, J = 7.2, 1.9 Hz, 1H), 7.82 – 7.78 (m, 1H), 7.51 – 7.41 (m, 4H), 5.45 (br, 1H), 3.63 (d, J = 13.4 Hz, 1H), 3.48 (d, J = 13.4 Hz, 1H), 2.98 (d, J = 13.1 Hz, 1H), 2.88 (d, J = 13.1 Hz, 1H), 2.44 (dt, J = 8.5, 5.6 Hz, 1H), 2.35 (td, J = 5.8, 1.4 Hz, 1H), 2.28 (app. q, J = 19.0, 18.5 Hz, 2H), 2.18 (s, 3H), 2.15 – 2.07 (m, 1H), 1.31 (s, 3H), 1.17 (d, J = 8.5 Hz, 1H), 0.85 (s, 3H). ^{13}C NMR (126 MHz, $CDCl_3$) δ 153.61, 152.51, 146.38, 141.25, 135.24, 132.22, 130.74, 130.26, 129.11, 123.45, 122.51, 121.90, 121.86, 120.10, 63.82, 61.33, 44.35, 42.72, 41.03, 38.11, 32.00, 31.55, 26.44, 21.28. HPLC-PDA-MS: RT = 4.99 min, 97.0% (254 nm), PDA λ_{max} = 317 nm, MS (m/z) $[M+H]^+$ 394.20.

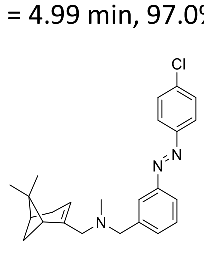

***N*-(3-((*E*)-(4-Chlorophenyl)diazenyl)benzyl)-1-((1*R*,5*S*)-6,6-dimethylbicyclo[3.1.1]hept-2-en-2-yl)-*N*-methylmethanamine (13d):** The general synthetic procedure C was used with 4-chloroaniline (152 mg, 1.19 mmol) in DCM (3.6 mL) and Oxone® (1.50 g, 2.4 mmol) in water (14.4 mL), to form the corresponding nitrosocompound **11d**, and the aniline **10b** (166 mg, 0.61 mmol) in DCM (7 mL) to give the azocompound **13d** as an orange oil (79 mg, 33%). ^{1}H NMR (500 MHz, $CDCl_3$) δ 7.88 (d, J = 8.6 Hz,

2H), 7.85 (s, 1H), 7.79 (td, J = 4.8, 4.2, 2.0 Hz, 1H), 7.49 (d, J = 8.7 Hz, 2H), 7.48 – 7.42 (m, 2H), 5.45 (br, 1H), 3.62 (d, J = 13.4 Hz, 1H), 3.47 (d, J = 13.4 Hz, 1H), 2.97 (d, J = 13.1 Hz, 1H), 2.87 (d, J = 13.1 Hz, 1H), 2.43 (dt, J = 8.4, 5.6 Hz, 1H), 2.35 (t, J = 5.9 Hz, 1H), 2.26 (app. q, J = 18.6, 17.8 Hz, 2H), 2.17 (s, 3H), 2.14 – 2.06 (m, 1H), 1.31 (s, 3H), 1.17 (d, J = 8.7 Hz, 1H), 0.85 (s, 3H). ^{13}C NMR (126 MHz, $CDCl_3$) δ 152.60, 151.14, 146.42, 141.22, 131.98, 129.46, 129.08, 124.23, 123.34, 121.77, 120.08, 63.83, 61.36, 44.34, 42.73, 41.03, 38.11, 32.00, 31.55, 26.44, 21.28. HPLC-PDA-MS: RT = 4.87 min, 98.4% (254 nm), PDA λ_{max} = 234 nm, MS (m/z) $[M+H]^+$ 394.20.

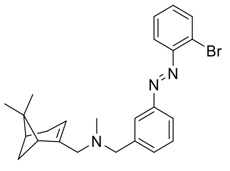

***N*-(3-((*E*)-(2-Bromophenyl)diazenyl)benzyl)-1-((1*R*,5*S*)-6,6-dimethylbicyclo[3.1.1]hept-2-en-2-yl)-*N*-methylmethanamine (13e):** The synthesis and characterization of this compound have been published by us [1].

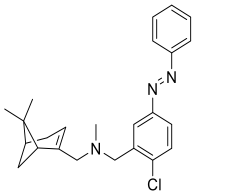

***N*-(2-Chloro-5-((*E*)-phenyldiazenyl)benzyl)-1-((1*R*,5*S*)-6,6-dimethylbicyclo[3.1.1]hept-2-en-2-yl)-*N*-methylmethanamine (14a):** The general synthetic procedure C was used with nitrosobenzene (**11a**) (111 mg, 1.04 mmol) and the aniline **10c** (155 mg, 0.51 mmol) in DCM (5.5 mL) to give the azocompound **14a** as an orange oil (118 mg, 59%). ^{1}H NMR (500 MHz, $CDCl_3$) δ 8.08 (s, 1H), 7.90 (d, J = 8.1 Hz, 2H), 7.71 (d, J = 8.3 Hz, 1H), 7.50 (t, J = 7.4 Hz, 2H), 7.47 – 7.41 (m, 2H), 5.43 (br, 1H), 3.67 (d, J = 14.6 Hz, 1H), 3.57 (d, J = 14.6 Hz, 1H), 3.00 (d, J = 13.1 Hz, 1H), 2.92 (d, J = 13.1 Hz, 1H), 2.45 – 2.36 (m, 1H), 2.34 (t, J = 5.8 Hz, 1H), 2.31 – 2.17 (m, 5H), 2.11 – 2.01 (m, 1H), 1.26 (s, 3H), 1.13 (d, J = 10.1 Hz, 1H), 0.80 (s, 3H). ^{13}C NMR (126 MHz, $CDCl_3$) δ 152.67, 151.28, 146.39, 138.51, 136.68, 131.24, 130.09, 129.23, 125.69, 123.02, 121.52, 120.06, 64.03, 58.22, 44.37, 42.89, 41.00, 38.09, 32.00, 31.55, 26.44, 21.24. HPLC-PDA-MS: RT = 4.87 min, 82.6% (254 nm), PDA λ_{max} = 324 nm, MS (m/z) $[M+H]^+$ 394.20.

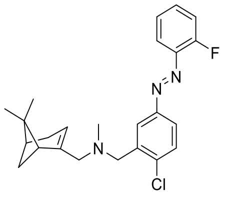

***N*-(2-Chloro-5-((*E*)-(2-fluorophenyl)diazenyl)benzyl)-1-((1*R*,5*S*)-6,6-dimethylbicyclo[3.1.1]hept-2-en-2-yl)-*N*-methylmethanamine (14b):** The general synthetic procedure C was used with 2-fluoroaniline (97 µL, 1.0 mmol) in DCM (2.7 mL) and Oxone® (1.26 g, 2.1 mmol) in water (10.7 mL), to form the corresponding nitrosocompound **11f**, and the aniline **10c** (153 mg, 0.50 mmol) in DCM (5.5 mL) to give the azocompound **14b** as an orange oil (102 mg, 49%). ^{1}H NMR (600 MHz, $CDCl_3$) δ 8.14 (d, J = 2.4 Hz, 1H), 7.79 – 7.73 (m, 2H), 7.50 – 7.43 (m, 2H), 7.32 – 7.25 (m, 1H), 7.23 (t, J = 7.7 Hz, 1H), 5.53 – 5.39 (m, 1H), 3.70 (d, J = 14.6 Hz, 1H), 3.60 (d, J = 14.6 Hz, 1H), 3.03 (d, J = 13.2 Hz, 1H), 2.95 (d,

J = 13.1 Hz, 1H), 2.42 (dt, J = 8.6, 5.6 Hz, 1H), 2.36 (t, J = 5.2 Hz, 1H), 2.31 (app. d, J = 17.6 Hz, 1H), 2.28 – 2.18 (m, 4H), 2.13 – 2.06 (m, 1H), 1.29 (s, 3H), 1.15 (d, J = 8.6 Hz, 1H), 0.83 (s, 3H). ^{13}C NMR (151 MHz, $CDCl_3$) δ 161.15, 159.44, 151.52, 146.43, 140.78, 140.74, 138.66, 137.21, 132.76, 132.70, 130.16, 126.44, 124.44, 124.41, 121.35, 120.10, 117.88, 117.31, 117.17, 64.06, 58.17, 44.43, 42.88, 41.06, 38.09, 31.99, 31.57, 26.38, 21.24. HPLC-PDA-MS: RT = 4.82 min, 90.6% (254 nm), PDA λ_{max} = 327 nm, MS (m/z) $[M+H]^+$ 412.20.

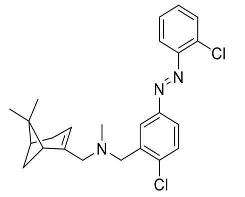

***N*-(2-Chloro-5-((*E*)-(2-chlorophenyl)diazenyl)benzyl)-1-((1*R*,5*S*)-6,6-dimethylbicyclo[3.1.1]hept-2-en-2-yl)-*N*-methylmethanamine (14c):** The general synthetic procedure C was used with 2-chloroaniline (105 µL, 1.0 mmol) in DCM (2.7 mL) and Oxone® (1.26 g, 2.1 mmol) in water (10.7 mL), to form the corresponding nitrosocompound **11b**, and the aniline **10c** (153 mg, 0.50 mmol) in DCM (5.5 mL) to give the azocompound **14c** as an orange oil (125 mg, 58%). ^{1}H NMR (600 MHz, $CDCl_3$) δ 8.16 (s, 1H), 7.78 (dd, J = 8.5, 2.4 Hz, 1H), 7.71 (dd, J = 8.0, 1.7 Hz, 1H), 7.56 (dd, J = 8.0, 1.3 Hz, 1H), 7.47 (d, J = 8.5 Hz, 1H), 7.40 (td, J = 7.6, 1.7 Hz, 1H), 7.35 (td, J = 8.0, 1.3 Hz, 1H), 5.47 (br, 1H), 3.71 (d, J = 14.6 Hz, 1H), 3.60 (d, J = 14.6 Hz, 1H), 3.04 (d, J = 13.2 Hz, 1H), 2.96 (d, J = 13.1 Hz, 1H), 2.41 (dt, J = 8.5, 5.6 Hz, 1H), 2.39 – 2.34 (m, 1H), 2.31 (app. d, J = 17.3 Hz, 1H), 2.27 – 2.20 (m, 4H), 2.12 – 2.06 (m, 1H), 1.28 (s, 3H), 1.15 (d, J = 8.6 Hz, 1H), 0.83 (s, 3H). ^{13}C NMR (151 MHz, $CDCl_3$) δ 151.51, 148.79, 146.44, 138.73, 137.33, 135.62, 64.14, 58.13, 38.11, 32.01, 31.57. δ. HPLC-PDA-MS: RT = 5.08 min, 76.2% (254 nm), PDA λ_{max} = 326 nm, MS (m/z) $[M+H]^+$ 428.20.

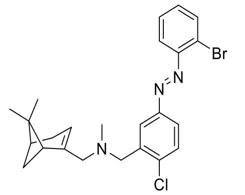

***N*-(5-((*E*)-(2-Bromophenyl)diazenyl)-2-chlorobenzyl)-1-((1*R*,5*S*)-6,6-dimethylbicyclo[3.1.1]hept-2-en-2-yl)-*N*-methylmethanamine (14d):** The synthesis and characterization of this compound have been published by us[1].

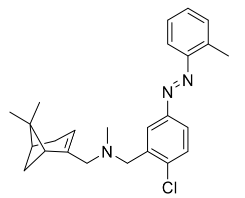

***N*-(2-Chloro-5-((*E*)-*o*-tolyldiazenyl)benzyl)-1-((1*R*,5*S*)-6,6-dimethylbicyclo[3.1.1]hept-2-en-2-yl)-*N*-methylmethanamine (14f):** The general synthetic procedure C was used with *o*-toluidine (107 µL, 1.00 mmol) in DCM (2.7 mL) and Oxone® (1.26 g, 2.1 mmol) in water (10.7 mL), to form the corresponding nitrosocompound **11g**, and the aniline **10c** (151 mg, 0.50 mmol) in DCM (5.5 mL) to give the azocompound **14f** as an orange oil (47 mg, 23%). ^{1}H NMR (600 MHz, $CDCl_3$) δ 8.10 (d, J = 2.4 Hz, 1H), 7.72 (dd, J = 8.4, 2.4 Hz, 1H), 7.64 (d, J = 8.0 Hz, 1H), 7.46 (d, J = 8.4 Hz, 1H), 7.39 – 7.32 (m, 2H), 7.30 – 7.26 (m, 1H), 5.46 (br, 1H), 3.70 (d, J = 14.6 Hz, 1H), 3.60 (d, J = 14.6 Hz, 1H), 3.04 (d, J = 13.2 Hz, 1H),

2.96 (d, J = 13.1 Hz, 1H), 2.74 (s, 3H), 2.41 (dt, J = 8.5, 5.6 Hz, 1H), 2.36 (t, J = 5.4 Hz, 1H), 2.30 (app. d, J = 17.9 Hz, 1H), 2.27 – 2.18 (m, 4H), 2.14 – 2.05 (m, 1H), 1.28 (s, 3H), 1.15 (d, J = 8.5 Hz, 1H), 0.83 (s, 3H).^{13}C NMR (151 MHz, $CDCl_3$) δ 151.72, 150.79, 146.49, 138.54, 138.39, 136.46, 131.42, 131.20, 130.04, 126.58, 125.62, 121.67, 120.02, 115.55, 64.17, 58.21, 44.39, 42.82, 41.07, 38.11, 31.99, 31.56, 26.43, 21.23, 17.70. HPLC-PDA-MS: RT = 5.15 min, 97.8% (254 nm), PDA λ_{max} = 325 nm, MS (m/z) $[M+H]^+$ 408.25.

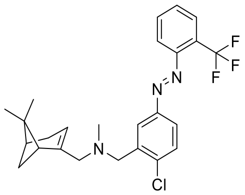

***N*-(2-Chloro-5-((*E*)-(2-(trifluoromethyl)phenyl)diazenyl)benzyl)-1-((1*R*,5*S*)-6,6-dimethylbicyclo [3.1.1]hept-2-en-2-yl)-*N*-methylmethanamine (14g):** The general synthetic procedure C was used with 2-(trifluoromethyl)aniline (126 µL, 1.00 mmol) in DCM (2.7 mL) and Oxone® (1.26 g, 2.1 mmol) in water (10.7 mL), to form the corresponding nitrosocompound **11h**, and the aniline **10c** (155 mg, 0.51 mmol) in DCM (5.5 mL) to give the azocompound **14g** as an orange oil (116 mg, 49%). ^{1}H NMR (500 MHz, $CDCl_3$) δ 8.18 (s, 1H), 7.89 – 7.80 (m, 2H), 7.76 (d, J = 8.5 Hz, 1H), 7.66 (t, J = 7.8 Hz, 1H), 7.57 (t, J = 7.6 Hz, 1H), 7.48 (d, J = 8.5 Hz, 1H), 5.47 (br, 1H), 3.70 (d, J = 14.8 Hz, 1H), 3.60 (d, J = 14.8 Hz, 1H), 3.06 (d, J = 13.2 Hz, 1H), 2.97 (d, J = 13.1 Hz, 1H), 2.47 – 2.38 (m, 1H), 2.37 (t, J = 5.9 Hz, 1H), 2.34 – 2.16 (m, 5H), 2.13 – 2.05 (m, 1H), 1.28 (s, 3H), 1.14 (d, J = 8.4 Hz, 1H), 0.82 (s, 3H). ^{13}C NMR (126 MHz, $CDCl_3$) δ 151.37, 149.53, 146.45, 138.80, 137.61, 132.69, 130.63, 130.26, 129.09, 128.84, 128.59, 128.34, 127.37, 126.89, 126.78, 126.74, 126.70, 126.65, 125.20, 123.02, 121.46, 120.84, 120.08, 116.29, 64.24, 58.07, 44.26, 42.74, 41.02, 38.09, 31.95, 31.55, 26.34, 21.20. HPLC-PDA-MS: RT = 5.15 min, 92.8% (256 nm), PDA λ_{max} = 324 nm, MS (m/z) $[M+H]^+$ 462.20.

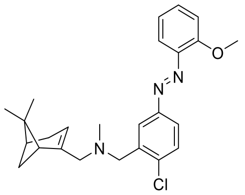

***N*-(2-Chloro-5-((*E*)-(2-methoxyphenyl)diazenyl)benzyl)-1-((1*R*,5*S*)-6,6-dimethylbicyclo [3.1.1]hept-2-en-2-yl)-*N*-methylmethanamine (14h):** The general synthetic procedure C was used with 2-methoxyaniline (113 µL, 1.00 mmol) in DCM (2.7 mL) and Oxone® (1.26 g, 2.1 mmol) in water (10.7 mL), to form the corresponding nitrosocompound **11i**, and the aniline **10c** (159 mg, 0.52 mmol) in DCM (5.5 mL) to give the azocompound **14h** as an orange oil (88 mg, 40%). ^{1}H NMR (500 MHz, $CDCl_3$) δ 8.08 (t, J = 2.1 Hz, 1H), 7.70 (d, J = 8.5 Hz, 1H), 7.67 (d, J = 7.8 Hz, 1H), 7.49 – 7.40 (m, 2H), 7.10 (d, J = 8.3 Hz, 1H), 7.02 (t, J = 7.6 Hz, 1H), 5.46 (s, 1H), 3.70 (d, J = 14.4 Hz, 1H), 3.59 (d, J = 14.3 Hz, 1H), 3.03 (d, J = 13.2 Hz, 1H), 2.94 (d, J = 13.1 Hz, 1H), 2.40 (app. qd, J = 6.5, 5.7, 3.6 Hz, 1H), 2.34 (t, J = 6.0 Hz, 1H), 2.33 – 2.17 (m, 5H), 2.14 – 2.04 (m, 1H), 1.28 (s, 3H), 1.15 (d, J = 8.5 Hz, 1H), 0.82 (s, 3H). ^{13}C NMR (126 MHz, $CDCl_3$) δ 157.15, 151.83, 146.39, 142.28, 138.33, 136.54, 132.77, 130.07, 127.21, 120.90, 120.37, 120.04, 117.08, 112.85, 64.09, 58.23, 56.44, 44.34, 42.77, 40.99, 38.07, 31.99, 31.54, 26.38, 21.21. HPLC-PDA-MS: RT = 4.63 min, 98.0% (254 nm), PDA λ_{max} = 324, 363 nm, MS (m/z) $[M+H]^+$ 424.25.

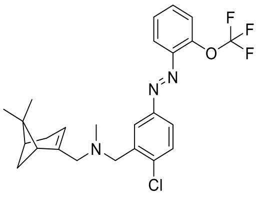

N-(2-Chloro-5-((E)-(2-(trifloromethoxy)phenyl)diazenyl)benzyl)-1-((1R,5S)-6,6-dimethylbicyclo [3.1.1]hept-2-en-2-yl)-N-methylmethanamine (14i): The general synthetic procedure C was used with 2-(trifluoromethoxy)aniline (136 µL, 1.00 mmol) in DCM (2.7 mL) and Oxone® (1.26 g, 2.1 mmol) in water (10.7 mL), to form the corresponding nitrosocompound **11j**, and the aniline **10c** (153 mg, 0.50 mmol) in DCM (5.5 mL) to give the azocompound **14i** as an orange oil (152 mg, 63%). ^{1}H NMR (600 MHz, $CDCl_3$) δ 8.15 (d, J = 2.0 Hz, 1H), 7.79 (dd, J = 8.1, 1.7 Hz, 1H), 7.75 (dd, J = 8.5, 2.4 Hz, 1H), 7.52 (ddd, J = 8.7, 7.3, 1.7 Hz, 1H), 7.47 (d, J = 8.5 Hz, 1H), 7.44 (dt, J = 8.2, 1.4 Hz, 1H), 7.41 (ddd, J = 8.4, 7.3, 1.3 Hz, 1H), 5.46 (br, 1H), 3.70 (d, J = 14.6 Hz, 1H), 3.60 (d, J = 14.7 Hz, 1H), 3.05 (d, J = 13.1 Hz, 1H), 2.96 (d, J = 13.2 Hz, 1H), 2.40 (dt, J = 8.6, 5.6 Hz, 1H), 2.34 (t, J = 5.3 Hz, 1H), 2.30 (app. d, J = 17.7 Hz, 1H), 2.27 – 2.18 (m, 4H), 2.13 – 2.05 (m, 1H), 1.27 (s, 3H), 1.14 (d, J = 8.6 Hz, 1H), 0.82 (s, 3H). ^{13}C NMR (151 MHz, $CDCl_3$) δ 151.51, 147.00, 146.42, 145.12, 138.74, 137.48, 132.12, 130.23, 127.69, 126.95, 123.34, 123.20, 121.63, 121.04, 120.05, 119.93, 118.22, 117.63, 64.19, 58.13, 44.38, 42.79, 41.06, 38.09, 31.96, 31.56, 26.37, 21.20. HPLC-PDA-MS: RT = 5.24 min, 87.8% (254 nm), PDA λ_{max} = 324 nm, MS (m/z) $[M+H]^+$ 478.25.

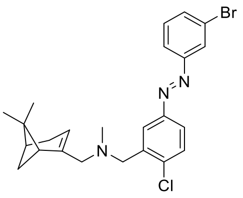

N-(5-((E)-(3-Bromophenyl)diazenyl)-2-chlorobenzyl)-1-((1R,5S)-6,6-dimethylbicyclo[3.1.1]hept-2-en-2-yl)-N-methylmethanamine (15b): The general synthetic procedure C was used with 3-bromoaniline (109 µL, 1.00 mmol) in DCM (2.7 mL) and Oxone® (1.26 g, 2.1 mmol) in water (10.7 mL), to form the corresponding nitrosocompound **11k**, and the aniline **10c** (153 mg, 0.50 mmol) in DCM (5.5 mL) to give the azocompound **15b** as an orange oil (161 mg, 68%). ^{1}H NMR (300 MHz, $CDCl_3$ δ 8.12 (d, J = 2.4 Hz, 1H), 8.06 (t, J = 1.9 Hz, 1H), 7.88 (ddd, J = 8.0, 1.6, 0.9 Hz, 1H), 7.73 (dd, J = 8.5, 2.4 Hz, 1H), 7.60 (ddd, J = 7.9, 2.0, 1.0 Hz, 1H), 7.47 (d, J = 8.5 Hz, 1H), 7.41 (t, J = 7.9 Hz, 1H), 5.46 (br, 1H), 3.70 (d, J = 14.8 Hz, 1H), 3.57 (s, 1H), 3.03 (d, J = 12.5 Hz, 1H), 2.94 (d, J = 13.1 Hz, 1H), 2.48 – 2.19 (m, 7H), 2.16 – 2.03 (m, 1H), 1.30 (s, 3H), 1.15 (d, J = 8.3 Hz, 1H), 0.83 (s, 3H). ^{13}C NMR (126 MHz, $CDCl_3$) δ 153.54, 150.98, 146.35, 138.71, 137.26, 133.82, 130.60, 130.18, 125.73, 124.79, 123.27, 123.15, 121.74, 120.09, 64.01, 58.19, 44.39, 42.94, 40.99, 38.11, 32.00, 31.55, 26.46, 21.25. HPLC-PDA-MS: RT = 5.26 min, 96% (254 nm), PDA λ_{max} = 324 nm, MS (m/z) $[M+H]^+$ 474.15.

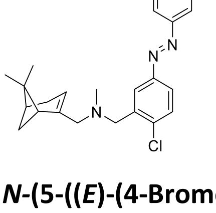

N-(5-((E)-(4-Bromophenyl)diazenyl)-2-chlorobenzyl)-1-((1R,5S)-6,6-dimethylbicyclo[3.1.1]hept-2-en-2-yl)-N-methylmethanamine (15c): The general synthetic procedure C was used with 4-

bromoaniline (109 µL, 1.00 mmol) in DCM (2.7 mL) and Oxone® (1.26 g, 2.1 mmol) in water (10.7 mL), to form the corresponding nitrosocompound **11l**, and the aniline **10c** (153 mg, 0.50 mmol) in DCM (5.5 mL) to give the azocompound **15c** as an orange oil (165 mg, 70%). ^{1}H NMR (600 MHz, $CDCl_3$) δ 8.10 (s, 1H), 7.80 (d, J = 8.4 Hz, 2H), 7.72 (dd, J = 8.6, 2.4 Hz, 1H), 7.65 (d, J = 8.3 Hz, 2H), 7.46 (d, J = 8.5 Hz, 1H), 5.46 (br, 1H), 3.69 (d, J = 14.6 Hz, 1H), 3.60 (d, J = 14.7 Hz, 1H), 3.02 (d, J = 13.2 Hz, 1H), 2.95 (d, J = 13.2 Hz, 1H), 2.40 (dt, J = 8.5, 5.6 Hz, 1H), 2.35 (t, J = 5.7 Hz, 1H), 2.31 (app. d, J = 17.8 Hz, 1H), 2.28 – 2.21 (m, 4H), 2.14 – 2.05 (m, 1H), 1.29 (s, 3H), 1.15 (d, J = 8.6 Hz, 1H), 0.83 (s, 3H). ^{13}C NMR (151 MHz, $CDCl_3$) δ 151.42, 151.15, 146.38, 138.70, 137.04, 132.49, 130.16, 125.66, 125.63, 124.53, 121.65, 120.07, 64.01, 58.22, 44.45, 42.93, 41.04, 38.11, 32.01, 31.57, 26.47, 21.25. HPLC-PDA-MS: RT = 5.96 min, % (254 nm), PDA λ_{max} = 332 nm, MS (m/z) $[M+H]^+$ 474.20.

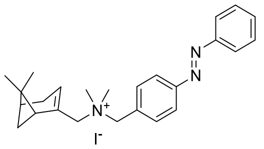

1-((1*R*,5*S*)-6,6-Dimethylbicyclo[3.1.1]hept-2-en-2-yl)-*N*,*N*-dimethyl-*N*-(4-((*E*)-phenyldiazenyl)benzyl)methanaminium iodide (2a): The general synthetic procedure D was used with the amine **12a** (35 mg, 0.10 mmol) and MeI (122 µL, 1.9 mmol) in DCM (2.5 mL) to give the ammonium salt **2a** as an orange solid (32 mg, 66%). ^{1}H NMR (500 MHz, $CDCl_3$) δ 8.03 – 7.76 (m, 6H), 7.59 – 7.38 (m, 3H), 6.23 (br, 1H), 5.27 (s, 2H), 4.42 (d, J = 12.4 Hz, 1H), 4.26 (d, J = 12.4 Hz, 1H), 3.19 (s, 3H), 3.15 (s, 3H), 2.57 – 2.28 (m, 4H), 2.19 – 2.08 (m, 1H), 1.28 (s, 3H), 1.14 (d, J = 8.3 Hz, 1H), 0.83 (s, 3H). ^{13}C NMR (126 MHz, $CDCl_3$) δ 153.72, 152.43, 136.94, 136.09, 134.47, 131.82, 129.63, 129.25, 123.34, 123.25, 68.98, 66.71, 49.13, 48.91, 47.02, 39.70, 38.18, 32.24, 32.06, 26.00, 21.52. HPLC-PDA-MS: RT = 4.54 min, 99.6% (254 nm), PDA λ_{max} = 320, 442 nm, MS (m/z) $[M]^+$ 374.30. HRMS (m/z): $[M]^+$ calculated for $C_{25}H_{32}N_3$, 374.2591; found, 374.2585.

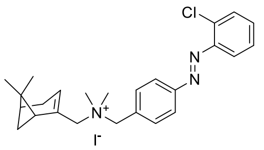

***N*-(4-((*E*)-(2-Chlorophenyl)diazenyl)benzyl)-1-((1*R*,5*S*)-6,6-dimethylbicyclo[3.1.1]hept-2-en-2-yl)-*N*,*N*-dimethylmethanaminium iodide (2b)**: The general synthetic procedure D was used with the amine **12b** (54 mg, 0.14 mmol) and MeI (171 µL, 2.7 mmol) in DCM (2.5 mL) to give the ammonium salt **2b** as an orange solid (62 mg, 84%). ^{1}H NMR (500 MHz, $CDCl_3$) δ 7.92 (d, J = 8.2 Hz, 2H), 7.88 (d, J = 8.2 Hz, 2H), 7.62 (dd, J = 8.1, 1.5 Hz, 1H), 7.52 (dd, J = 8.0, 1.2 Hz, 1H), 7.38 (td, J = 7.7, 1.6 Hz, 1H), 7.29 (t, J = 8.1 Hz, 1H), 6.22 (br, 1H), 5.30 (s, 2H), 4.41 (d, J = 12.4 Hz, 1H), 4.25 (d, J = 12.4 Hz, 1H), 3.19 (s, 3H), 3.15 (s, 3H), 2.49 (dt, J = 9.0, 5.6 Hz, 1H), 2.45 – 2.28 (m, 3H), 2.21 – 2.08 (m, 1H), 1.27 (s, 3H), 1.14 (d, J = 8.9 Hz, 1H), 0.83 (s, 3H). ^{13}C NMR (126 MHz, $CDCl_3$) δ 153.72, 148.37, 136.94, 136.13, 136.06, 134.55, 132.50, 130.87, 130.23, 127.37, 123.78, 117.49, 68.97, 66.59, 49.13, 48.91, 47.01, 39.69, 38.17, 32.24, 32.04, 25.98, 21.50. HPLC-PDA-MS: RT = 4.66 min, 98.7% (254 nm), PDA λ_{max} = 323, 455 nm, MS (m/z) $[M]^+$ 408.20. HRMS (m/z): $[M]^+$ calculated for $C_{25}H_{31}ClN_3$, 408.2201; found, 408.2202.

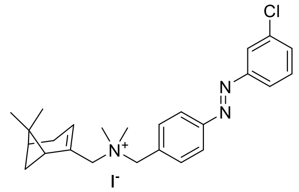

***N*-(4-((*E*)-(3-Chlorophenyl)diazenyl)benzyl)-1-((1*R*,5S)-6,6-dimethylbicyclo[3.1.1]hept-2-en-2-yl)-*N*,*N*-dimethylmethanaminium iodide (2c)**: The general synthetic procedure D was used with the amine **12c** (38 mg, 0.10 mmol) and MeI (121 µL, 1.9 mmol) in DCM (2.5 mL) to give the ammonium salt **2c** as an orange solid (47 mg, 91%). ^{1}H NMR (500 MHz, $CDCl_3$) δ 7.86 (s, 4H), 7.81 (t, J = 2.1 Hz, 1H), 7.77 (dt, J = 6.4, 2.1 Hz, 1H), 7.49 – 7.36 (m, 2H), 6.22 (br, 1H), 5.29 (s, 2H), 4.41 (d, J = 12.4 Hz, 1H), 4.25 (d, J = 12.4 Hz, 1H), 3.18 (s, 3H), 3.15 (s, 3H), 2.50 (dt, J = 9.0, 5.6 Hz, 1H), 2.47 – 2.29 (m, 3H), 2.18 – 2.10 (m, 1H), 1.28 (s, 3H), 1.15 (d, J = 9.0 Hz, 1H), 0.83 (s, 3H). ^{13}C NMR (126 MHz, $CDCl_3$) δ 153.29, 153.10, 137.00, 136.06, 135.30, 134.55, 131.51, 130.30, 130.17, 123.51, 122.84, 121.98, 69.08, 66.61, 49.14, 48.90, 47.04, 39.71, 38.20, 32.26, 32.07, 26.00, 21.52. HPLC-PDA-MS: RT = 4.79 min, 99.5% (254 nm), PDA λ_{max} = 319, 447 nm, MS (m/z) $[M]^+$ 408.25. HRMS (m/z): $[M]^+$ calculated for $C_{25}H_{31}ClN_3$, 408.2201; found, 408.2198.

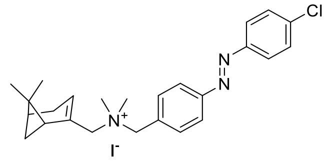

***N*-(4-((*E*)-(4-Chlorophenyl)diazenyl)benzyl)-1-((1*R*,5*S*)-6,6-dimethylbicyclo[3.1.1]hept-2-en-2-yl)-*N*,*N*-dimethylmethanaminium iodide (2d)**: The general synthetic procedure D was used with the amine **12d** (90 mg, 0.23 mmol) and MeI (286 µL, 4.6 mmol) in DCM (4.5 mL) to give the ammonium salt **2d** as an orange solid (104 mg, 85%). ^{1}H NMR (500 MHz, $CDCl_3$) δ 7.91 – 7.81 (m, 4H), 7.78 (d, J = 8.6 Hz, 2H), 7.42 (d, J = 8.6 Hz, 2H), 6.22 (br, 1H), 5.31 (s, 2H), 4.40 (d, J = 12.4 Hz, 1H), 4.24 (d, J = 12.4 Hz, 1H), 3.18 (s, 3H), 3.14 (s, 3H), 2.49 (dt, J = 8.7, 5.7 Hz, 1H), 2.46 – 2.27 (m, 3H), 2.20 – 2.09 (m, 1H), 1.28 (s, 3H), 1.14 (d, J = 9.0 Hz, 1H), 0.83 (s, 3H). ^{13}C NMR (126 MHz, $CDCl_3$) δ 153.39, 150.67, 137.85, 136.94, 136.06, 134.52, 129.92, 129.50, 124.46, 123.38, 68.98, 66.57, 49.11, 48.88, 47.03, 39.70, 38.18, 32.25, 32.06, 26.00, 21.51. HPLC-PDA-MS: RT = 4.80 min, 99.7% (254 nm), PDA λ_{max} = 327, 445 nm, MS (m/z) $[M]^+$ 408.20. HRMS (m/z): $[M]^+$ calculated for $C_{25}H_{31}ClN_3$, 408.2201; found, 408.2193.

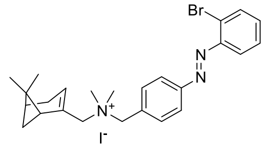

***N*-(4-((*E*)-(2-Bromophenyl)diazenyl)benzyl)-1-((1*R*,5*S*)-6,6-dimethylbicyclo[3.1.1]hept-2-en-2-yl)-*N*,*N*-dimethylmethanaminium iodide (2e)**: The general synthetic procedure D was used with the amine **12e** (143 mg, 0.33 mmol) and MeI (408 µL, 6.5 mmol) in DCM (6.0 mL) to give the ammonium salt **2e** as an orange solid (147 mg, 78%). ^{1}H NMR (500 MHz, $CDCl_3$) δ 7.93 (d, J = 8.4 Hz, 2H), 7.88 (d, J = 8.4 Hz, 2H), 7.71 (dd, J = 7.5, 1.8 Hz, 1H), 7.60 (dd, J = 7.6, 2.1 Hz, 1H), 7.35 – 7.27 (m, 2H), 6.22 (br, 1H), 5.30 (s, 2H), 4.41 (d, J = 12.4 Hz, 1H), 4.24 (d, J = 12.4 Hz, 1H), 3.19 (s, 3H), 3.15 (s, 3H), 2.49 (dt, J = 8.9, 5.6 Hz, 1H), 2.46 – 2.28 (m, 3H), 2.13 (td, J = 5.7, 2.6 Hz, 1H), 1.27 (s, 3H), 1.14 (d, J = 9.0 Hz, 1H), 0.83 (s, 3H). ^{13}C NMR (126 MHz, $CDCl_3$) δ 153.55, 149.25, 136.94, 136.06, 134.56, 133.94, 132.70, 130.25, 128.07, 126.69, 123.87, 117.69, 68.98, 66.59, 49.14, 48.91, 47.01, 39.69, 38.17, 32.24, 32.05,

25.99, 21.51. HPLC-PDA-MS: RT = 4.75 min, 98.8% (254 nm), PDA λ_{max} = 323, 457 nm, MS (m/z) $[M]^+$ 452.25, 454.25. HRMS (m/z): $[M]^+$ calculated for $C_{25}H_{31}BrN_3$, 452. 1696; found, 452.1687.

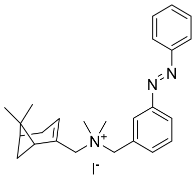

1-((1*R*,5*S*)-6,6-Dimethylbicyclo[3.1.1]hept-2-en-2-yl)-*N*,*N*-dimethyl-N-(3-((*E*)-phenyldiazenyl) benzyl)methanaminium iodide (3a): The general synthetic procedure D was used with the amine **13a** (39 mg, 0.11 mmol) and MeI (136 µL, 2.2 mmol) in DCM (2.5 mL) to give the ammonium salt **3a** as an orange solid (33 mg, 61%). ^{1}H NMR (500 MHz, $CDCl_3$) δ 8.05 (s, 1H), 7.95 (t, J = 6.4 Hz, 2H), 7.90 (d, J = 7.8 Hz, 2H), 7.57 (t, J = 7.8 Hz, 1H), 7.55 – 7.45 (m, 3H), 6.25 (br, 1H), 5.24 (s, 2H), 4.47 (d, J = 12.4 Hz, 1H), 4.32 (d, J = 12.3 Hz, 1H), 3.21 (s, 3H), 3.18 (s, 3H), 2.49 (dt, J = 8.3, 5.6 Hz, 1H), 2.46 – 2.28 (m, 3H), 2.19 – 2.09 (m, 1H), 1.28 (s, 3H), 1.14 (d, J = 8.9 Hz, 1H), 0.83 (s, 3H). ^{13}C NMR (126 MHz, $CDCl_3$) δ 152.79, 152.33, 137.01, 136.09, 135.92, 131.80, 130.33, 129.29, 128.42, 127.97, 124.11, 123.24, 69.13, 66.89, 49.25, 49.02, 47.01, 39.70, 38.17, 32.24, 32.08, 26.00, 21.54. HPLC-PDA-MS: RT = 4.62 min, 99.6% (254 nm), PDA λ_{max} = 317, 440 nm, MS (m/z) $[M]^+$ 374.20. HRMS (m/z): $[M]^+$ calculated for $C_{25}H_{32}N_3$, 374.2591; found, 374.2581.

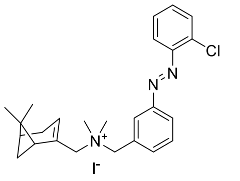

***N*-(3-((*E*)-(2-Chlorophenyl)diazenyl)benzyl)-1-((1*R*,5*S*)-6,6-dimethylbicyclo[3.1.1]hept-2-en-2-yl)-*N*,*N*-dimethylmethanaminium iodide (3b)**: The general synthetic procedure D was used with the amine **13b** (61 mg, 0.16 mmol) and MeI (194 µL, 3.1 mmol) in DCM (4.5 mL) to give the ammonium salt **3b** as an orange solid (34 mg, 41%). ^{1}H NMR (500 MHz, $CDCl_3$) δ 8.09 (t, J = 1.9 Hz, 1H), 7.99 (t, J = 8.2 Hz, 2H), 7.69 (dd, J = 8.0, 1.7 Hz, 1H), 7.60 (t, J = 7.8 Hz, 1H), 7.55 (dd, J = 8.0, 1.3 Hz, 1H), 7.41 (td, J = 7.6, 1.7 Hz, 1H), 7.34 (td, J = 7.7, 1.4 Hz, 1H), 6.27 (br, 1H), 5.24 (s, 2H), 4.46 (d, J = 12.4 Hz, 1H), 4.30 (d, J = 12.4 Hz, 1H), 3.22 (s, 3H), 3.19 (s, 3H), 2.51 (dt, J = 9.0, 5.6 Hz, 1H), 2.47 – 2.31 (m, 3H), 2.19 – 2.10 (m, 1H), 1.29 (s, 3H), 1.16 (d, J = 9.0 Hz, 1H), 0.85 (s, 3H). ^{13}C NMR (126 MHz, $CDCl_3$) δ 152.94, 148.33, 137.16, 136.48, 136.06, 136.05, 132.59, 130.93, 130.52, 129.03, 128.49, 127.53, 123.92, 117.67, 69.28, 66.85, 47.08, 39.73, 32.28, 32.11, 26.02, 21.56. HPLC-PDA-MS: RT = 4.67 min, 98.6% (254 nm), PDA λ_{max} = 321, 452 nm, MS (m/z) $[M]^+$ 408.25. HRMS (m/z): $[M]^+$ calculated for $C_{25}H_{31}ClN_3$, 408.2201; found, 408.2202.

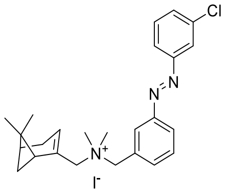

***N*-(3-((*E*)-(3-Chlorophenyl)diazenyl)benzyl)-1-((1*R*,5*S*)-6,6-dimethylbicyclo[3.1.1]hept-2-en-2-yl)-*N*,*N*-dimethylmethanaminium iodide (3c)**: The general synthetic procedure D was used with the amine **13c** (52 mg, 0.13 mmol) and MeI (165 µL, 2.64 mmol) in DCM (2.5 mL) to give the ammonium salt **3c** as an orange solid (33 mg, 47%). ^{1}H NMR (500 MHz, $CDCl_3$) δ 8.06 (s, 1H), 7.95 (t, J = 8.9 Hz, 2H),

7.86 (s, 1H), 7.82 (t, J = 4.3 Hz, 1H), 7.57 (t, J = 7.8 Hz, 1H), 7.44 (d, J = 4.6 Hz, 2H), 6.25 (br, 1H), 5.28 (s, 2H), 4.46 (d, J = 12.3 Hz, 1H), 4.31 (d, J = 12.4 Hz, 1H), 3.21 (s, 3H), 3.19 (s, 3H), 2.50 (dt, J = 8.8, 5.8 Hz, 1H), 2.45 – 2.29 (m, 3H), 2.18 – 2.11 (m, 1H), 1.28 (s, 3H), 1.15 (d, J = 8.9 Hz, 1H), 0.83 (s, 3H). ^{13}C NMR (126 MHz, $CDCl_3$) δ 153.05, 152.45, 137.05, 136.40, 136.07, 135.29, 131.47, 130.38, 130.36, 128.56, 128.15, 124.31, 122.85, 122.04, 69.13, 66.77, 49.23, 49.00, 47.02, 39.70, 38.18, 32.25, 32.09, 26.00, 21.55. HPLC-PDA-MS: RT = 4.79 min, 99.5% (254 nm), PDA λ_{max} = 319, 447 nm, MS (m/z) $[M]^+$ 408.25. HRMS (m/z): $[M]^+$ calculated for $C_{25}H_{31}ClN_3$, 408.2201; found, 408.2191.

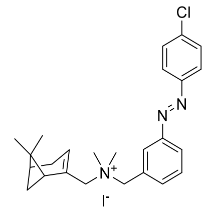

N-(3-((E)-(4-Chlorophenyl)diazenyl)benzyl)-1-((1R,5S)-6,6-dimethylbicyclo[3.1.1]hept-2-en-2-yl)-N,N-dimethylmethanaminium iodide (3d): The general synthetic procedure D was used with the amine **13d** (52 mg, 0.13 mmol) and MeI (165 µL, 2.6 mmol) in DCM (2.5 mL) to give the ammonium salt **3d** as an orange solid (59 mg, 83%). ^{1}H NMR (600 MHz, $CDCl_3$) δ 8.06 (s, 1H), 7.92 (d, J = 8.2 Hz, 2H), 7.84 (d, J = 8.4 Hz, 2H), 7.55 (t, J = 7.8 Hz, 1H), 7.45 (d, J = 8.4 Hz, 2H), 6.24 (br, 1H), 5.28 (s, 2H), 4.45 (d, J = 12.3 Hz, 1H), 4.31 (d, J = 12.4 Hz, 1H), 3.20 (s, 3H), 3.18 (s, 3H), 2.49 (dt, J = 8.8, 5.6 Hz, 1H), 2.45 – 2.25 (m, 3H), 2.19 – 2.09 (m, 1H), 1.27 (s, 3H), 1.14 (d, J = 9.0 Hz, 1H), 0.83 (s, 3H). ^{13}C NMR (126 MHz, $CDCl_3$) δ 152.57, 150.62, 137.76, 137.00, 136.11, 136.07, 130.31, 129.53, 128.52, 128.16, 124.52, 124.11, 69.06, 66.80, 49.20, 48.97, 47.00, 39.69, 38.17, 32.23, 32.07, 26.00, 21.53. HPLC-PDA-MS: RT = 4.78 min, 98.9% (254 nm), PDA λ_{max} = 317, 441 nm, MS (m/z) $[M]^+$ 408.25. HRMS (m/z): $[M]^+$ calculated for $C_{25}H_{31}ClN_3$, 408.2201; found, 408.2186.

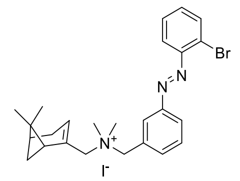

N-(3-((*E*)-(2-Bromophenyl)diazenyl)benzyl)-1-((1*R*,5*S*)-6,6-dimethylbicyclo[3.1.1]hept-2-en-2-yl)-N,N-dimethylmethanaminium iodide (3e): The synthesis and characterization of this compound have been published by us [1].

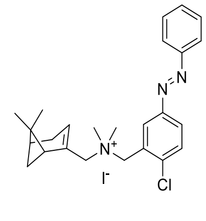

N-(2-Chloro-5-((*E*)-phenyldiazenyl)benzyl)-1-((1*R*,5*S*)-6,6-dimethylbicyclo[3.1.1]hept-2-en-2-yl)-N,N-dimethylmethanaminium iodide (4a): The general synthetic procedure D was used with the amine **14a** (100 mg, 0.25 mmol) and MeI (317 µL, 5.1 mmol) in DCM (5.0 mL) to give the ammonium salt **4a** as an orange solid (100 mg, 74%). ^{1}H NMR (500 MHz, $CDCl_3$) δ 8.46 (s, 1H), 7.98 – 7.89 (m, 3H), 7.61 (d, J = 8.5 Hz, 1H), 7.54 – 7.46 (m, 3H), 6.37 (br, 1H), 5.25 (d, J = 13.4 Hz, 1H), 5.19 (d, J = 13.1 Hz, 1H), 4.66 (d, J = 12.4 Hz, 1H), 4.44 (d, J = 12.3 Hz, 1H), 3.25 (s, 6H), 2.62 – 2.51 (m, 1H), 2.51 – 2.32 (m, 3H), 2.21 – 2.11 (m, 1H), 1.31 (s, 3H), 1.23 (d, J = 8.9 Hz, 1H). ^{13}C NMR (126 MHz, $CDCl_3$) δ 152.17, 151.35, 138.67, 137.66, 136.07, 132.16, 131.87, 131.66, 129.31, 126.48, 124.36, 123.53, 70.40, 63.11, 49.74, 49.52, 47.16, 39.72, 38.26, 32.34, 32.06, 26.01, 21.54. HPLC-PDA-MS: RT = 4.64 min, 99.2% (254 nm), PDA λ_{max} = 231, 324 nm, MS (m/z) $[M]^+$ 408.20. HRMS (m/z): $[M]^+$ calculated for $C_{25}H_{31}ClN_3$, 408.2201; found, 408.2208.

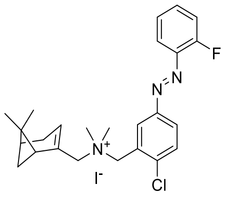

***N*-(2-Chloro-5-((*E*)-(2-fluorophenyl)diazenyl)benzyl)-1-((1*R*,5*S*)-6,6-dimethylbicyclo[3.1.1]hept-2-en-2-yl)-*N,N*-dimethylmethanaminium iodide (4b)**: The general synthetic procedure D was used with the amine **14b** (89 mg, 0.22 mmol) and MeI (270 µL, 4.3 mmol) in DCM (4.3 mL) to give the ammonium salt **4b** as an orange solid (116 mg, 97%). ^{1}H NMR (500 MHz, $CDCl_3$) δ 8.53 (s, 1H), 7.94 (d, J = 8.6 Hz, 1H), 7.79 (t, J = 7.9 Hz, 1H), 7.61 (d, J = 8.6 Hz, 1H), 7.49 (dd, J = 7.1 Hz, 1H), 7.29 – 7.19 (m, 2H), 6.38 (br, 1H), 5.29 (d, J = 13.2 Hz, 1H), 5.23 (d, J = 13.2 Hz, 1H), 4.67 (d, J = 12.4 Hz, 1H), 4.45 (d, J = 12.3 Hz, 1H), 3.27 (s, 6H), 2.61 – 2.52 (m, 1H), 2.52 – 2.32 (m, 3H), 2.23 – 2.12 (m, 1H), 1.32 (s, 3H), 1.24 (d, J = 8.9 Hz, 1H), 0.88 (s, 3H). ^{13}C NMR (126 MHz, $CDCl_3$) δ 161.63, 159.57, 151.52, 140.26, 140.21, 139.20, 137.72, 136.05, 133.92, 133.85, 131.75, 126.57, 124.64, 124.61, 122.82, 118.01, 117.36, 117.20, 70.41, 63.03, 49.77, 47.19, 39.74, 38.28, 32.35, 32.08, 26.02, 21.55. HPLC-PDA-MS: RT = 4.78 min, 99.1% (254 nm), PDA λ_{max} = 328 nm, MS (m/z) $[M]^+$ 426.20. HRMS (m/z): $[M]^+$ calculated for $C_{25}H_{30}FClN_3$, 426.2107; found, 426.2100.

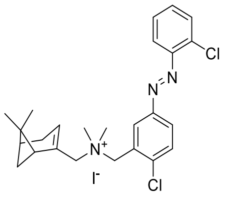

***N*-(2-Chloro-5-((*E*)-(2-chlorophenyl)diazenyl)benzyl)-1-((1*R*,5*S*)-6,6-dimethylbicyclo[3.1.1]hept-2-en-2-yl)-*N,N*-dimethylmethanaminium iodide (4c)**: The general synthetic procedure D was used with the amine **14c** (95 mg, 0.22 mmol) and MeI (277 µL, 4.4 mmol) in DCM (4.5 mL) to give the ammonium salt **4c** as an orange solid (97 mg, 77%). ^{1}H NMR (500 MHz, $CDCl_3$) δ 8.54 (s, 1H), 7.96 (d, J = 8.5 Hz, 1H), 7.74 (d, J = 7.9 Hz, 1H), 7.61 (d, J = 8.6 Hz, 1H), 7.55 (d, J = 7.8 Hz, 1H), 7.43 (t, J = 7.9 Hz, 1H), 7.34 (t, J = 7.4 Hz, 1H), 6.38 (br, 1H), 5.29 (d, J = 13.1 Hz, 1H), 5.23 (d, J = 13.1 Hz, 1H), 4.66 (d, J = 12.4 Hz, 1H), 4.44 (d, J = 12.3 Hz, 1H), 3.27 (s, 6H), 2.60 – 2.51 (m, 1H), 2.51 – 2.33 (m, 3H), 2.24 – 2.12 (m, 1H), 1.32 (s, 3H), 1.24 (d, J = 8.8 Hz, 1H), 0.88 (s, 3H). ^{13}C NMR (126 MHz, $CDCl_3$) δ 151.46, 148.13, 139.33, 137.73, 136.38, 136.05, 133.91, 132.97, 130.93, 127.59, 126.60, 123.11, 117.87, 70.39, 63.02, 49.76, 49.54, 47.18, 39.74, 38.28, 32.35, 32.08, 26.02, 21.56. HPLC-PDA-MS: RT = 4.95 min, 99.5% (254 nm), PDA λ_{max} = 233, 329 nm, MS (m/z) $[M+H]^+$ 442.20. HRMS (m/z): $[M+H]^+$ calculated for $C_{25}H_{30}Cl_2N_3$, 442.1811; found, 4421805.

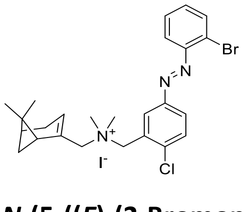

***N*-(5-((*E*)-(2-Bromophenyl)diazenyl)-2-chlorobenzyl)-1-((1*R*,5*S*)-6,6-dimethylbicyclo[3.1.1]hept-2-en-2-yl)-*N,N*-dimethylmethanaminium iodide (4d, VUF15888)**: The synthesis and characterization of this compound have been published by us [1].

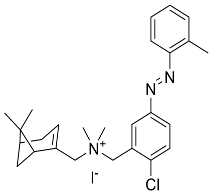

***N*-(2-Chloro-5-((*E*)o-tolyldiazenyl)benzyl)-1-((1*R*,5*S*)-6,6-dimethylbicyclo[3.1.1]hept-2-en-2-yl)-*N*,*N*-dimethylmethanaminium iodide (4f)**: The general synthetic procedure D was used with the amine **14f** (40 mg, 0.10 mmol) and MeI (123 µL, 2.0 mmol) in DCM (2.0 mL) to give the ammonium salt **4f** as an orange solid (22 mg, 41%). ^{1}H NMR (500 MHz, $CDCl_3$) δ 8.46 (s, 1H), 7.92 (d, J = 8.2 Hz, 1H), 7.65 (d, J = 8.1 Hz, 1H), 7.61 (d, J = 8.5 Hz, 1H), 7.38 (t, J = 7.4 Hz, 1H), 7.33 (d, J = 7.7 Hz, 1H), 7.26 – 7.22 (m, 1H), 6.38 (br, 1H), 5.27 (d, J = 12.5 Hz, 1H), 5.21 (d, J = 13.5 Hz, 1H), 4.68 (d, J = 12.4 Hz, 1H), 4.46 (d, J = 12.4 Hz, 1H), 3.26 (s, 6H), 2.74 (s, 3H), 2.61 – 2.52 (m, 1H), 2.52 – 2.33 (m, 3H), 2.23 – 2.11 (m, 1H), 1.31 (s, 3H), 1.24 (d, J = 8.8 Hz, 1H), 0.87 (s, 3H). ^{13}C NMR (126 MHz, $CDCl_3$) δ 151.75, 150.25, 139.52, 138.41, 137.69, 136.09, 132.24, 131.76, 131.62, 131.58, 126.60, 126.45, 124.54, 115.61, 70.35, 63.05, 49.70, 49.47, 47.18, 39.74, 38.28, 32.35, 32.08, 26.03, 21.56, 18.04. HPLC-PDA-MS: RT = 4.99 min, 99.5% (254 nm), PDA λ_{max} = 233, 331 nm, MS (m/z) $[M]^+$ 422.20. HRMS (m/z): $[M]^+$ calculated for $C_{26}H_{33}ClN_3$, 422.2358; found, 422.2359.

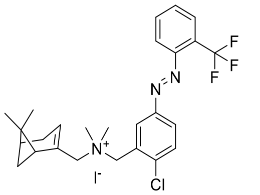

***N*-(2-Chloro-5-((*E*)-(2-(trifluoromethyl)phenyl)diazenyl)benzyl)-1-((1*R*,5*S*)-6,6-dimethylbicyclo [3.1.1]hept-2-en-2-yl)-*N*,*N*-dimethylmethanaminium iodide (4g)**: The general synthetic procedure D was used with the amine **14g** (95 mg, 0.21 mmol) and MeI (257 µL, 4.1 mmol) in DCM (4.0 mL) to give the ammonium salt **4g** as an orange solid (109 mg, 88%). ^{1}H NMR (500 MHz, $CDCl_3$) δ 8.59 (s, 1H), 7.94 (d, J = 8.6 Hz, 1H), 7.89 (d, J = 8.1 Hz, 1H), 7.84 (d, J = 7.8 Hz, 1H), 7.67 (t, J = 7.7 Hz, 1H), 7.65 – 7.55 (m, 2H), 6.39 (br, 1H), 5.31 (d, J = 13.2 Hz, 1H), 5.26 (d, J = 13.2 Hz, 1H), 4.68 (d, J = 12.3 Hz, 1H), 4.46 (d, J = 12.3 Hz, 1H), 3.27 (s, 6H), 2.63 – 2.52 (m, 1H), 2.52 – 2.35 (m, 3H), 2.23 – 2.14 (m, 1H), 1.32 (s, 3H), 1.24 (d, J = 8.9 Hz, 1H), 0.88 (s, 3H). ^{13}C NMR (126 MHz, $CDCl_3$) δ 151.33, 148.75, 139.69, 137.78, 136.04, 134.59, 132.92, 131.91, 131.69, 129.54, 129.29, 129.05, 128.80, 127.29, 126.84, 126.80, 126.75, 126.71, 126.64, 125.11, 122.94, 122.71, 120.76, 116.50, 70.39, 63.01, 49.75, 49.52, 47.19, 39.74, 38.29, 32.36, 32.08, 26.02, 21.54. HPLC-PDA-MS: RT = 4.88 min, 99.7% (254 nm), PDA λ_{max} = 324, nm, MS (m/z) $[M]^+$ 476.25. HRMS (m/z): $[M]^+$ calculated for $C_{26}H_{30}ClF_3N_3$ 476.2075; found, 476.2092.

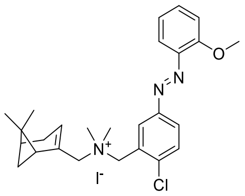

***N*-(2-Chloro -5-((*E*)-(2-methoxyphenyl)diazenyl)benzyl)-1-((1*R*,5*S*)-6,6-dimethylbicyclo[3.1.1]hept-2-en-2-yl)-*N*,*N*-dimethylmethanaminium iodide (4h)**: The general synthetic procedure D was used with amine **14h** (70 mg, 0.17 mmol) and MeI (206 µL, 3.3 mmol) in DCM (3.3 mL). Following the general synthetic procedure D the ammonium salt **4h** did not precipitate as a solid, but as an oil. MTBE was

decanted and the oil was washed with several portions of MTBE. The product was dried in the vacuum oven to give an orange oil, which corresponded to the expected product **4h** with the presence of a 0.44 molar fraction of solvated MTBE (91 mg, 91%). The MTBE solvate could not be removed under high vacuum and temperature and was taken into account when calculating the yield and the concentrations for pharmacological experiments. The product contained 95% of the *trans*-isomer and 5% of *cis*-isomer. ^{1}H NMR (600 MHz, $CDCl_3$) δ 8.41 (d, J = 2.3 Hz, 1H), 7.91 (dd, J = 8.6, 2.3 Hz, 1H), 7.71 (dd, J = 8.1, 1.7 Hz, 1H), 7.60 (d, J = 8.6 Hz, 1H), 7.48 (ddd, J = 8.6, 7.2, 1.7 Hz, 1H), 7.10 (d, J = 8.3 Hz, 1H), 7.01 (t, J = 7.6 Hz, 1H), 6.38 (br, J = 3.4 Hz, 1H), 5.23 (d, J = 13.3 Hz, 1H), 5.17 (d, J = 13.3 Hz, 1H), 4.67 (d, J = 12.4 Hz, 1H), 4.42 (d, J = 12.4 Hz, 1H), 4.03 (s, 3H), 3.25 (s, 6H), 2.55 (dt, J = 8.9, 5.6 Hz, 1H), 2.52 – 2.35 (m, 3H), 2.24 – 2.13 (m, 1H), 1.32 (s, 3H), 0.88 (s, 3H). ^{13}C NMR (151 MHz, $CDCl_3$) δ 157.73, 152.12, 141.84, 138.29, 137.73, 136.07, 133.98, 132.69, 131.67, 126.32, 123.61, 120.99, 117.31, 113.04, 72.91, 70.49, 63.38, 56.54, 49.77, 49.59, 49.55, 47.27, 39.80, 38.31, 32.38, 32.09, 27.11, 26.05, 21.57. HPLC-PDA-MS: *trans-isomer*: RT = 4.48 min, 94.7% (254 nm), PDA λ_{max} = 323, 362 nm, MS (m/z) $[M]^+$ 438.30; *cis-isomer*: RT = 4.20 min, 4.1% (254 nm), PDA λ_{max} = 434 nm, MS (m/z) $[M]^+$ 438.35. HRMS (m/z): $[M]^+$ calculated for $C_{26}H_{33}ClN_3O$, 438.2307; found, 438.2298.

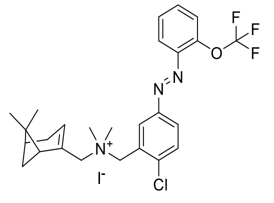

***N*-(2-Chloro-5-((*E*)-(2-(trifluoromethoxy)phenyl)diazenyl)benzyl)-1-((1*R*,5*S*)-6,6-dimethylbicyclo [3.1.1]hept-2-en-2-yl)-*N*,*N*-dimethylmethanaminium iodide (4i)**: The general synthetic procedure D was used with the amine **14i** (74 mg, 0.16 mmol) and MeI (194 µL, 3.1 mmol) in DCM (3.10mL) to give the ammonium salt **4i** as an orange solid (72 mg, 75%). ^{1}H NMR (500 MHz, $CDCl_3$) δ 8.56 (s, 1H), 7.92 (d, J = 8.4 Hz, 1H), 7.83 (d, J = 8.0 Hz, 1H), 7.61 (d, J = 8.8 Hz, 1H), 7.55 (t, J = 7.8 Hz, 1H), 7.46 – 7.35 (m, 2H), 6.39 (br, 1H), 5.30 (d, J = 13.1 Hz, 1H), 5.24 (d, J = 13.3 Hz, 1H), 4.69 (d, J = 12.3 Hz, 1H), 4.48 (d, J = 12.4 Hz, 1H), 3.28 (s, 6H), 2.65 – 2.50 (m, 1H), 2.50 – 2.36 (m, 3H), 2.23 – 2.12 (m, 1H), 1.31 (s, 3H), 1.23 (d, J = 8.9 Hz, 1H), 0.87 (s, 3H). ^{13}C NMR (126 MHz, $CDCl_3$) δ 151.43, 147.37, 144.43, 139.56, 137.72, 136.07, 134.50, 133.26, 131.85, 127.88, 126.62, 123.73, 123.19, 122.35, 121.68, 119.63, 117.75, 117.58, 70.32, 62.96, 49.72, 49.50, 47.15, 39.72, 38.26, 32.34, 32.06, 26.00, 21.53. HPLC-PDA-MS: RT = 5.05 min, 99.7% (254 nm), PDA λ_{max} = 325 nm, MS (m/z) $[M]^+$ 492.25. HRMS (m/z): $[M]^+$ calculated for $C_{26}H_{30}ClF_3N_3O$, 492.2019; found, 492.2024

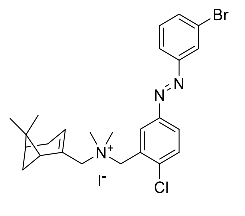

***N*-(5-((*E*)-(3-Bromophenyl)diazenyl)-2-chlorobenzyl)-1-((1*R*,5*S*)-6,6-dimethylbicyclo[3.1.1]hept-2-en-2-yl)-*N*,*N*-dimethylmethanaminium iodide (5b)**: The general synthetic procedure D was used with the amine **15b** (138 mg, 0.29 mmol) and MeI (365 µL, 5.8 mmol) in DCM (6.0 mL) to give the ammonium salt **5b** as an orange solid (148 mg, 82%). ^{1}H NMR (500 MHz, $CDCl_3$) δ 8.50 (s, 1H), 8.02 (s, 1H), 7.97 – 7.87 (m, 2H), 7.65 – 7.56 (m, 2H), 7.39 (t, J = 7.7 Hz, 1H), 6.37 (br, 1H), 5.28 (d, J = 13.1 Hz, 1H), 5.22 (d, J = 13.2 Hz, 1H), 4.66 (d, J = 12.3 Hz, 1H), 4.45 (d, J = 12.4 Hz, 1H), 3.26 (s, 6H), 2.63 – 2.50 (m, 1H), 2.50 – 2.31 (m, 3H), 2.22 – 2.10 (m, 1H), 1.31 (s, 3H), 1.23 (d, J = 8.8 Hz, 1H), 0.86 (s, 3H). ^{13}C

NMR (126 MHz, $CDCl_3$) δ 152.95, 150.97, 139.28, 137.68, 136.07, 134.69, 131.89, 131.74, 130.72, 126.67, 125.09, 124.67, 123.67, 123.25, 70.40, 62.93, 47.15, 39.72, 38.26, 32.34, 32.07, 26.02, 21.55. HPLC-PDA-MS: RT = 4.96 min, 99.7% (254 nm), PDA λ_{max} = 324 nm, MS (m/z) $[M]^+$ 488.20. HRMS (m/z): $[M]^+$ calculated for $C_{25}H_{30}BrClN_3$, 486.1306; found, 486.1302.

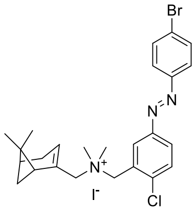

***N*-(5-((*E*)-(4-Bromophenyl)diazenyl)-2-chlorobenzyl)-1-((1*R*,5*S*)-6,6-dimethylbicyclo[3.1.1]hept-2-en-2-yl)-*N*,*N*-dimethylmethanaminium iodide (5c)**: The general synthetic procedure D was used with the amine **15c** (76 mg, 0.16 mmol) and MeI (201 µL, 3.2 mmol) in DCM (3.3 mL) to give the ammonium salt **5c** as an orange solid (58 mg, 59%). ^{1}H NMR (500 MHz, $CDCl_3$) δ 8.51 (s, 1H), 7.93 (d, J = 8.0 Hz, 1H), 7.82 (d, J = 8.7 Hz, 2H), 7.67 – 7.58 (m, 3H), 6.36 (br, 1H), 5.28 (d, J = 12.9 Hz, 1H), 5.21 (d, J = 13.2 Hz, 1H), 4.64 (d, J = 12.4 Hz, 1H), 4.44 (d, J = 12.3 Hz, 1H), 3.25 (s, 6H), 2.61 – 2.50 (m, 1H), 2.50 – 2.31 (m, 3H), 2.17 (app. q, J = 4.3, 3.8 Hz, 1H), 1.30 (s, 3H), 1.23 (d, J = 8.9 Hz, 1H), 0.86 (s, 3H). ^{13}C NMR (126 MHz, $CDCl_3$) δ 151.14, 150.85, 138.99, 137.66, 136.06, 132.57, 131.97, 131.70, 126.75, 126.62, 125.00, 124.48, 70.35, 62.99, 49.77, 49.53, 47.15, 39.72, 38.26, 32.34, 32.06, 26.01, 21.55. HPLC-PDA-MS: RT = 4.95 min, 97.6% (254 nm), PDA λ_{max} = 233, 333 nm, MS (m/z) $[M]^+$ 488.20. HRMS (m/z): $[M]^+$ calculated for $C_{25}H_{30}BrClN_3$, 486.1306; found, 4861304.

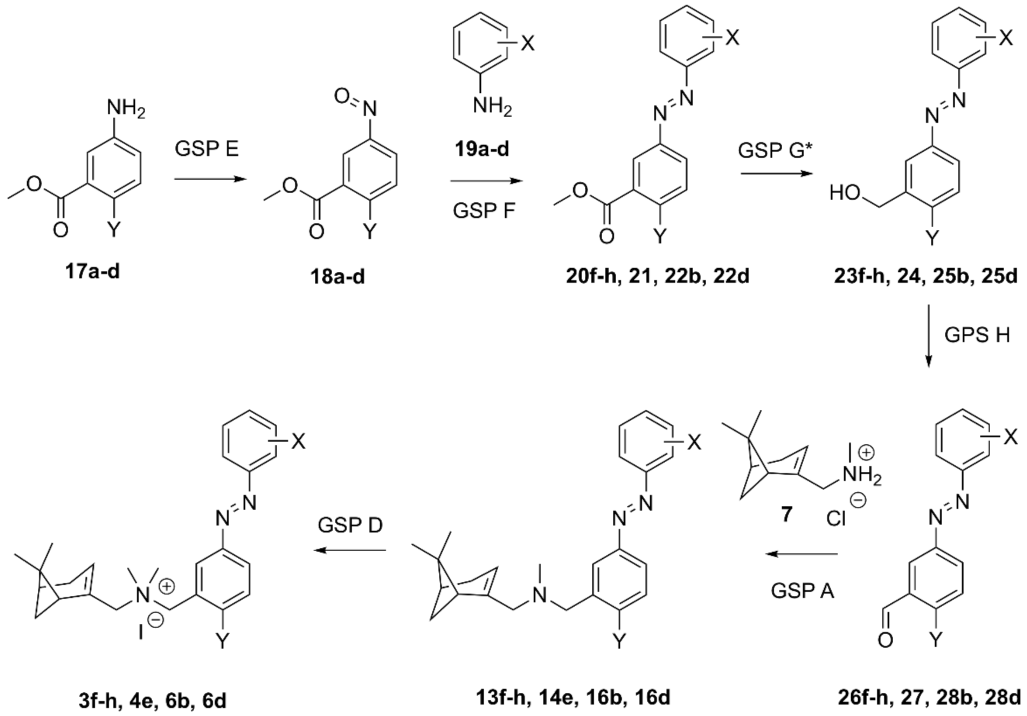

Scheme S2: Synthetic strategies for compounds **3f–h**, **4e**, **6b** and **6d.** (*)Compound **23h** was not prepared under the same conditions of the general procedure G. GSP = General Synthetic Procedure.

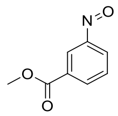

Methyl 3-nitrosobenzoate (18a): The general synthetic procedure E was used with methyl 3-aminobenzoate (**17a**) (1.00 g, 6.6 mmol) in DCM (20 mL) and Oxone® (8.3 g, 13.6 mmol) in water (80 mL) to form the corresponding nitrosocompound **18a** (1.03g, 94%). ^{1}H NMR (500 MHz, $CDCl_3$) δ 8.60 (s, 1H), 8.39 (d, J = 7.8 Hz, 1H), 8.00 (d, J = 7.8 Hz, 1H), 4.00 (s, 6H). ^{13}C NMR (126 MHz, $CDCl_3$) δ 165.84, 164.89, 135.84, 131.83, 129.66, 123.90, 122.61, 52.84.

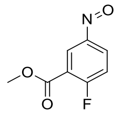

Methyl 2-fluoro-5-nitrosobenzoate (18b): The synthesis and characterization of this compound have been published by us [1].

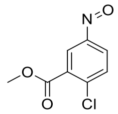

Methyl 2-chloro-5-nitrosobenzoate (18c): The general synthetic procedure E was used with methyl 5-amino-2-chlorobenzoate (**17c**) (630 mg, 3.4 mmol) in DCM (10 mL) and Oxone® (4.38 g, 7.1 mmol) in water (40 mL) to form the corresponding nitrosocompound **18c** (665 mg, 98%). ^{1}H NMR (500 MHz, $CDCl_3$) δ 8.54 (d, J = 2.9 Hz, 1H), 7.78 (dd, J = 8.5, 2.2 Hz, 1H), 7.70 (d, J = 8.5 Hz, 1H), 4.02 (s, 3H). ^{13}C NMR (126 MHz, $CDCl_3$) δ 164.84, 162.46, 141.56, 132.48, 131.26, 125.66, 122.09, 53.15.

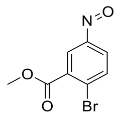

Methyl 2-bromo-5-nitrosobenzoate (18d): The synthesis and characterization of this compound have been published by us [1].

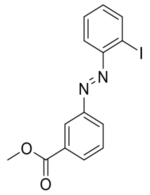

Methyl (*E*)-3-((2-iodophenyl)diazenyl)benzoate (20f): The general synthetic procedure F was used with the nitrosocompound **18a** (444 mg, 2.7 mmol) and 2-iodoaniline **19a** (544 mg, 2.5 mmol) in AcOH (10 mL) to give the azocompound **20f** as an orange solid (662 mg, 73%). ^{1}H NMR (600 MHz, $CDCl_3$) δ 8.64 (t, J = 1.8 Hz, 1H), 8.17 (td, J = 8.3, 7.8, 1.6 Hz, 2H), 8.04 (d, J = 7.9 Hz, 1H), 7.65 (dd, J = 8.0, 1.6 Hz, 1H), 7.61 (t, J = 7.8 Hz, 1H), 7.43 (t, J = 7.6 Hz, 1H), 7.18 (td, J = 7.6, 1.6 Hz, 1H), 3.98 (s, 3H). ^{13}C NMR (151 MHz, $CDCl_3$) δ 166.55, 152.35, 151.25, 140.08, 132.74, 132.36, 131.56, 129.42, 129.06, 126.40, 126.10, 117.47, 103.02, 52.55. HPLC-PDA-MS: RT = 5.77 min, 93% (254 nm), PDA λ_{max} = 321 nm, MS (m/z) $[M+H]^+$ 367.05.

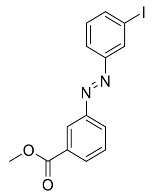

Methyl (*E*)-3-((3-iodophenyl)diazenyl)benzoate (20g): The general synthetic procedure F was used with the nitrosocompound **18a** (280 mg, 1.70 mmol) and 3-iodoaniline **19b** (248 mg, 1.13 mmol) in AcOH (11 mL) to give the azocompound **20g** as an orange solid (397 mg, 96%). ^{1}H NMR (600 MHz, $CDCl_3$) δ 8.55 (t, J = 1.9 Hz, 1H), 8.27 (t, J = 1.8 Hz, 1H), 8.17 (dt, J = 7.7, 1.4 Hz, 1H), 8.09 (ddd, J = 8.0, 2.0, 1.2 Hz, 1H), 7.93 (ddd, J = 7.9, 1.9, 1.0 Hz, 1H), 7.81 (dt, J = 7.9, 1.3 Hz, 1H), 7.60 (t, J = 7.8 Hz, 1H), 7.27 (t, J = 7.9 Hz, 1H), 3.97 (s, 3H). ^{13}C NMR (151 MHz, $CDCl_3$) δ 166.53, 153.26, 152.36, 140.07, 132.26, 131.51, 130.88, 130.83, 129.39, 127.26, 124.25, 123.85, 94.71, 52.53. HPLC-PDA-MS: RT = 6.01 min, 99.4 % (254 nm), PDA λ_{max} = 315 nm, MS (m/z) $[M+H]^+$ 366.95.

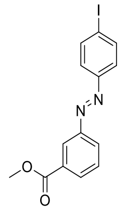

Methyl (*E*)-3-((4-iodophenyl)diazenyl)benzoate (20h): The general synthetic procedure F was used with the nitrosocompound **18a** (450 mg, 2.7 mmol) and 4-iodoaniline **19c** (475 mg, 2.2 mmol) in AcOH (10 mL) to give the azocompound **20h** as an orange solid (730 mg, 92%). ^{1}H NMR (600 MHz, $CDCl_3$) δ 8.56 (s, 1H), 8.16 (d, J = 7.6 Hz, 1H), 8.09 (d, J = 8.0 Hz, 1H), 7.88 (d, J = 8.2 Hz, 2H), 7.68 (d, J = 8.3 Hz, 2H), 7.60 (t, J = 7.8 Hz, 1H), 3.97 (s, 3H). ^{13}C NMR (151 MHz, $CDCl_3$) δ 166.59, 152.52, 151.85, 138.58, 132.12, 131.51, 129.40, 127.19, 124.73, 124.23, 98.42, 52.54. HPLC-PDA-MS: RT = 5.99 min, 97.8 % (254 nm), PDA λ_{max} = 332 nm, MS (m/z) $[M+H]^+$ 367.00.

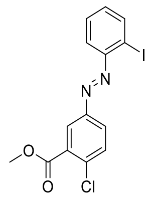

Methyl (*E*)-2-chloro-5-((2-iodophenyl)diazenyl)benzoate (21): The general synthetic procedure F was used with the nitrosocompound **18c** (546 mg, 2.7 mmol) and 2-iodoaniline **19a** (563 mg, 2.6 mmol) in AcOH (10 mL) to give the azocompound **21** as an orange solid (637 mg, 62%). ^{1}H NMR (600 MHz, $CDCl_3$) δ 8.46 (d, J = 2.4 Hz, 1H), 8.04 (d, J = 8.2 Hz, 1H), 8.02 (dd, J = 8.5, 2.2 Hz, 1H), 7.63 (dd, J = 8.0, 1.6 Hz, 1H), 7.60 (d, J = 8.5 Hz, 1H), 7.43 (t, J = 7.6 Hz, 1H), 7.19 (td, J = 7.6, 1.6 Hz, 1H), 3.99 (s, 3H). δ ^{13}C NMR (151 MHz, $CDCl_3$) δ 165.59, 151.07, 150.38, 140.16, 136.78, 132.99, 132.19, 130.97, 129.08, 128.01, 125.61, 103.31, 52.85. HPLC-PDA-MS: RT = 5.92 min, 86% (254 nm), PDA λ_{max} = 329 nm, MS (m/z) $[M+H]^+$ 401.00.

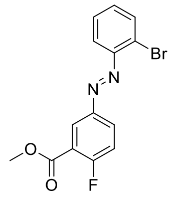

Methyl (*E*)-5-((2-bromophenyl)diazenyl)-2-fluorobenzoate (22b): The synthesis and characterization of this compound have been published by us [1].

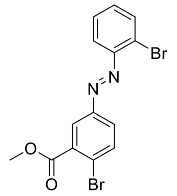

Methyl (*E*)-2-bromo-5-((2-bromophenyl)diazenyl)benzoate (22d): The synthesis and characterization of this compound have been published by us [1].

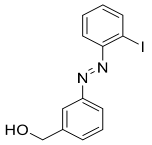

(*E*)-(3-((2-Iodophenyl)diazenyl)phenyl)methanol (23f): The general synthetic procedure G was used with benzoate **20f** (172 mg, 0.40 mmol) and DIBAL-H (1.6 mL, 1.6 mmol) in THF (8 mL) to give the alcohol **23f** as an orange oil (102 mg, 76%). ^{1}H NMR (600 MHz, $CDCl_3$) δ 8.04 (dd, J = 7.9, 1.3 Hz, 1H), 7.99 (br, 1H), 7.96 – 7.88 (m, 1H), 7.63 (dd, J = 8.0, 1.6 Hz, 1H), 7.55 – 7.51 (m, 2H), 7.43 (ddd, J = 8.2, 7.3, 1.3 Hz, 1H), 7.17 (td, J = 7.6, 1.6 Hz, 1H), 4.83 (s, 2H). ^{13}C NMR (151 MHz, $CDCl_3$) δ 152.67, 151.46, 142.29, 140.01, 132.42, 130.10, 129.57, 129.07, 123.01, 121.95, 117.50, 102.65, 65.10. HPLC-PDA-MS: RT = 4.88 min, 97.9% (254 nm), PDA λ_{max} = 324 nm, MS (m/z) $[M+H]^+$ 338.75.

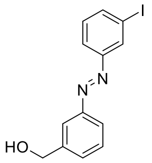

(*E*)-(3-((3-Iodophenyl)diazenyl)phenyl)methanol (23g): The general synthetic procedure G was used with benzoate **22g** (364 mg, 0.99 mmol) and DIBAL-H (3.0 mL, 3.0 mmol) in THF (20 mL) to give the alcohol **23g** as an orange oil (334 mg, 99%). ^{1}H NMR (600 MHz, $CDCl_3$) δ 8.25 (t, J = 1.8 Hz, 1H), 7.94 – 7.88 (m, 2H), 7.85 (dt, J = 6.9, 2.1 Hz, 1H), 7.80 (ddd, J = 7.8, 1.7, 1.1 Hz, 1H), 7.55 – 7.49 (m, 2H), 7.27 (t, J = 7.9 Hz, 1H), 4.82 (s, 2H). ^{13}C NMR (151 MHz, $CDCl_3$) δ 153.47, 152.68, 142.27, 139.77, 130.81, 130.75, 129.96, 129.53, 123.75, 123.01, 120.86, 94.71, 65.03. HPLC-PDA-MS: RT = 5.09 min, 98.8% (254 nm), PDA λ_{max} = 320 nm, MS (m/z) $[M+H]^+$ 339.05.

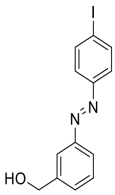

(*E*)-(3-((4-Iodophenyl)diazenyl)phenyl)methanol (23h): Benzoate **20h** (208mg, 0.56 mmol) was dissolved in a mixture of DCM (8 mL) and PhMe (4 mL). DIBAL-H (682 µL, 0.68 mmol, 1.0 M in THF) was added slowly at -78°C and the reaction mixture was stirred for 1 h at -78°C. MeOH (1 mL) was added to quench the reaction. The mixture was slowly warmed to rt. An aqueous Rochelle salt solution (10%, 20 mL) was added and the mixture was stirred for 3 h at rt. Water (30 mL) and DCM (20 mL) were added and the layers were separated. The organic layer was washed with brine (3 x 20 mL), dried over Na_2SO_4, filtered and evaporated *in vacuo* to give a residue (orange/red oil) that showed only 50% conversion. The residue was purified with automated flash chromatography (cHex/EtOAc) to give alcohol **23h** as an orange/red oil with high purity (86 mg, 45%). ^{1}H NMR (500 MHz, $CDCl_3$) δ 7.91 (s, 1H), 7.90 – 7.82 (m, 3H), 7.66 (d, J = 8.4 Hz, 2H), 7.56 – 7.48 (m, 2H), 4.81 (s, 2H). ^{13}C NMR (126 MHz, $CDCl_3$) δ 152.76, 151.97, 142.23, 138.51, 129.82, 129.53, 124.62, 122.95, 120.77, 97.97, 65.04. HPLC-PDA-MS: RT = 5.08 min, 93.4% (254 nm), PDA λ_{max} = 333 nm, MS (m/z) $[M+H]^+$ 338.95.

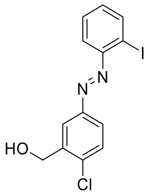

(*E*)-(2-Chloro-5-((2-iodophenyl)diazenyl)phenyl)methanol (24): The general synthetic procedure G was used with benzoate **21** (140 mg, 0.28 mmol) and DIBAL-H (1.3 mL, 1.3 mmol) in THF (6 mL) to give the alcohol **24** as an orange oil (88 mg, 81%). ^{1}H NMR (600 MHz, $CDCl_3$) δ 8.16 (d, J = 2.4 Hz, 1H), 8.04 (dd, J = 7.9, 1.3 Hz, 1H), 7.87 (dd, J = 8.4, 2.4 Hz, 1H), 7.63 (dd, J = 8.0, 1.6 Hz, 1H), 7.52 (d, J = 8.4 Hz, 1H), 7.43 (ddd, J = 8.1, 7.2, 1.3 Hz, 1H), 7.18 (td, J = 7.5, 1.6 Hz, 1H), 4.89 (s, 2H). ^{13}C NMR (151 MHz, $CDCl_3$) δ 151.29, 151.19, 140.09, 139.52, 135.79, 132.65, 130.34, 129.08, 124.10, 123.23, 117.46, 102.96, 62.80. HPLC-PDA-MS: RT = 5.33 min, 99.1% (254 nm), PDA λ_{max} = 331 nm, MS (m/z) $[M+H]^+$ 373.05.

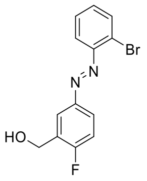

(*E*)-(5-((2-Bromophenyl)diazenyl)-2-fluorophenyl)methanol (25b): The synthesis and characterization of this compound have been published by us [1].

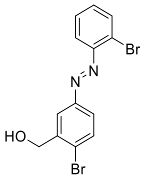

(*E*)-(2-Bromo-5-((2-bromophenyl)diazenyl)phenyl)methanol (25d): The synthesis and characterization of this compound have been published by us [1].

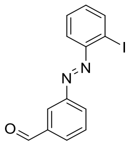

(*E*)-3-((2-Iodophenyl)diazenyl)benzaldehyde (26f): The general synthetic procedure H was used with alcohol **23f** (187 mg, 0.55 mmol) and Dess Martin periodinane (235 mg , 0.55 mmol) in DCM (11 mL) to give benzaldehyde **26f** as an orange oil (171 mg, 92%). ^{1}H NMR (600 MHz, $CDCl_3$) δ 10.15 (s, 1H), 8.46 (t, J = 1.9 Hz, 1H), 8.25 (ddd, J = 7.9, 2.1, 1.2 Hz, 1H), 8.06 (dd, J = 7.9, 1.2 Hz, 1H), 8.04 (dt, J = 7.6, 1.4 Hz, 2H), 7.71 (t, J = 7.7 Hz, 1H), 7.67 (dd, J = 8.1, 1.6 Hz, 1H), 7.45 (td, J = 7.7, 7.3, 1.3 Hz, 1H), 7.21 (td, J = 7.6, 1.6 Hz, 1H). ^{13}C NMR (151 MHz, $CDCl_3$) δ 191.70, 152.74, 151.17, 140.17, 137.56, 132.99, 131.70, 130.12, 129.11, 128.78, 125.41, 117.48, 103.26. HPLC-PDA-MS: RT = 5.49 min, 99.5% (254 nm), PDA λ_{max} = 319 nm, MS (m/z) $[M+H]^+$ 337.10.

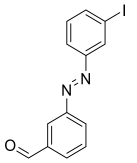

((*E*)-3-((3-Iodophenyl)diazenyl)benzaldehyde (26g): The general synthetic procedure H was used with alcohol **23g** (329 mg, 0.97 mmol) and Dess Martin periodinane (413 mg, 0.97 mmol) in DCM (20 mL) to give benzaldehyde **26g** as an orange oil (222 mg, 68%). ^{1}H NMR (600 MHz, $CDCl_3$) δ 10.14 (s, 1H), 8.40 (t, J = 1.8 Hz, 1H), 8.29 (t, J = 1.8 Hz, 1H), 8.19 (ddd, J = 7.9, 2.0, 1.2 Hz, 1H), 8.03 (dt, J = 7.6, 1.4 Hz, 1H), 7.95 (ddd, J = 7.9, 1.9, 1.0 Hz, 1H), 7.84 (dt, J = 8.0, 1.3 Hz, 1H), 7.71 (t, J = 7.7 Hz, 1H), 7.30 (t, J = 7.9 Hz, 1H). ^{13}C NMR (151 MHz, $CDCl_3$) δ 191.70, 153.21, 152.80, 140.33, 137.52, 131.71, 130.94, 130.91, 130.10, 129.07, 124.07, 123.97, 94.75. HPLC-PDA-MS: RT = 5.67 min, 91.6% (254 nm), PDA λ_{max} = 319 nm, MS (m/z) $[M+H]^+$ 337.00.

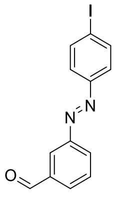

(*E*)-3-((4-Iodophenyl)diazenyl)benzaldehyde (26h): The general synthetic procedure H was used with alcohol **23h** (187 mg, 0.55 mmol) and Dess Martin periodinane (235 mg, 0.55 mmol) in DCM (11 mL) to give benzaldehyde **26h** as an orange oil (156 mg, 84%). ^{1}H NMR (500 MHz, $CDCl_3$) δ 10.14 (s, 1H), 8.40 (s, 1H), 8.19 (d, J = 7.9 Hz, 1H), 8.02 (d, J = 7.5 Hz, 1H), 7.90 (d, J = 8.4 Hz, 2H), 7.70 (t, J = 7.9 Hz, 1H), 7.69 (d, J = 8.4 Hz, 2H). ^{13}C NMR (126 MHz, $CDCl_3$) 191.81, 152.89, 151.74, 138.65, 137.47, 131.62, 130.09, 129.02, 124.78, 123.99, 98.78. HPLC-PDA-MS: RT = 5.65 min, 99.6% (254 nm), PDA λ_{max} = 332 nm, MS (m/z) $[M+H]^+$ 337.00.

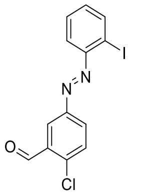

(*E*)-2-Chloro-5-((2-iodophenyl)diazenyl)benzaldehyde (27): The general synthetic procedure H was used with alcohol **24** (200 mg, 0.54 mmol) and Dess Martin periodinane (228 mg, 0.54 mmol) in DCM (11 mL) to give benzaldehyde **27** as an orange oil (192 mg, 97%). ^{1}H NMR (600 MHz, $CDCl_3$) δ 10.55 (s, 1H), 8.53 (d, J = 2.5 Hz, 1H), 8.13 (dd, J = 8.5, 2.5 Hz, 1H), 8.05 (dd, J = 7.9, 1.3 Hz, 1H), 7.66 (dd, J = 8.0, 1.6 Hz, 1H), 7.62 (d, J = 8.5 Hz, 1H), 7.44 (ddd, J = 8.2, 7.3, 1.3 Hz, 1H), 7.21 (td, J = 7.6, 1.6 Hz, 1H). ^{13}C NMR (151 MHz, $CDCl_3$) δ 189.10, 150.90, 140.17, 140.09, 133.22, 133.05, 131.66, 128.99, 127.43, 126.07, 117.32, 103.38. HPLC-PDA-MS: RT = 5.91 min, 97.9% (254 nm), PDA λ_{max} = 321 nm, MS (m/z) $[M+H]^+$ 371.10.

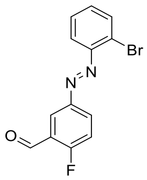

(*E*)-5-((2-Bromophenyl)diazenyl)-2-fluorobenzaldehyde (28b): The synthesis and characterization of this compound have been published by us [1].

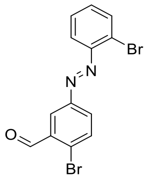

(*E*)-2-Bromo-5-((2-bromophenyl)diazenyl)benzaldehyde (28d): The synthesis and characterization of this compound have been published by us [1].

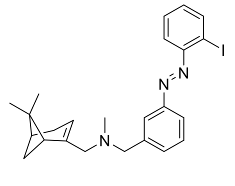

1-((1*R*,5*S*)-6,6-Dimethylbicyclo[3.1.1]hept-2-en-2-yl)-*N*-(3-((*E*)-(2-iodophenyl)diazenyl)benzyl)-*N*-methylmethanamine (13f): The general synthetic procedure A was used with ammonium salt **7** (115 mg, 0.57 mmol), TEA (89 µL, 0.64 mmol) and benzaldehyde **26f** (153 mg, 0.46 mmol) in DCE (9.0 mL) to give the tertiary amine **13f** (213 g, 96%). ^{1}H NMR (600 MHz, $CDCl_3$) δ 8.03 (d, J = 7.8 Hz, 1H), 7.94 (s, 1H), 7.91 – 7.84 (m, 1H), 7.63 (dd, J = 8.0, 1.6 Hz, 1H), 7.51 – 7.45 (m, 2H), 7.43 (td, J = 7.7, 1.2 Hz, 1H), 7.16 (td, J = 7.6, 1.6 Hz, 1H), 5.46 (br, 1H), 3.64 (d, J = 13.4 Hz, 1H), 3.48 (d, J = 13.4 Hz, 1H), 2.99 (d, J = 13.6 Hz, 1H), 2.88 (d, J = 13.0 Hz, 1H), 2.44 (dt, J = 8.6, 5.6 Hz, 1H), 2.36 (t, J = 5.2 Hz, 1H), 2.31 (app. d, J = 17.7 Hz, 1H), 2.24 (app. d, J = 17.5 Hz, 1H), 2.16 (s, 3H), 2.15 – 2.07 (m, 1H), 1.31 (s, 3H), 1.17 (d, J = 8.6 Hz, 1H), 0.85 (s, 3H). ^{13}C NMR (151 MHz, $CDCl_3$) δ 152.53, 151.54, 146.49, 141.29, 139.95, 132.25, 132.21, 129.09, 129.04, 124.55, 121.79, 120.03, 117.49, 102.54, 63.93, 61.39, 44.37, 42.69, 41.11, 38.13, 32.04, 31.57, 26.51, 21.29. HPLC-PDA-MS: RT = 4.81 min, 99.9% (254 nm), PDA λ_{max} = 321 nm, MS (m/z) $[M+H]^+$ 486.25

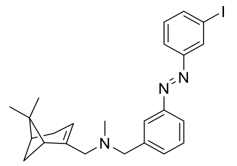

1-((1*R*,5*S*)-6,6-Dimethylbicyclo[3.1.1]hept-2-en-2-yl)-*N*-(3-((*E*)-(3-iodophenyl)diazenyl)benzyl)-*N*-methylmethanamine (13g): The general synthetic procedure A was used with ammonium salt **7** (160 mg, 0.79 mmol), TEA (124 µL, 0.89 mmol) and benzaldehyde **26g** (213 mg, 0.63 mmol) in DCE (12.5 mL) to give the tertiary amine **13g** (269 g, 87%). ^{1}H NMR (600 MHz, CDCl$_3$) δ 8.25 (t, J = 1.8 Hz, 1H), 7.91 (ddd, J = 7.9, 2.0, 1.0 Hz, 1H), 7.86 (br, 1H), 7.79 (dt, J = 8.3, 1.4 Hz, 2H), 7.51 – 7.43 (m, 2H), 7.26 (t, J = 7.9 Hz, 1H), 5.49 – 5.42 (m, 1H), 3.62 (d, J = 13.4 Hz, 1H), 3.47 (d, J = 13.4 Hz, 1H), 2.97 (d, J = 12.5 Hz, 1H), 2.87 (d, J = 13.1 Hz, 1H), 2.44 (dt, J = 8.6, 5.6 Hz, 1H), 2.35 (t, J = 5.3 Hz, 1H), 2.31 (app. d, J = 17.6 Hz, 1H), 2.24 (app. d, J = 17.9 Hz, 1H), 2.17 (s, 3H), 2.14 – 2.08 (m, 1H), 1.32 (s, 3H), 1.17 (d, J = 8.6 Hz, 1H), 0.85 (s, 3H). ^{13}C NMR (151 MHz, CDCl$_3$) δ 153.60, 152.52, 146.44, 141.28, 139.55, 132.16, 130.77, 130.74, 129.08, 123.64, 123.44, 121.85, 120.05, 94.70, 63.84, 61.38, 44.41, 42.74, 41.09, 38.12, 32.02, 31.56, 26.47, 21.29. HPLC-PDA-MS: RT = 4.81 min, 99.9% (254 nm), PDA λ$_{max}$ = 321 nm, MS (m/z) [M+H]$^+$ 486.25

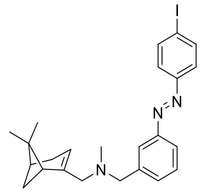

1-((1*R*,5*S*)-6,6-Dimethylbicyclo[3.1.1]hept-2-en-2-yl)-*N*-(3-((*E*)-(4-iodophenyl)diazenyl)benzyl)-*N*-methylmethanamine (13h): The general synthetic procedure A was used with ammonium salt **4** (110 mg, 0.55 mmol), TEA (100 µL, 0.73 mmol) and benzaldehyde **19h** (150 mg, 0.45 mmol) in DCE (10 mL) to give the tertiary amine **13h** (192 g, 89%). ^{1}H NMR (500 MHz, CDCl$_3$) δ 7.91 – 7.83 (m, 3H), 7.82 – 7.76 (m, 1H), 7.66 (d, J = 8.2 Hz, 2H), 7.50 – 7.43 (m, 2H), 5.44 (br, 1H), 3.62 (d, J = 13.4 Hz, 1H), 3.47 (d, J = 13.4 Hz, 1H), 2.97 (d, J = 13.1 Hz, 1H), 2.87 (d, J = 13.0 Hz, 1H), 2.50 – 2.39 (m, 1H), 2.39 – 2.20 (m, 3H), 2.17 (s, 3H), 2.15 – 2.08 (m, 1H), 1.31 (s, 3H), 1.17 (d, J = 8.6 Hz, 1H), 0.85 (s, 3H). ^{13}C NMR (126 MHz, CDCl$_3$) δ 152.59, 152.09, 146.39, 141.22, 138.45, 132.03, 129.08, 124.57, 123.35, 121.79, 120.07, 97.67, 63.83, 61.36, 44.34, 42.75, 41.03, 38.10, 32.00, 31.54, 26.44, 21.28. HPLC-PDA-MS: RT = 5.64 min, 99.7% (254 nm), PDA λ$_{max}$ = 333 nm, MS (m/z) [M+H]$^+$ 486.15.

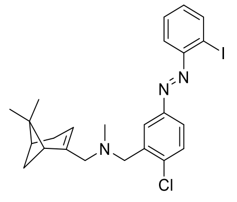

***N*-(2-Chloro-5-((*E*)-(2-iodophenyl)diazenyl)benzyl)-1-((1*R*,5*S*)-6,6-dimethylbicyclo[3.1.1]hept-2-en-2-yl)-*N*-methylmethanamine (14e):** The general synthetic procedure A was used with ammonium salt **7** (116 mg, 0.58 mmol), TEA (90 µL, 0.65 mmol) and benzaldehyde **27** (171 mg, 0.46 mmol) in DCE (9 mL) to give the tertiary amine **14e** (199 g, 83%). ^{1}H NMR (600 MHz, CDCl$_3$) δ 8.19 (d, J = 2.4 Hz, 1H), 8.04 (d, J = 7.6 Hz, 1H), 7.80 (dd, J = 8.5, 2.5 Hz, 1H), 7.64 (dd, J = 8.0, 1.6 Hz, 1H), 7.48 (d, J = 8.4 Hz, 1H), 7.43 (td, J = 7.7, 1.2 Hz, 1H), 7.17 (td, J = 7.6, 1.6 Hz, 1H), 5.47 (br, 1H), 3.70 (d, J = 14.7 Hz, 1H), 3.60 (d, J = 14.7 Hz, 1H), 3.06 (d, J = 13.1 Hz, 1H), 2.96 (d, J = 13.1 Hz, 1H), 2.46 – 2.34 (m, 2H), 2.34 –

2.18 (m, 5H), 2.12 – 2.06 (m, 1H), 1.28 (s, 3H), 1.14 (d, J = 8.4 Hz, 1H), 0.82 (s, 3H). ^{13}C NMR (151 MHz, $CDCl_3$) δ 151.36, 151.09, 146.45, 140.02, 138.75, 137.34, 132.45, 130.22, 129.06, 126.80, 121.62, 120.05, 117.44, 102.87, 64.24, 58.19, 44.37, 42.79, 41.08, 38.13, 32.05, 31.58, 26.52, 21.23. HPLC-PDA-MS: RT = 5.13 min, 99.4% (254 nm), PDA λ_{max} = 329 nm, MS (m/z) $[M+H]^+$ 520.20.

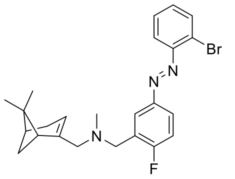

***N*-(5-((*E*)-(2-Bromophenyl)diazenyl)-2-fluorobenzyl)-1-((1*R*,5*S*)-6,6-dimethylbicyclo[3.1.1]hept-2-en-2-yl)-*N*-methylmethanamine (16b):** The synthesis and characterization of this compound have been published by us [1].

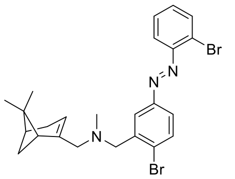

***N*-(2-Bromo-5-((*E*)-(2-bromophenyl)diazenyl)benzyl)-1-((1*R*,5*S*)-6,6-dimethylbicyclo[3.1.1]hept-2-en-2-yl)-*N*-methylmethanamine (16d):** The synthesis and characterization of this compound have been published by us [1].

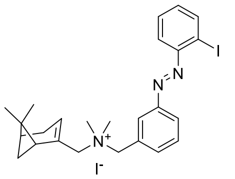

***N*-(3-((*E*)-(2-Iodophenyl)diazenyl)benzyl)-1-((1*R*,5*S*)-6,6-dimethylbicyclo[3.1.1]hept-2-en-2-yl)-*N*,*N*-dimethylmethanaminium iodide (3f)**: The general synthetic procedure D was used with the amine **13f** (176 mg, 0.36 mmol) and MeI (453 µL, 7.3 mmol) in DCM (7.0 mL) to give the ammonium salt **3f** as an orange solid (215 mg, 95%). ^{1}H NMR (600 MHz, $CDCl_3$) δ 8.10 (t, J = 1.9 Hz, 1H), 8.04 (dd, J = 8.3, 1.5 Hz, 1H), 8.02 (dd, J = 7.9, 1.3 Hz, 1H), 7.99 (dt, J = 7.6, 1.4 Hz, 1H), 7.66 – 7.58 (m, 2H), 7.41 (ddd, J = 8.1, 7.2, 1.0 Hz, 1H), 7.18 (td, J = 7.6, 1.6 Hz, 1H), 6.28 (br, 1H), 5.27 (s, 2H), 4.49 (d, J = 12.4 Hz, 1H), 4.32 (d, J = 12.4 Hz, 1H), 3.22 (s, 3H), 3.19 (s, 3H), 2.52 (dt, J = 9.0, 5.6 Hz, 1H), 2.47 – 2.32 (m, 3H), 2.19 – 2.13 (m, 1H), 1.30 (s, 3H), 1.15 (s, 1H), 0.86 (s, 3H). ^{13}C NMR (151 MHz, $CDCl_3$) δ 152.42, 150.71, 139.92, 137.06, 136.31, 135.97, 132.97, 130.42, 129.00, 128.38, 124.76, 117.28, 103.77, 69.20, 66.77, 49.20, 48.94, 47.01, 39.66, 38.12, 32.19, 32.01, 25.93, 21.50. HPLC-PDA-MS: RT = 4.75 min, 99.6% (254 nm), PDA λ_{max} = 322 nm, MS (m/z) $[M]^+$ 500.20. HRMS (m/z): $[M]^+$ calculated for $C_{25}H_{31}IN_3$, 500.1557; found, 500.1547.

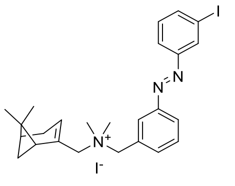

1-((1*R*,5*S*)-6,6-Dimethylbicyclo[3.1.1]hept-2-en-2-yl)-*N*-(3-((*E*)-(3-iodophenyl)diazenyl)benzyl)-*N*,*N*-dimethylmethanaminium iodide (3g): The general synthetic procedure D was used with the amine **13g** (260 mg, 0.54 mmol) and MeI (670 µL, 10.7 mmol) in DCM (11.0 mL) to give the ammonium salt

3g as an orange solid (305 mg, 91%). ^{1}H NMR (600 MHz, CDCl$_3$) δ 8.22 (s, 1H), 8.04 (s, 1H), 7.97 (d, J = 7.6 Hz, 1H), 7.95 (d, J = 8.2 Hz, 1H), 7.91 (d, J = 8.2 Hz, 1H), 7.80 (d, J = 7.7 Hz, 1H), 7.59 (t, J = 7.8 Hz, 1H), 7.26 (t, J = 8.0 Hz, 1H), 6.26 (br, 1H), 5.28 (s, 2H), 4.48 (d, J = 12.4 Hz, 1H), 4.32 (d, J = 12.4 Hz, 1H), 3.21 (s, 3H), 3.19 (s, 3H), 2.51 (dt, J = 9.0, 5.6 Hz, 1H), 2.47 – 2.32 (m, 3H), 2.20 – 2.08 (m, 1H), 1.30 (s, 3H), 1.16 (d, J = 9.0 Hz, 1H), 0.85 (s, 3H). ^{13}C NMR (151 MHz, CDCl$_3$) δ 153.06, 152.50, 140.34, 137.15, 136.41, 136.09, 130.92, 130.88, 130.44, 128.53, 128.04, 124.45, 124.00, 94.73, 69.30, 66.89, 49.31, 49.07, 47.13, 39.78, 38.24, 32.30, 32.13, 26.05, 21.58. HPLC-PDA-MS: RT = 4.58 min, 99.9% (254 nm), PDA λ$_{max}$ = 317, nm, MS (m/z) [M]$^+$ 500.25. HRMS (m/z): [M]$^+$ calculated for $C_{25}H_{31}IN_3$, 500.1557; found, 500.1541.

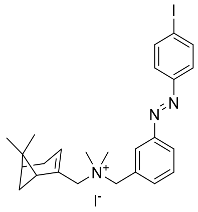

***N*-(3-((E)-(4-Iodophenyl)diazenyl)benzyl)-1-((1*R*,5*S*)-6,6-dimethylbicyclo[3.1.1]hept-2-en-2-yl)-*N*,*N*-dimethylmethanaminium iodide (3h)**: The general synthetic procedure D was used with the amine **13h** (112 mg, 0.23 mmol) and MeI (289 µL, 4.6 mmol) in DCM (4.5 mL) to give the ammonium salt **3h** as an orange solid (141 mg, 97%). ^{1}H NMR (600 MHz, CDCl$_3$) δ 8.05 (t, J = 1.9 Hz, 1H), 7.98 – 7.91 (m, 2H), 7.85 (d, J = 8.5 Hz, 2H), 7.63 (d, J = 8.5 Hz, 2H), 7.58 (t, J = 7.8 Hz, 1H), 6.26 (br, 1H), 5.27 (s, 2H), 4.46 (d, J = 12.5 Hz, 1H), 4.30 (d, J = 12.4 Hz, 1H), 3.20 (s, 3H), 3.18 (s, 3H), 2.52 (dt, J = 8.9, 5.6 Hz, 1H), 2.47 – 2.31 (m, 3H), 2.20 – 2.12 (m, 1H), 1.30 (s, 3H), 1.16 (d, J = 9.0 Hz, 1H), 0.85 (s, 3H). ^{13}C NMR (151 MHz, CDCl$_3$) δ 152.66, 151.64, 138.61, 137.16, 136.20, 136.07, 130.42, 128.48, 128.00, 124.82, 124.41, 98.87, 69.32, 66.98, 49.31, 49.07, 47.14, 39.78, 38.24, 32.31, 32.14, 26.05, 21.58. HPLC-PDA-MS: RT = 4.99 min, 100% (254 nm), PDA λ$_{max}$ = 333, nm, MS (m/z) [M]$^+$ 500.20. HRMS (m/z): [M]$^+$ calculated for $C_{25}H_{31}IN_3$, 500.1557; found, 500.1542.

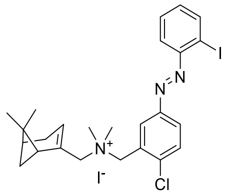

***N*-(5-((*E*)-(2-Iodophenyl)diazenyl)-2-chlorobenzyl)-1-((1*R*,5*S*)-6,6-dimethylbicyclo[3.1.1]hept-2-en-2-yl)-*N*,*N*-dimethylmethanaminium iodide (4e)**: The general synthetic procedure D was used with the amine **14e** (195 mg, 0.38 mmol) and MeI (469 µL, 7.5 mmol) in DCM (7.5 mL) to give the ammonium salt **4e** as an orange solid (203 mg, 82%). ^{1}H NMR (600 MHz, CDCl$_3$) δ 8.53 (d, J = 2.3 Hz, 1H), 8.05 – 7.98 (m, 2H), 7.68 (d, J = 7.6 Hz, 1H), 7.64 (d, J = 8.6 Hz, 1H), 7.43 (t, J = 7.6 Hz, 1H), 7.20 (t, J = 7.5 Hz, 1H), 6.39 (br, 1H), 5.30 (d, J = 13.2 Hz, 1H), 5.24 (d, J = 13.2 Hz, 1H), 4.69 (d, J = 12.4 Hz, 1H), 4.46 (d, J = 12.4 Hz, 1H), 3.27 (s, 6H), 2.65 – 2.52 (m, 1H), 2.52 – 2.35 (m, 3H), 2.25 – 2.14 (m, 1H), 1.33 (s, 3H), 1.24 (d, J = 8.9 Hz, 1H), 0.88 (s, 3H). ^{13}C NMR (151 MHz, CDCl$_3$) δ 151.12, 150.69, 140.08, 139.25, 137.81, 136.07, 133.48, 132.82, 131.84, 129.17, 126.62, 124.60, 117.61, 104.53, 70.46, 63.20, 49.79, 49.54, 47.27, 39.80, 38.32, 32.40, 32.12, 26.06, 21.61. HPLC-PDA-MS: RT = 4.69 min, 99.9% (254 nm), PDA λ$_{max}$ = 212, 329 nm, MS (m/z) [M]$^+$ 534.25. HRMS (m/z): [M]$^+$ calculated for $C_{25}H_{30}ClIN_3$, 534.1167; found, 534.1175.

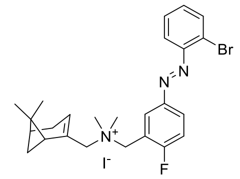

***N*-(5-((*E*)-(2-Bromophenyl)diazenyl)-2-fluorobenzyl)-1-((1*R*,5*S*)-6,6-dimethylbicyclo[3.1.1]hept-2-en-2-yl)-*N*,*N*-dimethylmethanaminium iodide (6b)**: The synthesis and characterization of this compound have been published by us [1].

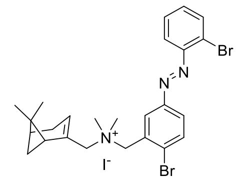

***N*-(5-((*E*)-(2-Bromophenyl)diazenyl)-2-bromobenzyl)-1-((1*R*,5*S*)-6,6-dimethylbicyclo[3.1.1]hept-2-en-2-yl)-*N*,*N*-dimethylmethanaminium iodide (6d)**: The synthesis and characterization of this compound have been published by us [1].

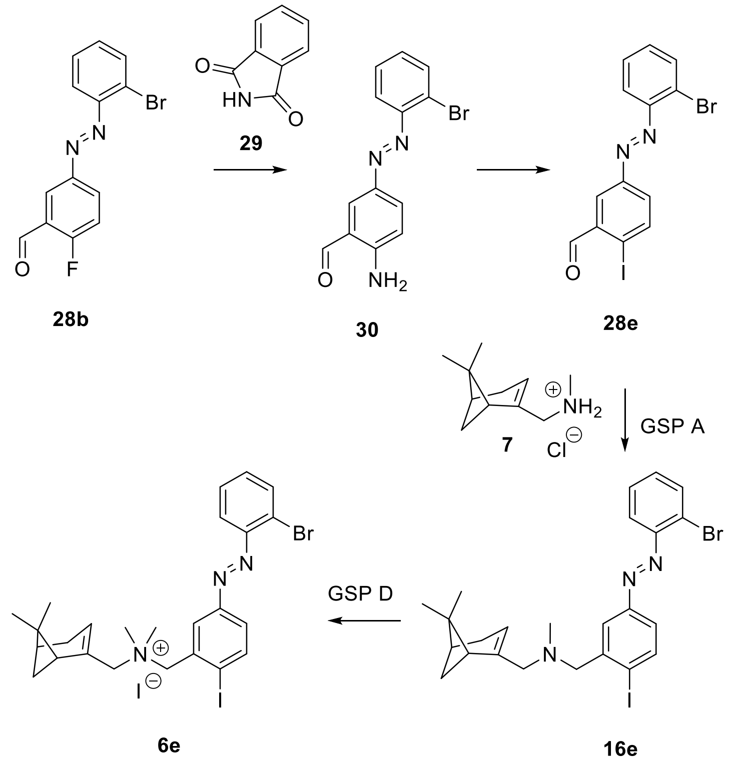

Scheme S3: Synthetic strategy for compound **6e**. GSP=General Synthetic Procedure.

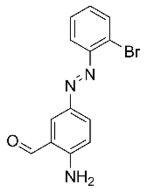

(E)-2-Amino-5-((2-bromophenyl)diazenyl)benzaldehyde (30): A solution of phtalimide (195 mg, 1.32 mmol), benzaldehyde **28b** (271 mg, 0.88 mmol) and K_2CO_3 (244 mg, 1.77 mmol) in DMF (6.5 mL) was heated in the microwave at 65 °C for 3 h. Water (30 mL) was added and the mixture was allowed to cool to room temperature. Slightly acidic water (50 mL) and DCM (50 mL) were added. The layers were separated. The organic layer was washed with slightly acidic brine (3 × 30 mL), dried over Na_2SO_4 and concentrated to give a red solid. The solid was dissolved in AcOH (50 mL). The mixture was heated to reflux for 1 h. Around 30 mL of the solvent were removed in vacuo and water was added slowly to induce precipitation. The suspension was heated to reflux to re-dissolve the solids and slowly cooled to 0 °C to crystallize the product. The crystals were filtered and washed with cold water (3 × 5 mL). The crystalline red solid was purified with automated flash chromatography (cHex/EtOAc) to give compound **30** (179 mg, 67%) as a red solid. ^{1}H NMR (600 MHz, $CDCl_3$) δ 10.01 (s, 1H), 8.21 (d, J = 2.3 Hz, 1H), 8.06 (dd, J = 8.9, 2.3 Hz, 1H), 7.73 (dd, J = 8.0, 1.3 Hz, 1H), 7.66 (dd, J = 8.0, 1.6 Hz, 1H), 7.38 (ddd, J = 8.2, 7.3, 1.3 Hz, 1H), 7.28 (td, J = 8.5, 7.8, 1.6 Hz, 1H), 6.75 (d, J = 8.9 Hz, 1H), 6.62 (br, J = 8.9 Hz, 2H). ^{13}C NMR (151 MHz, $CDCl_3$) δ 93.91, 152.54, 149.76, 144.15, 134.95, 133.80, 131.24, 128.40, 128.12, 125.18, 118.14, 117.88, 116.98. HPLC-PDA-MS: RT = 5.05 min, 98.2% (254 nm), PDA λ_{max} = 375 nm, MS (m/z) $[M+H]^+$ 304.05, 306.00.

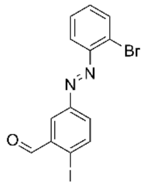

(*E*)-5-((2-Bromophenyl)diazenyl)-2-iodobenzaldehyde (28e): To a solution of pTsOH·H_2O (0.139 g, 0.730 mmol) in MeCN (6 mL) was added amine **30** (74 mg, 0.243 mmol). The suspension of ammonium salt was cooled to 10-15 °C and to this was added gradually a solution of $NaNO_2$ (34 mg, 0.487 mmol) and KI (101 mg, 0.61 mmol) in water (0.9 mL). The reaction mixture was stirred for 10 min while allowed to warm to rt, after which it was stirred for 2 h. Water (10 mL) and satd. aq. $NaHCO_3$ were added (until pH = 9-10), followed by aq. $Na_2S_2O_3$ (1 M, 5 mL). The precipitate was filtered and purified with automated flash chromatography (cHex/DCM) to give compound **28e** (13 mg, 13%) as an orange solid. ^{1}H NMR (600 MHz, $CDCl_3$) δ 10.16 (s, 1H), 8.44 (d, J = 2.5 Hz, 1H), 8.12 (d, J = 8.4 Hz, 1H), 7.86 (dd, J = 8.4, 2.5 Hz, 1H), 7.77 (dd, J = 7.8, 1.4 Hz, 1H), 7.70 (dd, J = 7.9, 1.7 Hz, 1H), 7.40 (td, J = 7.6, 1.4 Hz, 1H), 7.36 (td, J = 7.6, 1.7 Hz, 1H). ^{13}C NMR (151 MHz, $CDCl_3$) δ 195.03, 152.71, 149.37, 141.76, 136.26, 134.13, 132.95, 128.20, 127.59, 126.73, 126.63, 117.85, 103.48. HPLC-PDA-MS: RT = 5.15 min, 80.3 % (254 nm), PDA λ_{max} = 377 nm, MS (m/z) $[M+H]^+$ no clear MS signal detectable

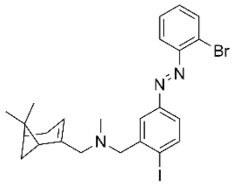

***N*-(5-((*E*)-(2-Bromophenyl)diazenyl)-2-iodobenzyl)-1-((1*R*,5*S*)-6,6-dimethylbicyclo[3.1.1]hept-2-en-2-yl)-*N*-methylmethanamine (16e):** The general synthetic procedure A was used with ammonium salt **7** (20 mg, 0.10 mmol), TEA (14 µL, 0.10 mmol), benzaldehyde **28e** (33 mg, 0.08 mmol) and $NaBH(OAc)_3$ (27 mg, 0.13 mmol) in DCE (1.6 mL) to give the tertiary amine **16e** (24 mg, 54%) as an orange oil. ^1H NMR (600 MHz, $CDCl_3$) δ 8.11 (br, 1H), 8.00 (d, J = 8.4 Hz, 1H), 7.78 (dd, J = 8.0, 1.3 Hz, 1H), 7.72 (d, J = 7.5 Hz, 1H), 7.57 (d, J = 6.4 Hz, 1H), 7.42 (td, J = 7.6, 1.3 Hz, 1H), 7.35 (td, J = 7.6, 1.7 Hz, 1H), 5.51 (br, 1H), 3.62 (s, 2H), 3.16 – 2.87 (m, 2H), 2.49 – 2.37 (m, 2H), 2.37 – 2.22 (m, 5H), 2.17 – 2.08 (m, 1H), 1.31 (s, 3H), 1.16 (d, J = 8.3 Hz, 1H), 0.85 (s, 3H). ^{13}C NMR (151 MHz, $CDCl_3$) δ 52.75, 149.68, 140.93, 140.26, 133.94, 132.24, 128.13, 126.13, 124.42, 121.83, 117.91, 109.53, 104.28, 61.28, 59.03, 44.47, 42.64, 41.01, 38.13, 32.01, 31.60, 26.44, 21.27, 21.27. HPLC-PDA-MS: RT = 5.50 min, 97.7 % (254 nm), PDA λ_{max} = 338 nm, MS (m/z) $[M+H]^+$ 564.05, 566.15.

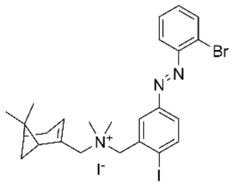

***N*-(5-((*E*)-(2-Bromophenyl)diazenyl)-2-iodobenzyl)-1-((1*R*,5*S*)-6,6-dimethylbicyclo[3.1.1]hept-2-en-2-yl)-*N*,*N*-dimethylmethanaminium iodide (6e, VUF16620):** The general synthetic procedure D was used with the amine **16e** (24 mg, 0.04 mmol) and MeI (53 µL, 0.85 mmol) in DCM (0.9 mL) to give the ammonium salt **6e** as an orange solid (14.3 mg, 57%). ^1H NMR δ 8.50 (d, J = 2.4 Hz, 1H), 8.11 (d, J = 8.5 Hz, 1H), 7.75 (t, J = 7.2 Hz, 2H), 7.69 (dd, J = 8.6, 2.3 Hz, 1H), 7.40 (t, J = 7.5 Hz, 1H), 7.36 (t, J = 7.5 Hz, 1H), 6.40 (br, 1H), 5.31 (d, J = 13.5 Hz, 1H), 5.28 (d, J = 13.7 Hz, 1H), 4.71 (d, J = 12.5 Hz, 1H), 4.41 (d, J = 12.4 Hz, 1H), 3.31 (s, 3H), 3.28 (s, 3H), 2.63 – 2.53 (m, 1H), 2.53 – 2.35 (m, 3H), 2.24 – 2.16 (m, 1H), 1.37 – 1.28 (m, 4H), 0.88 (s, 3H).^{13}C NMR (151 MHz, $CDCl_3$) δ 152.74, 149.09, 142.23, 137.88, 135.96, 134.04, 133.27, 132.43, 131.94, 128.31, 127.10, 123.51, 118.11, 107.86, 70.72, 69.42, 50.06, 49.68, 47.32, 39.83, 38.41, 32.44, 32.14, 26.05, 21.58.HPLC-PDA-MS: *trans*-**6e** RT = 4.89 min, 96.8% (254 nm), PDA λ_{max} = 337 nm, MS (m/z) $[M]^+$ 578.10, 570.10; *cis*-**6e** RT = 4.62 min, 2.1% (254 nm), PDA λ_{max} = 293, 420 nm, MS (m/z) $[M]^+$ 578.10, 570.10. HRMS (m/z): $[M]^+$ calculated for $C_{25}H_{30}BrIN_3$, 578.0662; found, 578.0661.

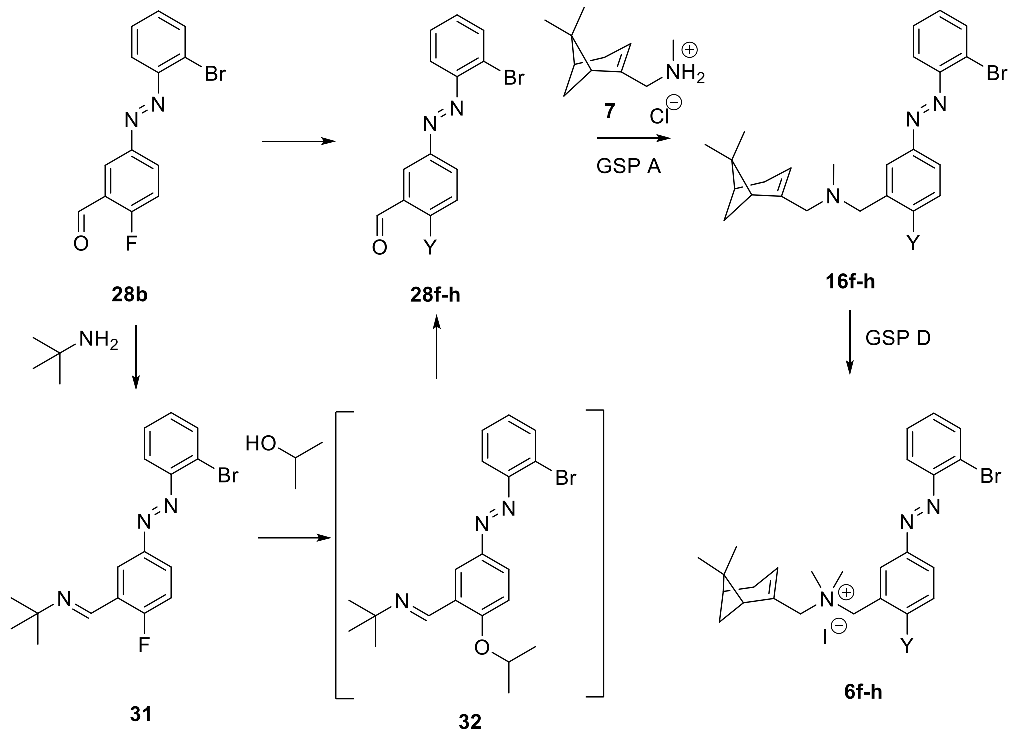

Scheme S4: Synthetic strategies for compounds **6f-h**. GSP = General Synthetic Procedure.

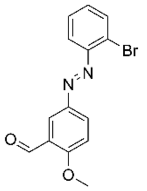

(*E*)-5-((2-Bromophenyl)diazenyl)-2-methoxybenzaldehyde (28f): The synthesis and characterization of this compound have been published by us [1], but here we report an alternative procedure. NaOMe (30% in MeOH, 50 µL, 0.27 mmol) was added to a solution of fluoride **28b** (66 mg, 0.22 mmol) in anhydrous MeOH (4.5 mL). The mixture was heated in the MW at 65 °C for 60 min. The product partially crystallised. Water (4.5 mL) was added. The resulting suspension was filtered and washed with water (3 × 4.5 mL) to give an orange solid which was dried in the oven overnight. This afforded compound **28f** as an orange solid (62 mg, 90%), which was used without further purification. ^{1}H NMR (600 MHz, $CDCl_3$) δ 10.53 (s, 1H), 8.48 (d, J = 2.6 Hz, 1H), 8.22 (dd, J = 8.9, 2.6 Hz, 1H), 7.75 (dd, J = 8.0, 1.3 Hz, 1H), 7.67 (dd, J = 8.0, 1.7 Hz, 1H), 7.39 (td, J = 7.6, 1.3 Hz, 1H), 7.31 (td, J = 7.6, 1.7 Hz, 1H), 7.15 (d, J = 8.9 Hz, 1H), 4.05 (s, 3H). ^{13}C NMR (151 MHz, $CDCl_3$) δ 189.27, 164.00, 149.59, 146.71, 133.92, 131.99, 129.33, 128.14, 125.85, 125.84, 125.24, 117.90, 112.41, 56.40. HPLC-PDA-MS: RT = 5.32 min, 99.0% (254 nm), PDA λ_{max} = 338 nm, MS (m/z) $[M+H]^+$ 319.00, 321.05.

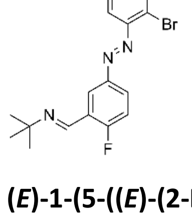

(*E*)-1-(5-((*E*)-(2-Bromophenyl)diazenyl)-2-isopropoxyphenyl)-*N*-(*tert*-butyl)methanimine (31): A solution of benzaldehyde **28b** (650 mg, 2.1 mmol) and *tert*-butylamine (227 µL, 2.2 mmol) in PhMe was heated to reflux with a Dean-Stark system for 20 h. The solvent was removed in vacuo to give an

orange solid that corresponds to **31** (755 mg, 99%) which was used as such in further reactions. ^{1}H NMR (600 MHz, $CDCl_3$) δ 8.68 (dd, J = 6.8, 2.6 Hz, 1H), 8.61 (s, 1H), 7.98 (ddd, J = 8.7, 4.9, 2.6 Hz, 1H), 7.75 (dd, J = 7.9, 1.3 Hz, 1H), 7.67 (dd, J = 8.0, 1.6 Hz, 1H), 7.42 – 7.36 (m, 1H), 7.32 (td, J = 7.6, 1.7 Hz, 1H), 7.21 (t, J = 9.3 Hz, 1H), 1.34 (s, 9H). ^{13}C NMR (151 MHz, $CDCl_3$) δ 164.77, 163.06, 149.61, 149.33, 149.31, 147.92, 147.90, 133.92, 132.13, 128.14, 126.22, 126.19, 125.92, 125.73, 125.66, 123.68, 123.61, 117.94, 116.91, 116.75, 58.35, 29.78. HPLC-PDA-MS: RT = 5.43 min, >99 % (254 nm), PDA λ_{max} = 320 nm, MS (m/z) $[M+H]^+$ 306.95, 308.95, hydrolysis product (aldehyde) observed in MS.

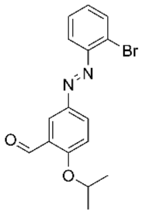

(*E*)-5-((2-Bromophenyl)diazenyl)-2-isopropoxybenzaldehyde (28g): Anhydrous 2-PrOH (40 µL, 0.53 mmol) was added to a suspension of NaH (13 mg, 0.53 mmol) in anhydrous DMSO (1.5 mL). It was stirred for 30 min. Fluoride **31** (181 mg, 0.50 mmol) was added. The dark blue mixture was heated to 100 °C for 1 h, during which the colour turned to dark red. The reaction mixture was poured into water (40 mL) and extraction with EtOAc (3 × 20 mL) was performed. The combined organic layers were washed with brine, dried over $MgSO_4$, filtered and concentrated in vacuo to give a pink oil. This residue containing intermediate **32** was dissolved in THF (5 mL). Water (1.5 mL) and AcOH (100 µL) were added. The mixture was stirred for 16 h at rt. The solvent was removed *in vacuo* and the obtained residue was redissolved in EtOAc/water 3:2 (50 mL). The layers were separated. The organic phase was washed with brine, dried over $MgSO_4$, filtered and concentrated *in vacuo* to give a residue that was purified with automated flash chromatography (cHex/DCM) to give **28g** as an orange solid (138 mg, 79%). ^{1}H NMR (600 MHz, $CDCl_3$) δ 10.54 (s, 1H), 8.47 (d, J = 2.6 Hz, 1H), 8.17 (dd, J = 9.0, 2.6 Hz, 1H), 7.74 (dd, J = 7.9, 1.3 Hz, 1H), 7.66 (dd, J = 8.1, 1.6 Hz, 1H), 7.38 (ddd, J = 8.0, 7.2, 1.4 Hz, 1H), 7.30 (ddd, J = 8.0, 7.2, 1.7 Hz, 1H), 7.12 (d, J = 9.0 Hz, 1H), 4.81 (hept, J = 6.1 Hz, 1H), 1.47 (d, J = 6.1 Hz, 6H). ^{13}C NMR (151 MHz, $CDCl_3$) δ 189.55, 162.89, 149.60, 146.32, 133.87, 131.89, 128.73, 128.12, 126.05, 125.84, 125.78, 117.90, 114.29, 71.94, 22.12. HPLC-PDA-MS: RT = 5.84 min, 94.4% (254 nm), PDA λ_{max} = 341 nm, MS (m/z) $[M+H]^+$ 347.00, 349.00.

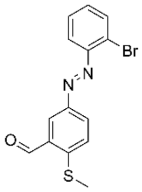

(*E*)-5-((2-Bromophenyl)diazenyl)-2-(methylthio)benzaldehyde (28h): A solution of fluoride **28b** (76 mg, 0.25 mmol) and NaSMe (18 mg, 0.25 mmol) in DMF (3 mL) was heated in the MW at 65 °C for 30 min. Water (6 mL) was added. The suspension was filtered. The solid was washed with water (3 × 3 mL) and dried in the oven overnight to give **28h** as an orange solid (73 mg, 88%), which was used as such for further reactions. ^{1}H NMR (600 MHz, $CDCl_3$) δ 10.30 (s, 1H), 8.41 (s, 1H), 8.14 (d, J = 7.7 Hz, 1H), 7.76 (d, J = 7.9 Hz, 1H), 7.70 (d, J = 8.0 Hz, 1H), 7.46 (d, J = 8.6 Hz, 1H), 7.40 (t, J = 7.7 Hz, 1H), 7.33 (t, J = 7.7 Hz, 1H), 2.58 (s, 3H). ^{13}C NMR (151 MHz, $CDCl_3$) δ 191.13, 149.53, 149.29, 147.83, 134.01, 132.91, 132.35, 129.93, 128.17, 127.25, 126.19, 125.33, 117.85, 15.45. HPLC-PDA-MS: RT = 5.51 min, 92.1 % (254 nm), PDA λ_{max} = 353 nm, MS (m/z) $[M+H]^+$ 334.90, 337.05.

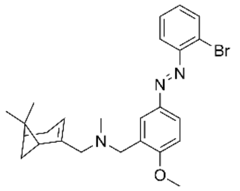

***N*-(5-((*E*)-(2-Bromophenyl)diazenyl)-2-methoxybenzyl)-1-((1*R*,5*S*)-6,6-dimethylbicyclo[3.1.1]hept-2-en-2-yl)-*N*-methylmethanamine (16f):** The synthesis and characterization of this compound have been published by us [1].

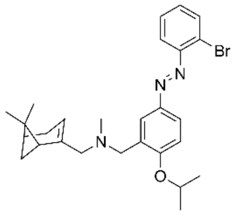

***N*-(5-((*E*)-(2-Bromophenyl)diazenyl)-2-isopropoxybenzyl)-1-((1*R*,5*S*)-6,6-dimethylbicyclo[3.1.1] hept-2-en-2-yl)-*N*-methylmethanamine (16g):** The general synthetic procedure A was used with ammonium salt **7** (94 mg, 0.47 mmol), TEA (68 µL, 0.49 mmol), benzaldehyde **28g** (130 mg, 0.37 mmol) and $NaBH(OAc)_3$ (127 mg, 0.60 mmol) in DCE (7.5 mL) to give the tertiary amine **16g** (172 mg, 93%) as an orange oil. ^1H NMR (600 MHz, $CDCl_3$) δ 8.10 (d, J = 2.5 Hz, 1H), 7.86 (dd, J = 8.8, 2.5 Hz, 1H), 7.73 (dd, J = 8.0, 1.3 Hz, 1H), 7.65 (dd, J = 8.0, 1.6 Hz, 1H), 7.37 (td, J = 7.6, 1.3 Hz, 1H), 7.28 – 7.24 (m, 1H), 6.96 (d, J = 8.8 Hz, 1H), 5.46 (br, 1H), 4.67 (hept, J = 6.0 Hz, 1H), 3.60 (d, J = 14.2 Hz, 1H), 3.55 (d, J = 14.4 Hz, 1H), 3.02 (d, J = 14.1 Hz, 1H), 2.92 (d, J = 13.2 Hz, 1H), 2.42 (dt, J = 8.6, 5.6 Hz, 1H), 2.37 (t, J = 5.7 Hz, 1H), 2.31 (app. d, J = 17.6 Hz, 1H), 2.23 (app. d, J = 17.0 Hz, 2H), 2.20 (s, 3H), 2.15 – 2.05 (m, 1H), 1.39 (d, J = 5.7 Hz, 6H), 1.29 (s, 3H), 1.16 (d, J = 8.5 Hz, 1H), 0.84 (s, 3H). ^{13}C NMR (151 MHz, $CDCl_3$) δ 159.38, 150.09, 146.74, 133.70, 131.10, 129.52, 128.06, 126.51, 125.18, 123.36, 119.59, 117.95, 112.57, 100.13, 70.64, 64.00, 54.81, 44.35, 42.61, 41.13, 38.11, 32.04, 31.58, 26.47, 22.27, 21.25. HPLC-PDA-MS: RT = 5.46 min, 89.9% (254 nm), PDA λ_{max} = 352 nm, MS (m/z) $[M+H]^+$ 496.30, 498.35.

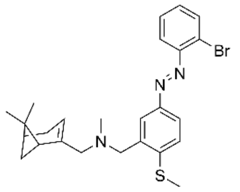

***N*-(5-((*E*)-(2-Bromophenyl)diazenyl)-2-(methylthio)benzyl)-1-((1*R*,5*S*)-6,6-dimethylbicyclo[3.1.1] hept-2-en-2-yl)-*N*-methylmethanamine (16h):** The general synthetic procedure A was used with ammonium salt **7** (72 mg, 0.36 mmol), TEA (56 µL, 0.40 mmol), benzaldehyde **28h** (96 mg, 0.29 mmol) and $NaBH(OAc)_3$ (97 mg, 0.46 mmol) in DCE (5.7 mL) to give the tertiary amine **16h** (117 mg, 84%) as an orange oil. ^1H NMR (600 MHz, $CDCl_3$) δ 8.11 (br, 1H), 8.00 (d, J = 8.4 Hz, 1H), 7.78 (dd, J = 8.0, 1.3 Hz, 1H), 7.72 (d, J = 7.5 Hz, 1H), 7.57 (d, J = 6.4 Hz, 1H), 7.42 (td, J = 7.6, 1.3 Hz, 1H), 7.35 (td, J = 7.6, 1.7 Hz, 1H), 5.51 (br, 1H), 3.62 (s, 2H), 3.16 – 2.87 (m, 2H), 2.49 – 2.37 (m, 2H), 2.37 – 2.22 (m, 5H), 2.17 – 2.08 (m, 1H), 1.31 (s, 3H), 1.16 (d, J = 8.3 Hz, 1H), 0.85 (s, 3H). ^{13}C NMR (151 MHz, $CDCl_3$) δ 52.75, 149.68, 140.93, 140.26, 133.94, 132.24, 128.13, 126.13, 124.42, 121.83, 117.91, 109.53, 104.28, 61.28, 59.03, 44.47, 42.64, 41.01, 38.13, 32.01, 31.60, 26.44, 21.27, 21.27. HPLC-PDA-MS: RT = 5.14 min, 98.8% (254 nm), PDA λ_{max} = 367 nm, MS (m/z) $[M+H]^+$ 484.20, 486.20.

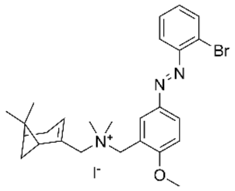

***N*-(5-((*E*)-(2-Bromophenyl)diazenyl)-2-methoxybenzyl)-1-((1*R*,5*S*)-6,6-dimethylbicyclo[3.1.1]hept-2-en-2-yl)-*N*,*N*-dimethylmethanaminium iodide (6f, VUF16216):** The synthesis and characterization of this compound have been published by us [1].

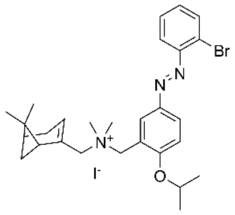

***N*-(5-((*E*)-(2-Bromophenyl)diazenyl)-2-isopropoxybenzyl)-1-((1*R*,5*S*)-6,6-dimethylbicyclo[3.1.1] hept-2-en-2-yl)-*N*,*N*-dimethylmethanaminium iodide (6g):** The general synthetic procedure D was used with the amine **16g** (153 mg, 0.31 mmol) and MeI (385 µL, 6.2 mmol) in DCM (6.0 mL) to give the ammonium salt **6g** as an orange solid (171 mg, 87%). ^{1}H NMR δ 8.29 (d, J =2.4 Hz, 1H), 8.07 (dd, J = 9.0, 2.4 Hz, 1H), 7.73 (dd, J = 8.0, 1.3 Hz, 1H), 7.70 (dd, J = 8.1, 1.6 Hz, 1H), 7.39 (td, J = 7.6, 1.3 Hz, 1H), 7.31 (td, J = 7.6, 1.7 Hz, 1H), 7.10 (d, J = 9.0 Hz, 1H), 6.38 (br, 1H), 4.88 (s, 2H), 4.79 (p, J = 6.1 Hz, 1H), 4.60 (d, J = 12.5 Hz, 1H), 4.35 (d, J = 12.5 Hz, 1H), 3.21 (s, 3H), 3.20 (s, 3H), 2.55 (dt, J = 8.9, 5.6 Hz, 1H), 2.51 – 2.36 (m, 3H), 2.23 – 2.16 (m, 1H), 1.43 (d, J = 6.0 Hz, 6H), 1.33 (s, 3H), 1.25 (d, J = 8.8 Hz, 1H), 0.89 (s, 3H).^{13}C NMR (151 MHz, $CDCl_3$) δ 158.62, 148.34, 145.44, 136.16, 135.37, 132.84, 132.53, 131.19, 127.22, 125.06, 124.15, 117.07, 115.79, 112.67, 70.93, 69.29, 61.00, 48.76, 48.69, 46.29, 38.84, 37.30, 31.36, 31.12, 25.07, 21.27, 20.60. HPLC-PDA-MS: *trans*-**6g** RT = 5.32 min, 98.6% (254 nm), PDA λ_{max} = 352 nm, MS (m/z) [M]$^+$ 510.30, 512.30; *cis*-**6g** RT = 4.84 min, 1.0% (254 nm), PDA λ_{max} = 309, 429 nm, MS (m/z) [M]$^+$ 510.35, 512.35. HRMS (m/z): [M]$^+$ calculated for $C_{28}H_{37}BrN_3O$, 510.2115; found, 510.2121.

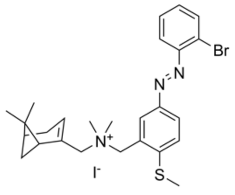

***N*-(5-((*E*)-(2-Bromophenyl)diazenyl)-2-(methylthio)benzyl)-1-((1*R*,5*S*)-6,6-dimethylbicyclo[3.1.1] hept-2-en-2-yl)-*N*,*N*-dimethylmethanaminium iodide (6h):** The general synthetic procedure D was used with the amine **16h** (104 mg, 0.22 mmol) and MeI (268 µL, 4.3 mmol) in DCM (4.5 mL) to give the ammonium salt **6h** as an orange solid (115 mg, 86%). ^{1}H NMR δ 8.33 (d, J = 2.2 Hz, 1H), 8.01 (dd, J = 8.6, 2.1 Hz, 1H), 7.74 (d , J = 8.2 Hz, 1H), 7.72 (d, J = 8.0 Hz, 2H), 7.45 (d, J = 8.7 Hz, 1H), 7.39 (t, J = 7.6 Hz, 1H), 7.33 (t, J = 7.6 Hz, 1H), 6.37 (br, 1H), 5.15 (d, J = 13.4 Hz, 1H), 5.08 (d, J = 13.5 Hz, 1H), 4.63 (d, J = 12.4 Hz, 1H), 4.38 (d, J = 12.4 Hz, 1H), 3.24 (d, J = 6.3 Hz, 6H), 2.58 (s, 3H), 2.57 – 2.52 (m, 1H), 2.50 – 2.36 (m, 3H), 2.20 –2.14 (m, 1H), 1.32 (s, 3H), 1.29 (d, J = 8.9 Hz, 1H), 0.88 (s, 3H).^{13}C NMR (151 MHz,

$CDCl_3$) δ 150.06, 149.24, 145.90, 137.53, 136.15, 133.91, 132.67, 132.56, 128.23, 127.58, 126.61, 125.97, 123.00, 118.05, 70.54, 64.02, 49.89, 49.64, 47.29, 39.81, 38.32, 32.37, 32.05, 26.05, 21.56, 16.81. HPLC-PDA-MS: RT = 4.95 min, 99.2% (254 nm), PDA λ_{max} = 367 nm, MS (m/z) $[M]^+$ 498.30, 500.25. HRMS (m/z): $[M]^+$ calculated for $C_{26}H_{33}BrN_3S$, 498.1565; found, 498.1573.

NMR Spectra

Compound **4d** (VUF15888). Analytical data for this compound has been published by us before [1].

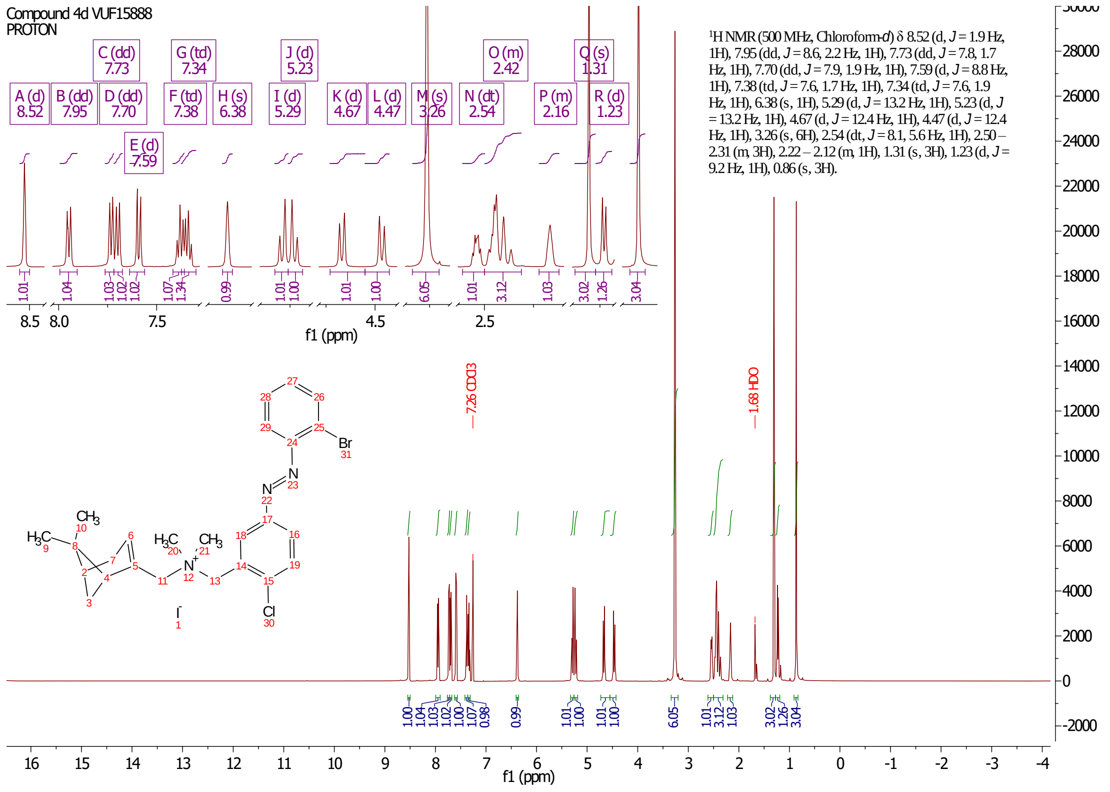

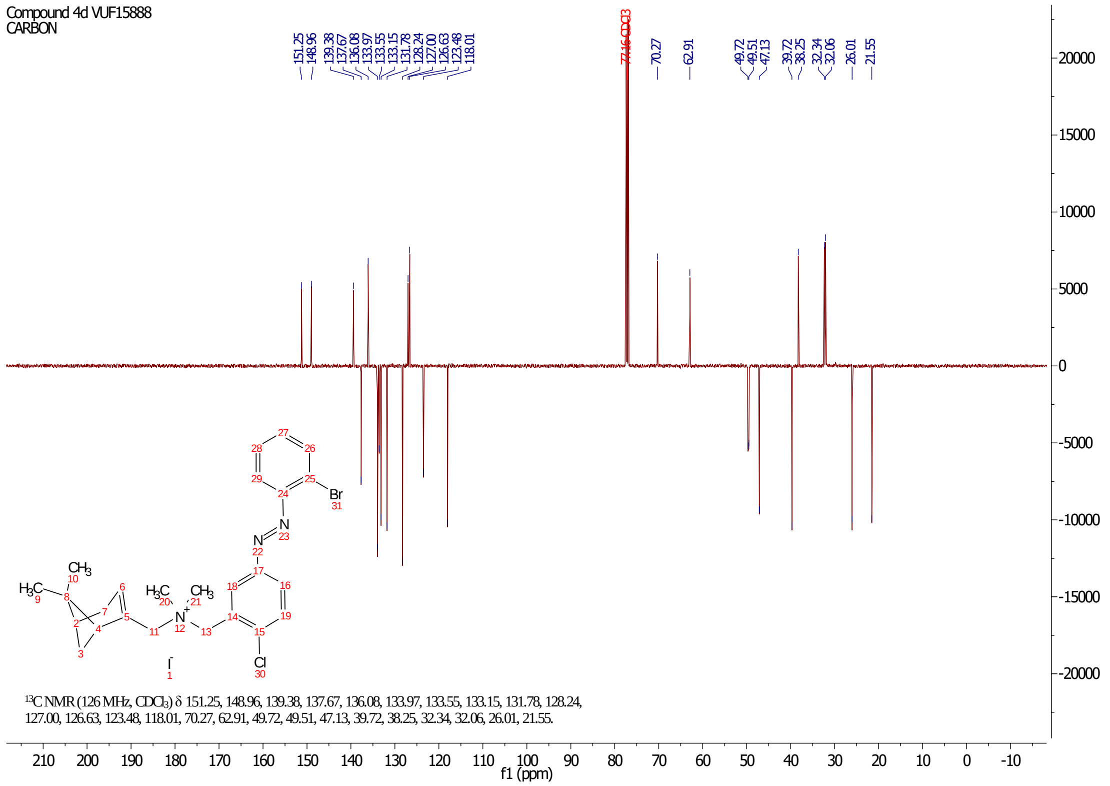

Compound **6e** (VUF16620).

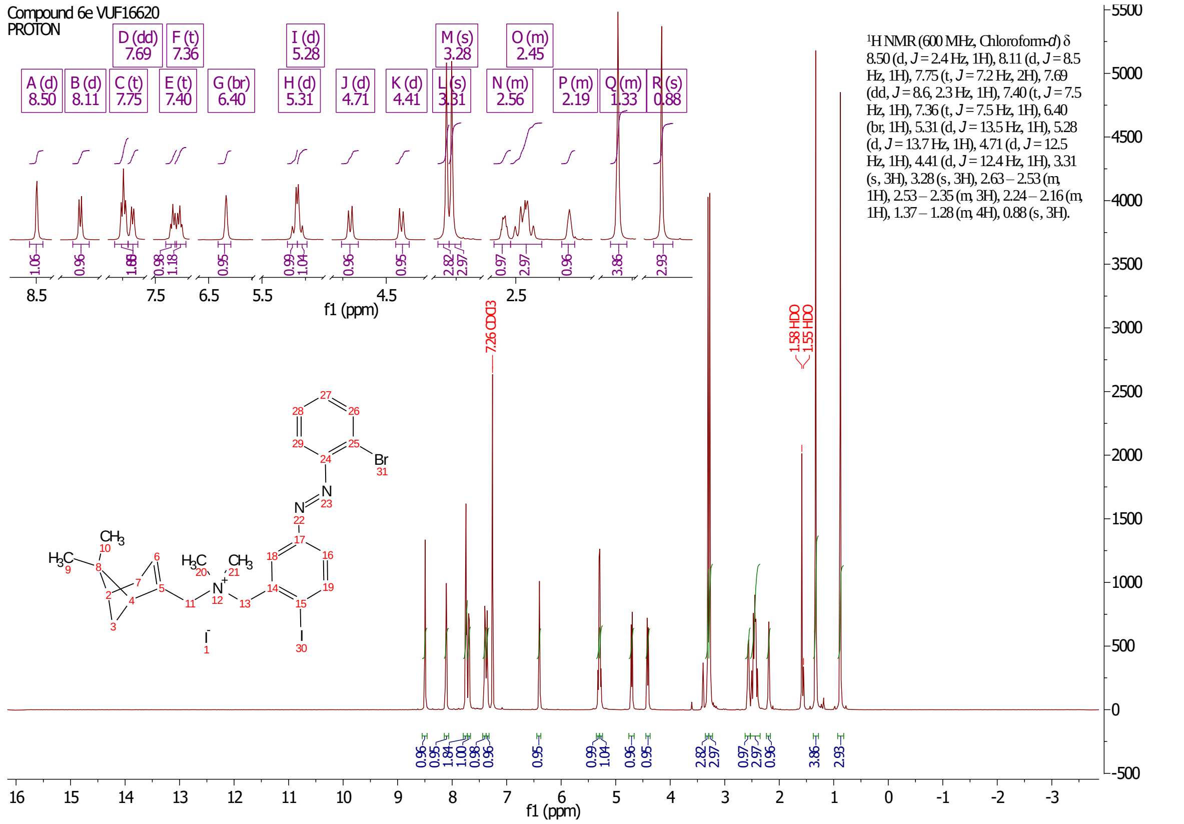

^{1}H NMR (600 MHz, Chloroform-*d*) δ 8.50 (d, *J* = 2.4 Hz, 1H), 8.11 (d, *J* = 8.5 Hz, 1H), 7.75 (t, *J* = 7.2 Hz, 2H), 7.69 (dd, *J* = 8.6, 2.3 Hz, 1H), 7.40 (t, *J* = 7.5 Hz, 1H), 7.36 (t, *J* = 7.5 Hz, 1H), 6.40 (br, 1H), 5.31 (d, *J* = 13.5 Hz, 1H), 5.28 (d, *J* = 13.7 Hz, 1H), 4.71 (d, *J* = 12.5 Hz, 1H), 4.41 (d, *J* = 12.4 Hz, 1H), 3.31 (s, 3H), 3.28 (s, 3H), 2.63 – 2.53 (m, 1H), 2.53 – 2.35 (m, 3H), 2.24 – 2.16 (m, 1H), 1.37 – 1.28 (m, 4H), 0.88 (s, 3H).

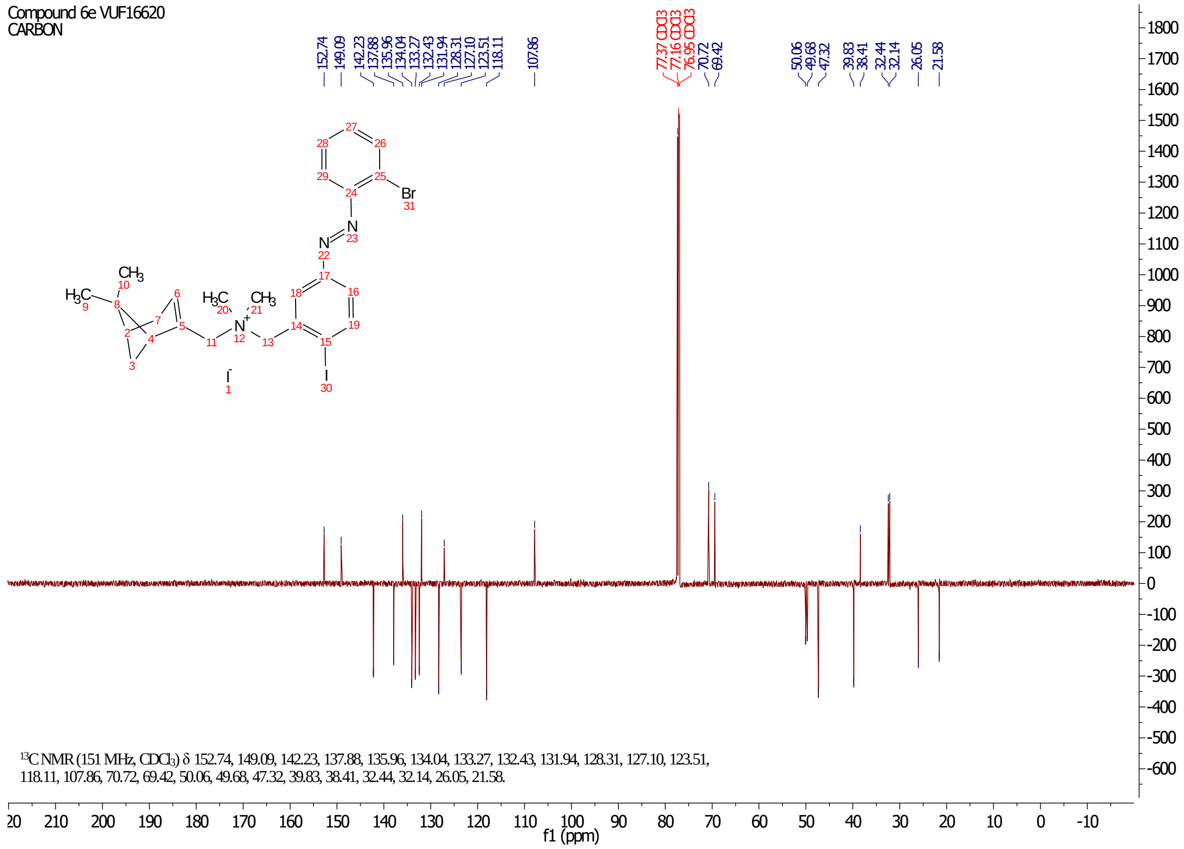

^{13}C NMR (151 MHz, $CDCl_3$) δ 152.74, 149.09, 142.23, 137.88, 135.96, 134.04, 133.27, 132.43, 131.94, 128.31, 127.10, 123.51, 118.11, 107.86, 70.72, 69.42, 50.06, 49.68, 47.32, 39.83, 38.41, 32.44, 32.14, 26.05, 21.58.

Compound **6f** (VUF16216). Analytical data for this compound has been published by us before [1].

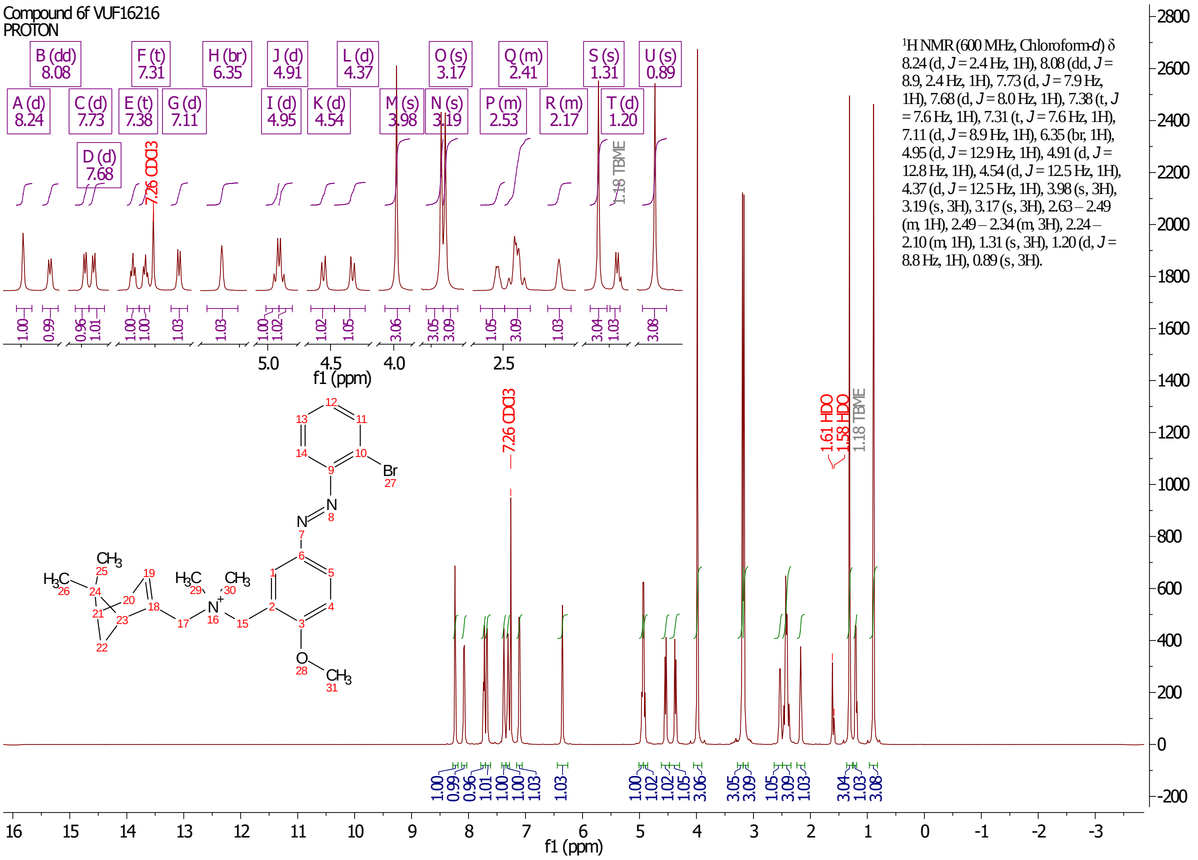

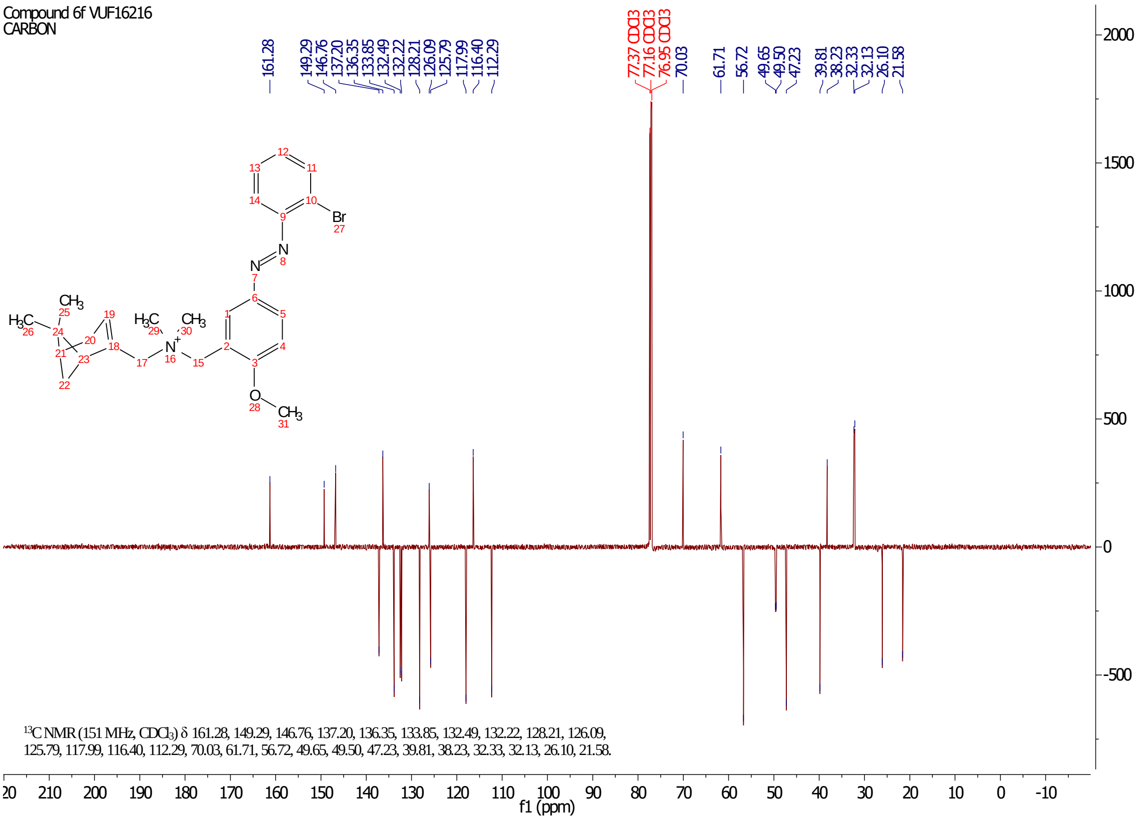

LC-PDA-MS chromatograms

Compound **4d** (VUF15888). The iodide counter ion is visible at 0.8 min. Analytical data for this compound has been published by us before [1].

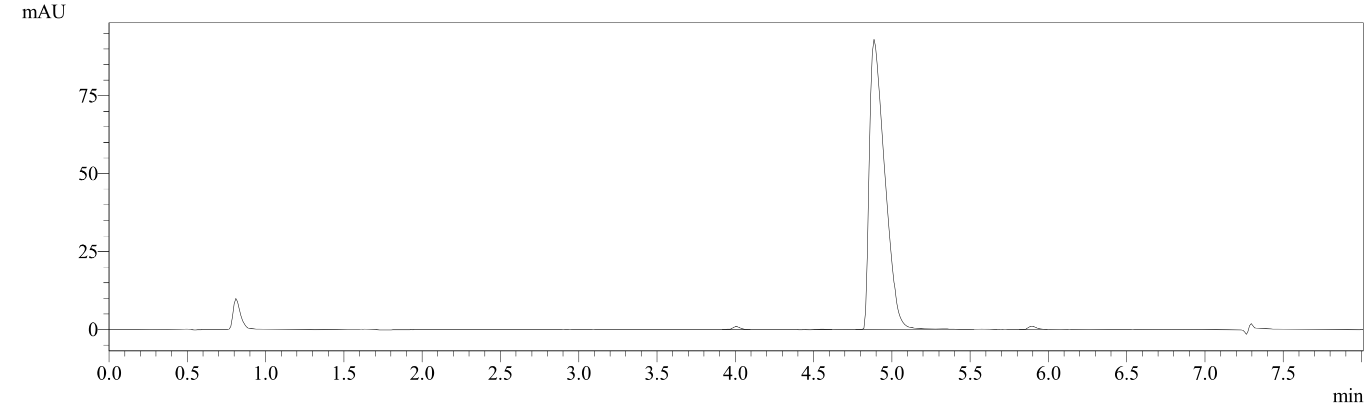

PDA Ch1 254nm 4nm

Peak#	Name	Ret. Time	Area	Area %
1		3.999	3250	0.501
2		4.542	545	0.084
3		4.882	639476	98.612
4		5.339	1061	0.164
5		5.603	276	0.043
6		5.888	3872	0.597

#:1 Ret.Time:Averaged 4.960-4.980(Scan#:497-499)
Mass Peaks:10 Base Peak:488.20(870908) Polarity:Pos Segment1 - Event1

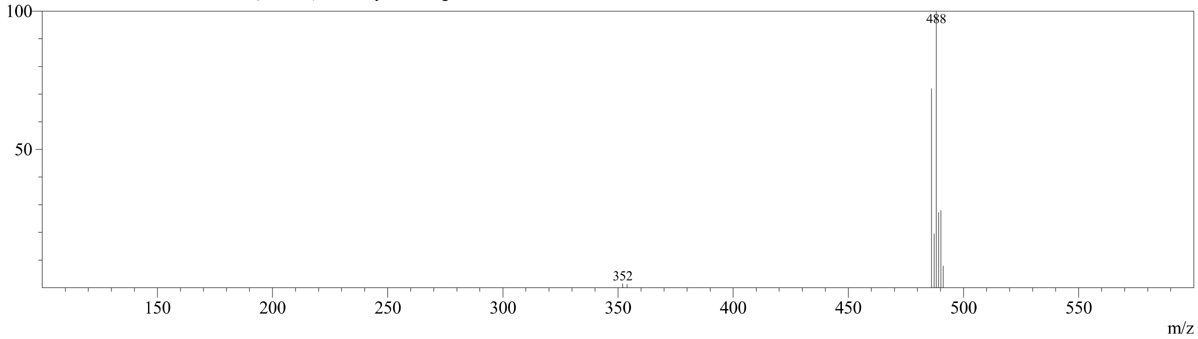

Retention Time : 4.882
Compound Name :
Spectrum Operation : BG Adjust

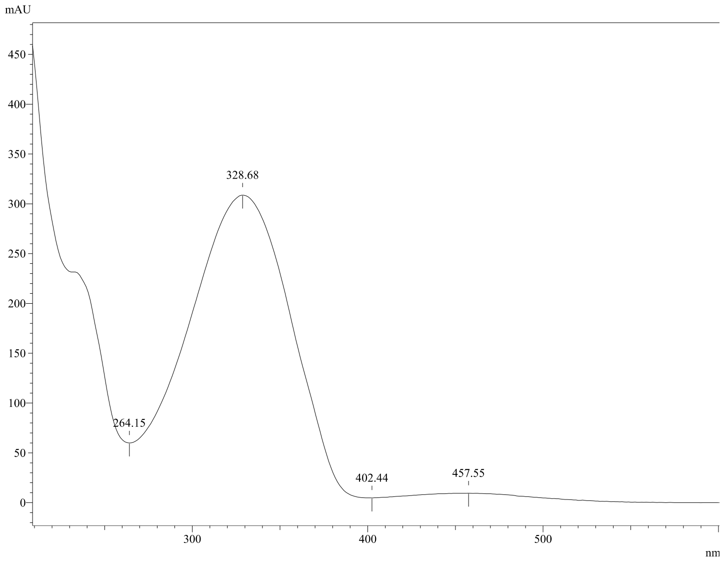

Compound **6e** (VUF16620). The iodide counter ion is visible at 0.8 min. A trace of *cis* isomer is visible at 4.62 min.

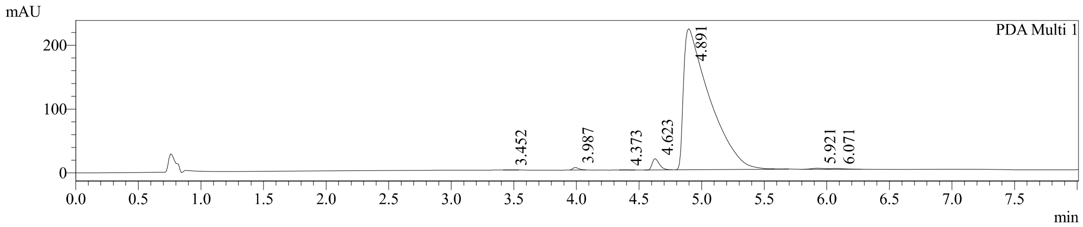

1 PDA Multi 1 / 254nm 4nm

PDA Ch1 254nm 4nm

Peak#	Name	Ret. Time	Area	Area %
1		3.452	775	0.023
2		3.987	12585	0.381
3		4.373	712	0.022
4		4.623	69984	2.117
5		4.891	3201191	96.820
6		5.921	10975	0.332
7		6.071	10114	0.306

#:1 Ret.Time:Averaged 5.020-5.040(Scan#:503-505)
Mass Peaks:14 Base Peak:580.10(122409) Polarity:Pos Segment1 - Event1

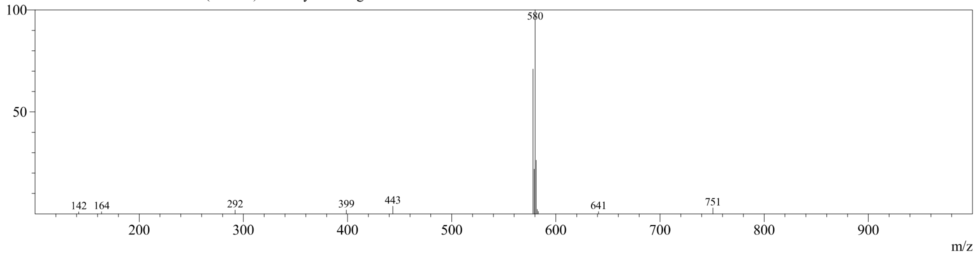

#:2 Ret.Time:Averaged 4.680-4.700(Scan#:469-471)
Mass Peaks:9 Base Peak:580.10(149159) Polarity:Pos Segment1 - Event1

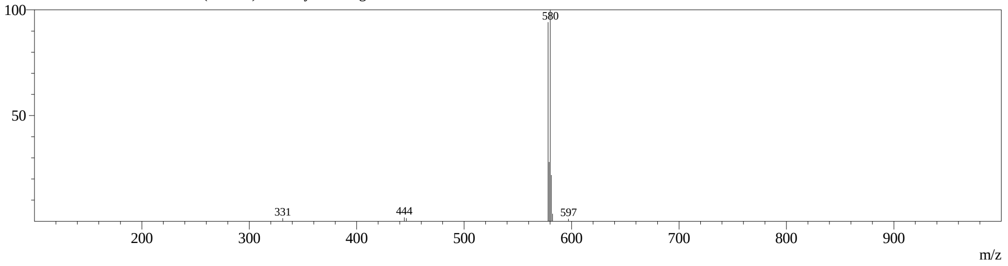

Retention Time : 4.623
Compound Name :
Spectrum Operation : BG Adjust

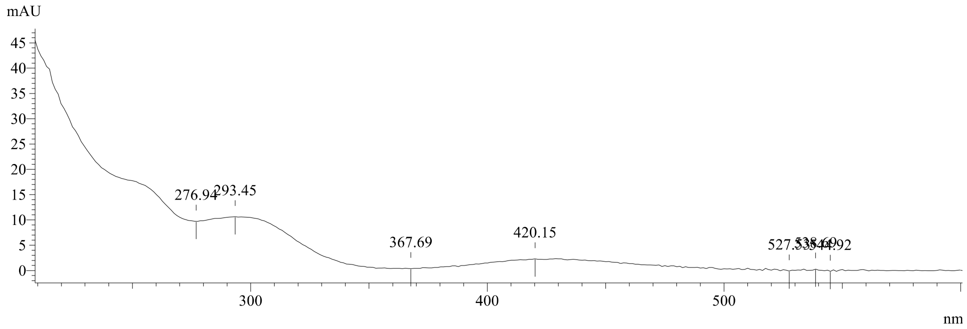

Retention Time : 4.891
Compound Name :
Spectrum Operation : BG Adjust

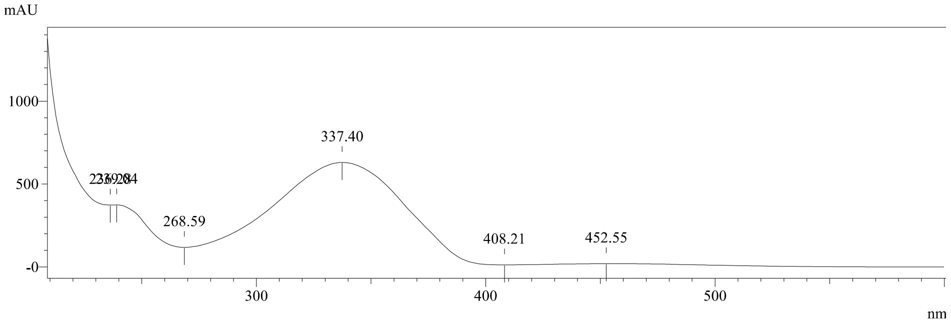

Compound **6f** (VUF16216). The iodide counter ion is visible at 0.8 min. Analytical data for this compound has been published by us before [1].

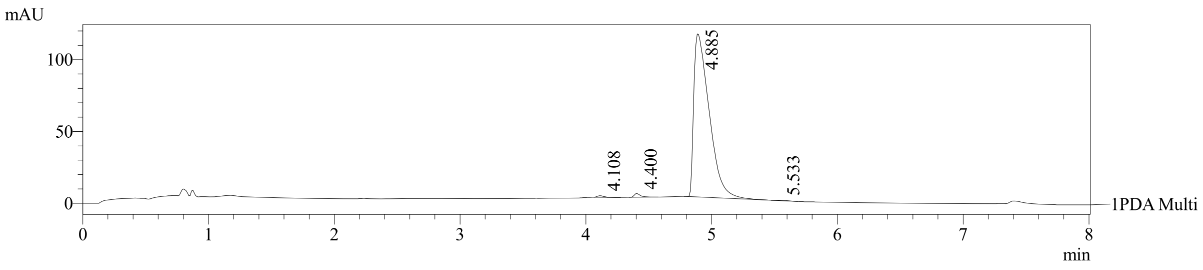

1 PDA Multi 1 / 254nm 4nm
PDA Ch1 254nm 4nm

Peak#	Name	Ret. Time	Area	Area %
1		4.108	3422	0.350
2		4.400	8828	0.903
3		4.885	963798	98.578
4		5.533	1651	0.169

#:2 Ret.Time:Averaged 4.960-4.980(Scan#:497-499)
Mass Peaks:6 Base Peak:482.25(674463) Polarity:Pos Segment1 - Event1

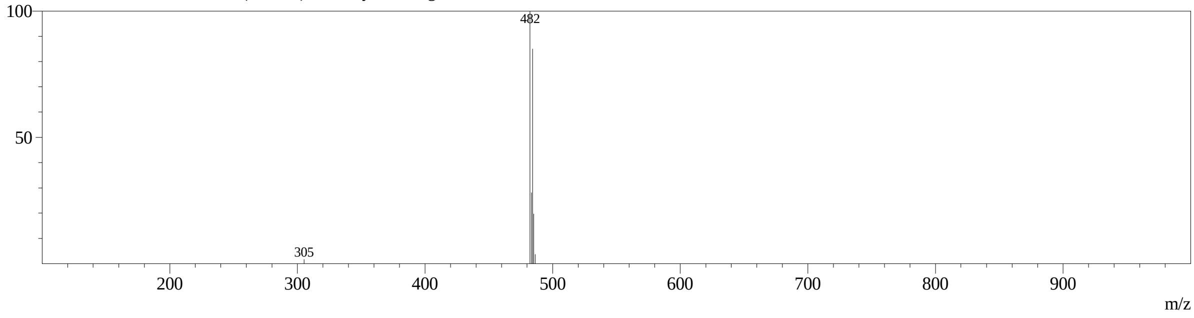

Retention Time : 4.885
Compound Name :
Spectrum Operation : BG Adjust

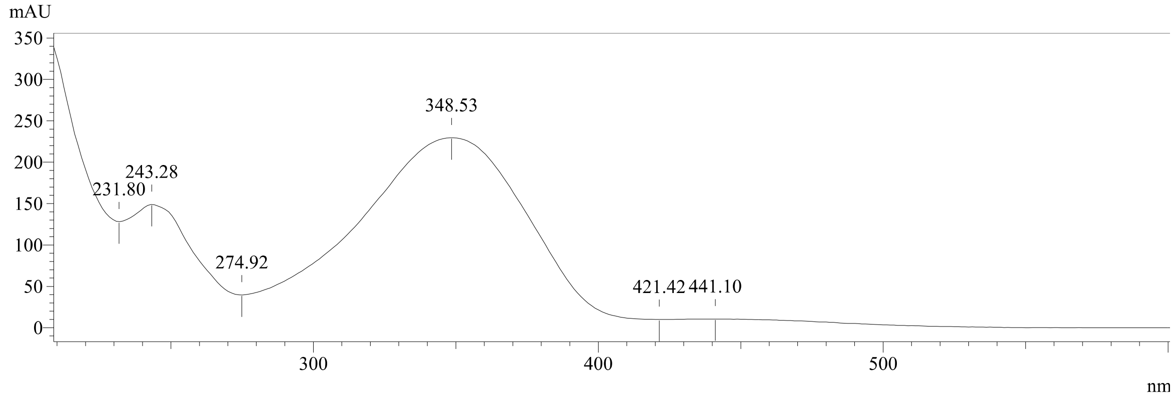

References

(1) Gómez-Santacana, X.; de Munnik, S. M.; Vijayachandran, P.; Da Costa Pereira, D.; Bebelman, J. P. M.; de Esch, I. J. P.; Vischer, H. F.; Wijtmans, M.; Leurs, R. *Angew. Chem. - Int. Ed.* **2018**, *57* (36), 11608–11612.

(2) Hauwert, N. J.; Mocking, T. A. M.; Da Costa Pereira, D.; Kooistra, A. J.; Wijnen, L. M.; Vreeker, G.; Verweij, N. W. E.; De Boer, B. H.; Smit, M. J.; de Graaf, C.; et al. *J Am Chem Soc* **2018**, *140* (12), 4232–4243.

(3) Scholten, D. J.; Wijtmans, M.; van Senten, J. R.; Custers, H.; Stunnenberg, A.; de Esch, I. J.; Smit, M. J.; Leurs, R. *Mol Pharmacol* **2015**, *87* (4), 639–648.

(4) Scholten, D. J.; Canals, M.; Wijtmans, M.; de Munnik, S.; Nguyen, P.; Verzijl, D.; de Esch, I. J.; Vischer, H. F.; Smit, M. J.; Leurs, R. *Br J Pharmacol* **2012**, *166* (3), 898–911.

(5) Yung-Chi, C.; Prusoff, W. H. *Biochem. Pharmacol.* **1973**, *22* (23), 3099–3108.

(6) Wijtmans, M.; Verzijl, D.; Bergmans, S.; Lai, M.; Bosch, L.; Smit, M. J.; de Esch, I. J.; Leurs, R. *Bioorg Med Chem* **2011**, *19* (11), 3384–3393.
